# Experimental and In Silico Evaluation of New Heteroaryl Benzothiazole Derivatives as Antimicrobial Agents

**DOI:** 10.3390/antibiotics11111654

**Published:** 2022-11-18

**Authors:** Alexander Zubenko, Victor Kartsev, Anthi Petrou, Athina Geronikaki, Marija Ivanov, Jasmina Glamočlija, Marina Soković, Lyudmila Divaeva, Anatolii Morkovnik, Alexander Klimenko

**Affiliations:** 1 North-Caucasian Zonal Research Veterinary Institute, 346406 Novocherkassk, Russia; 2 InterBioScreen, 85355 Moscow, Russia; 3 Faculty of Pharmacy, School of Health, Aristotle University, 54124 Thessaloniki, Greece; 4 Mycological Laboratory, Department of Plant Physiology, Institute for Biological Research “Siniša Stanković”, National Institute of Republic of Serbia, University of Belgrade, 11060 Belgrade, Serbia; 5 Institute of Physical and Organic Chemistry, Southern Federal University, Pr. Stachki 194/2, 344090 Rostov-on-Don, Russia

**Keywords:** heteroarylated benzothiazole, antimicrobial, antibacterial, antifungal, molecular docking

## Abstract

In this manuscript, we describe the design, preparation, and studies of antimicrobial activity of a series of novel heteroarylated benzothiazoles. A molecular hybridization approach was used for the designing compounds. The in vitro evaluation exposed that these compounds showed moderate antibacterial activity. Compound **2j** was found to be the most potent (MIC/MBC at 0.23–0.94 mg/mL and 0.47–1.88 mg/mL) On the other hand, compounds showed good antifungal activity (MIC/MFC at 0.06–0.47 and 0.11–0.94 mg/mL respectively) with **2d** being the most active one. The docking studies revealed that inhibition of *E. coli MurB* and 14-lanosterol demethylase probably represent the mechanism of antibacterial and antifungal activities.

## 1. Introduction

The growing problem in the community and in hospitals is resistance to pathogenic bacteria. Thus, the search for novel agents to fight against bacterial resistant is very attractive for scientists.

Benzothiazoles and its derivatives attracted the interest of medicinal chemists because of their extensive variety of pharmacological properties, including anti-inflammatory [1,2,3], antimicrobial [4,5,6,7], anticancer [8,9,10], antitubercular [11,12], antidiabetic [13], antioxidant [14,15], antiviral [16,17,18], antileishmanial [19], and others [20,21].

The antimicrobial potential of benzothiazoles is of great importance against the backdrop of the global aggravating problem of antimicrobial resistance and multidrug resistance that cause significant mortality in the world (about 700,000 annual deaths with the prospect of an increase in more than an order of magnitude) [10]. 

The antimicrobial activity of benzothiazole derivatives is widely presented in the literature. Thus, Singh et al. [22] synthesized and evaluated the antimicrobial activity of several novel benzothiazole based 4-thiazolidinones. Some compounds appeared to be the very potent against *E. coli* and *C. albicans* with MIC values in the range of 15.6–125 microg/mL. Haroun et.al. [5] synthesized new benzothiazole based thiazolidinone and found that all synthesized derivatives expressed better activity than ampicillin against most of the studied strains as well as more than streptomycin against several strains. On the other hand, compounds showed very good antifungal activity higher than reference drugs ketoconazole and bifonazole with very low toxicity (LD50 350–1000 mg/kg). Morsy et al. [4] evaluated antimicrobial activity of benzothiazole derivatives with MIC for antibacterial and antifungal one at 25–250 mg/mL. Nishad et al. [6] synthesized substituted N-(benzo[d]thiazol-2-yl)-2-chloroacetamides among which compound B_4_ was the most potent against all the tested strains with low MIC values. 

**Figure 1:**
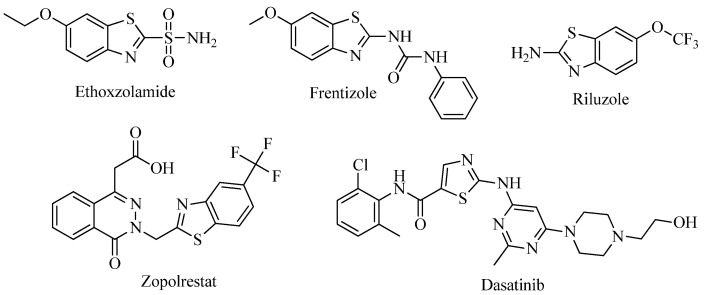
Benzothiazole- and phthalazine-based approved drugs.

It is noteworthy that there are many drugs with benzothiazole scaffold, such as Ethoxzolamide, a sulfonamide medication acting as carbonic anhydrize inhibitor against glaucoma and duodenal ulcers being a diuretic agent; Frentizole, an FDA-approved immunosuppressive drug, a novel inhibitor of the Aβ-ABAD interaction; Riluzole, a medication for the treatment of amyotrophic lateral sclerosis; and Zopolrestat, an aldose reductase inhibitor for the treatment of antidiabetic drug. [23]. Additionally, there are many benzothiazole derivatives known to be studied in clinical trials [24] (Figure 1).

On the other hand, phthalazine core is also mentioned in the literature to possess antimicrobial activity. Mourad et al. [25] prepared a series of phthalazine derivatives and studied their antimicrobial activity towards three bacterial strains. It was found that many of the compounds exhibited excellent inhibition against the tested pathogens. Rayes et al. [26] reported antimicrobial activity of phthalazinedion-based derivatives. Moreover, the antitumor approved drug Dasatinib contains phthalazine moiety. Consequently, the design and development of new benzothiazole-based phthalazine derivatives is a promising option in the creation of novel antimicrobial agents.

Taking all these into account, and as a continuation of our outgoing project on search for new compounds with antimicrobial activity, we synthesized novel derivatives incorporating benzothiazole and substituted phthalazine heterocycle through different linkers in the frame of one molecule. It is known that the combination of two or more molecules in one [27] is a promising strategy for enhancing of the activity as well as diminishing the side effects [28].

**Figure 2:**
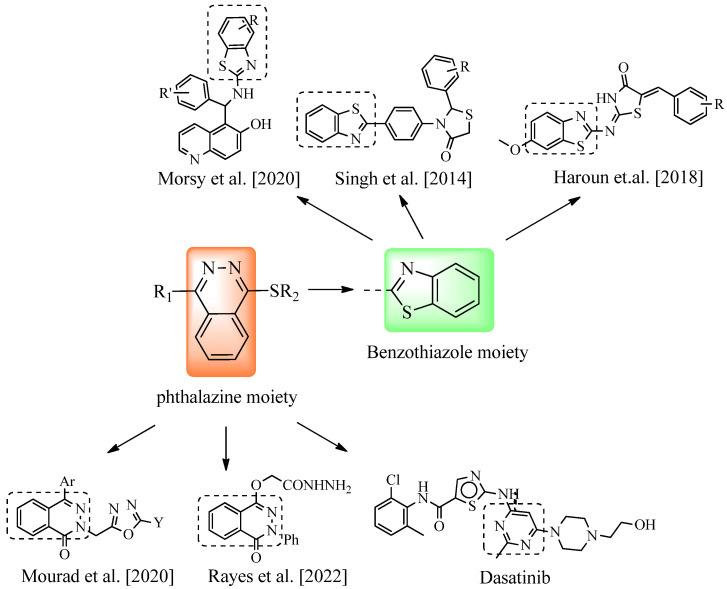
Compounds design based on bibliography [4,5,22,25,26].

Herein we report the synthesis, evaluation of antimicrobial activity, as well as molecular docking studies of new heteroarylated benzothiazole derivatives (Figure 2).

## 2. Results and Discussion

### 2.1. Chemistry

**Scheme 1:**
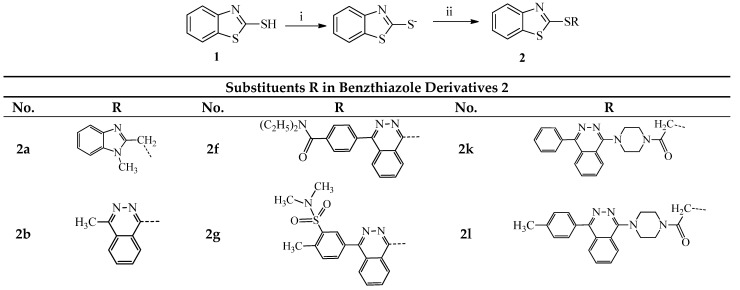

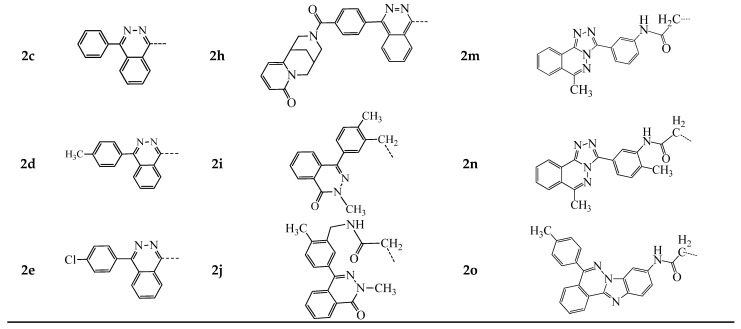
Synthesis of heteroarylderivatives benzothiazole **2**. Reagents and conditions: (i) NaH, DMF, nitrogen atmosphere, 20–25 °C, 0.5 h; (ii) 5 min, 1 h at 100 °C (for **2i**, **k**, **l**), 40–45 °C (for **2a**, **m**–**o**) or 4 h at 50–55 °C (for **2j**).

**Scheme 2:**
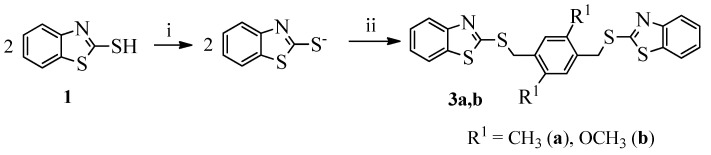
Synthesis of benzothiazole **3**. Reagents and conditions: (i) NaH, DMF, nitrogen atmosphere, 25–30 °C, 0.5 h; (ii) 15–20 °C, then at 40–45 °C, 0.5 h.

**Scheme 3:**
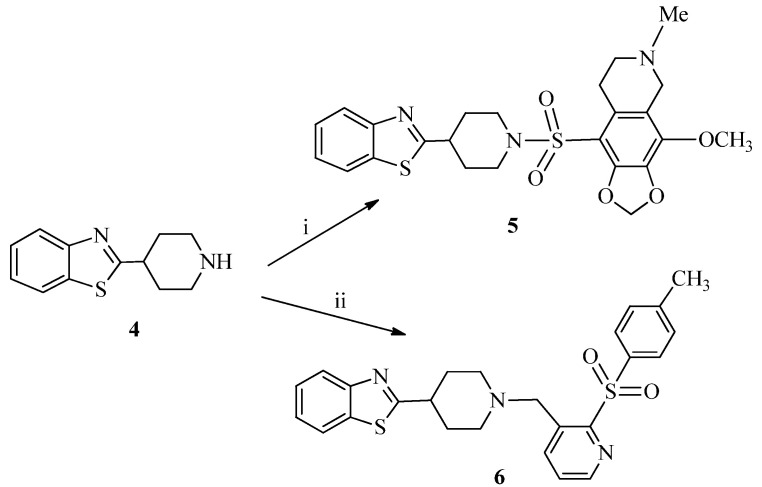
Synthesis of benzothiazole derivatives **5**, **6**. Reagents and conditions**:** (i), CHCl_3_, Et_3_N, 20–25 °C, 10 h; (ii) 2NaHCO_3_, DMF, 20–25 °C, 2 h.

**Scheme 4:**
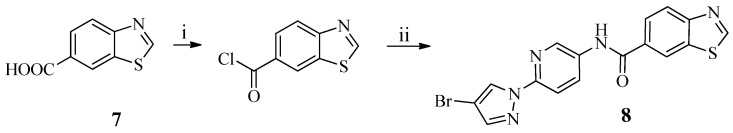
Synthesis of 5-substituted benzothiazole **8.** Reagents and conditions: (i) SOCl_2_, DMF, CHCl_3_, boiling; (ii), Et_3_N, CHCl_3_, 0–2 °C, NaHCO_3_.

**Scheme 5:**
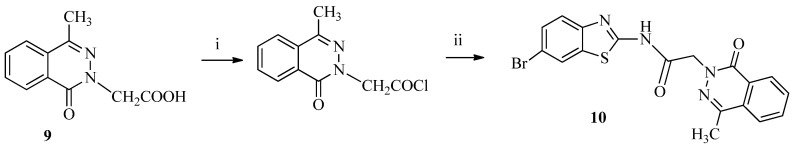
Synthesis of oxophthalazines benzothiazole derivative **10**. Reagents and conditions: (i) SOCl_2_, DMF, boiling; (ii) pyridine, 20–25 °C, NaHCO_3_.

New heteroarylated benzothiazoles were synthesized according to five routs (Scheme 1, Scheme 2, Scheme 3, Scheme 4 and Scheme 5) and their antimicrobial activity studied against a panel of pathogens.

In these benzothiazole derivatives (structures **2**, **3**, **5**, **6**, **8**, **10**) the heteroaryl group is linked to the mono- or bicyclic scaffold through various linkers such as -(CH_2_)_n_CONH(CH_2_)_2_- (n = 1,2), -S-, 1-piperidin-4-yl, -CONH-.

The target benzothiazole heteroaryl(aryl)derivatives were prepared by nucleophilic substitution of the chlorine atom in chlorosubstituted (1-methyl-2-chloromethylbenzimidazole for **2a**, 1-chloro-4-R-phthalazines for **2b**–**2o**, 1,4-bis(chloromethyl)benzene derivatives—for **3a**, **3b**, 3-(chloromethyl)-2-tosylpyridine—for **6**) or in acid chlorides (tetrahydro-[1,3]dioxolo[4,5-g]isoquinoline-8-sulfonyl chloride -for **5**), benzo[*d*]thiazole-6-carboxylic acid for **8**) and 4-methyl-1-oxophthalazine-2(1*H*)-carboxylic acid for **10**) various derivatives of benzothiazole in DMF. In this case, the clean products with good yield were obtained. Compounds were characterized by ^1^H-NMR, ^13^C-NMR, and elemental analysis. In ^1^H-NMR, the signals of the benzothiazole ring appeared in the aromatic region of 7.30–7.60 ppm (H-5, H-6), 7.88–8.78 (H-4, H-7). The signal of SCH_2_ group of compounds **2a**, **2i**–**2o**, **3a**, **3b** was found as a singlet at 4.10–4.47 ppm. In the ^13^C NMR spectra, the signal of the characteristic C=O group is seen at 166 ppm. All signals of ^1^H-NMR, ^13^C-NMR correspond to the proposed structures.

### 2.2. Biological Evaluation

#### 2.2.1. Antibacterial Activity

Title derivatives were studied for their antibacterial activity against several selected bacterial pathogens by microdilution method. Compounds showed moderate to good potency (MIC/MBC at 0.23 to >3.75 mg/mL and 0.35–>3.75 mg/mL, respectively; Table 1) following the order: **2j** > **2c** > **2g** = **8** > **2d** > **2h** > **2e** > **5** > **2i** > **2l** > **2k** > **3a** > **2a** > **2b** > **2m** > **6** > **2o** > **2n** > **3b** > **10** > **2f**.

The most potent among compounds tested appeared **2j** with MIC and MBC at 0.23–0.94 mg/mL and 0.47–1.88 mg/mL, respectively, while compound **2f** was the less potent. Some compounds demonstrate quite high potency against some bacterial strains. Thus, compounds **2b** and **2e** exhibit good activity against *S. typhimurium* with MIC 0.23 mg/mL, while compounds **2g** and **2j** exhibit good activity against *E. coli* with the same MIC. Compounds **2d**, **2k** and **8** were potent against *B. cereus* (MIC 0.23 mg/mL), whereas **2k** also exhibit good activity against *L. monocytogenes.* On the other hand, activity of compounds **2a**, **2i** and **5** against the same strain was a little bit lower (MIC 0.35 mg/mL). *En. cloacae* appeared to be very sensitive to these derivatives in the contrast to the resistant *S. aureus.*

According to structure-activity relationships studies the presence of 4-(3,4-dimethylphenyl)-2-methylphthalazin-1(2*H*)-one connected to benzothiazole via 2-mercapto-*N*-methylacetamide linker (**2j**) is beneficial for antibacterial activity. Replacement of 4-(3,4-dimethylphenyl)-2-methylphthalazin-1(2*H*)-one as substituent by 1-phenylphthalazine connected to benzothiazole by S as linker (**2c**) decreased a little the activity. Introduction of 1-phenylpthalazine *N*,*N*-dimethylsulfonic amide as substituent gave compound **2g** with less potency in comparison with **2c**. *N*,*N*-diethyl-4-(phthalazin-1-yl)benzamide as substituent was detrimental not only for the group of compounds with S-linker but for all tested compounds. From all mentioned above, it seems that the important role for antibacterial activity play the substituent of benzothiazole ring as well as the linker.

#### 2.2.2. Antifungal Activity

The evaluation of antifungal activity was performed via a microdilution method, with bifonazole and ketoconazole being used as the reference drugs. According to obtained results (Table 2) all compounds demonstrated good antifungal activity except compound **10**. The order of activity can be presented as follows: **2d** > **3b** > **3a** > **2o** > **2m** > **2a** > **2i** > **2j** > **2l** > **6** > **2b** > **5** > **8** > **2e** > **2c** > **2g** > **2f** > **2n** > **2h** > **10**. Compound **2d** with MIC/MFC at 0.08–0.17 mg/mL and 0.11–0.23 mg/m, respectively, exhibited the highest potency, whereas compound **10** was the less active.

Several derivatives appeared to be more active than the reference drugs towards some fungal strains. Thus, compounds **2d**, **2i**, **3b**, and **6** showed better potency against *T viride* compared with ketoconazole and bifonazole with MIC/MFC at 0.06/0.11 mg/mL. Good potency was also expressed in compounds **2a**, **2b**, **2h**, **2i**, **2l**, **3a**, and **5**, with minimal inhibitory and fungicidal concentrations at 0.11/0.23 mg/mL, comparable with both reference drugs against *T. viride*, while **2i**, **2m**, and **3a** were found to be active also against *A. niger*. On the other side, compounds **2a**, **2j**, **2m**, and **2o** were potent against *A. versicolor*, while **2a** was also potent with MIC at 0.11mg/mL against *P. cyclopium var. verucosum*. It should be mentioned that *T. viride* demonstrated high sensibility toward our compounds, while *A. fumigatus*, followed by *P. funiculosum.* were the most resistant ones.

The structure-activity relationship study showed that the presence of 1-(p-tolyl)phthalazine substituent linked to benzothiazole ring through S-linker (**2d**) is favorable for antifungal activity. Replacement of 1-(p-tolyl)phthalazine by 2,5-dimethoxy-1,4-phenyl linked to two benzothiazole rings via sulfamethylene linker give less active compound **3b**, while introduction of methyl group (**3a**) instead of methoxy ones (**3b**) resulted in lesser active compound compared with previous one (**3b).** It should be mentioned that ten compounds (**2b**, **2d**, **2h**, **2i**, **2j**, **2l**, **2m**, **2o**, **3a**, and **6**) showed activity better than that of ketoconazole against *A. niger* mostly, while all compounds exhibited higher activity than ketoconazole against *T. viride*. Furthermore, derivatives **2b**, **2d**, **2h**, **2i**, **2j**, **2l**, **2m**, and **6** were more potent also than bifonazole against *T. viride*. The general observation is that fungi are more sensitive to tested than bacterial strains. It should be noticed that the response of fungi and bacteria to the compounds tested is different. This behavior is probably due to some differences between bacteria and fungi organization of prokaryotic organisms, organization of DNA genetic material and finally in composition of the cell wall which are made from peptidoglycans (bacteria) and chitin (fungi). Both are prokaryotic organisms, but bacteria are unicellular, while fungi multicellular. On the other hand, despite both containing DNA as genetic material, the genetic material of bacteria is organized in cytoplasm, while in fungi it is organized inside the nucleus. Bacteria do not contain membrane-bound organelles in comparison with fungi which contain membrane-bound. Finally, the cell wall of bacteria is made up of peptidoglycans, whereas the cell wall of fungi is made up of chitin. The only common response of bacteria and fungi to compounds tested was observed for compounds **10** and **2f** which were among the less active.

### 2.3. In Silico Studies—Molecular Docking

#### 2.3.1. In Silico Studies to Antibacterial Targets

Compounds were docked to different antibacterial targets, aiming for a prediction of possible mechanisms of action.

To this direction, we used the following enzymes for docking studies: responsible for the most common mechanisms of activity of antibacterial agents such as *E. coli* DNA gyrase, Thymidylate kinase, *E. coli* Primase, *E. coli* MurA and *E. coli* MurB enzymes.

According to the results of the docking studies, the lowest Free Energy of Binding was observed to *E. coli* MurB (Table 3), suggesting inhibition of this enzyme as putative mechanism of antibacterial activity.

**Figure 3:**
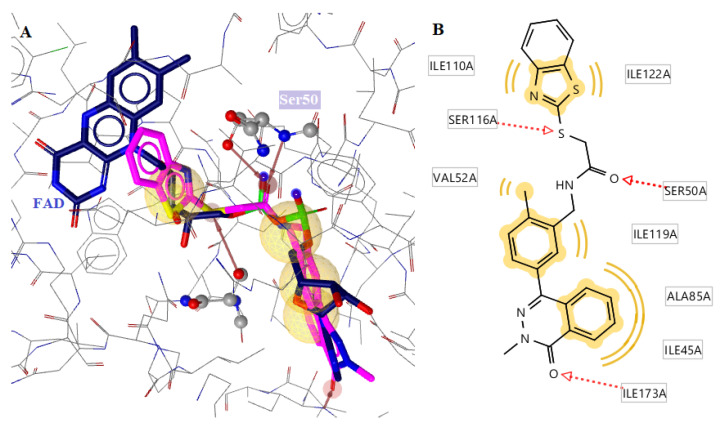
(**A**) Superposition of compound **2j** (magenta) and FAD (blue) in *E. coli* MurB. (**B**) Docked conformation of the most active compound **2j** in *E. coli* MurB. Red dotted arrows indicate H-bond and yellow spheres hydrophobic interactions.

One of the most active compounds, **2d**, binds *E. coli* MurB enzyme forming three favorable hydrogen bond interactions. These are between the oxygen atom of COOH group, of compound and residue Ser50 (2.27 Å), and the oxygen atom of the C=O group and Ile173 residue (2.74 Å), and the last one between S atom of the compound and residue Ser116 (3.56 Å). Moreover, hydrophobic interactions between Ile122, Ile110, Ile119, Val52, Ala85 and Ile45 and the compound were detected, contributing to the stability of the complex ligand-enzyme (Figure 3).

It was observed that the most active compounds bind to MurB in a similar way to FAD, interacting with the residues such as Ser50, Arg213, Arg158 and Ser229 (Figure 3). The similarity in binding mode with FAD is probably the reason of comparable to ampicillin potency of these derivatives.

**Figure 4:**
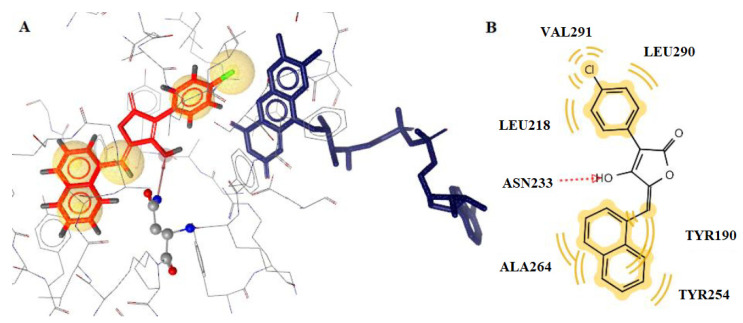
(**A**) Superposition of Naphthyl Tetronic Acid inhibitor (red) and FAD (blue) in *E. coli* MurB. (**B**) Docked conformation of Naphthyl Tetronic Acid inhibitor in *E. coli* MurB. Red dotted arrows indicate H-bond and yellow spheres hydrophobic interactions.

Finally, the docking pose of a known inhibitor of MurB enzyme also co-crystalized with it in the X-ray structure and showed that it binds MurB in a completely different way from our compounds. This inhibitor fit into the binding center of the enzyme away from the binding cavity of substate FAD, while our compounds seem to bind MurB in the FAD cavity of the enzyme, interacting with crucial for the enzyme activity residues (Figure 3 and Figure 4). This observation confirms the better binding energy of our compounds and by extension their higher inhibition over this inhibitor.

#### 2.3.2. In Silico Studies to Antifungal Targets

All the synthesized compounds and the reference drug ketoconazole were docked to lanosterol 14α-demethylase of *C. albicans* and DNA topoisomerase IV (Table 4) in order to explore the possible mechanism of antifungal activity of compounds.

It was found that the most active compound, **2d**, binds the enzyme alongside the heme group, interacting with it throughout its benzene ring forming aromatic and hydrophobic interactions.

**Figure 5:**
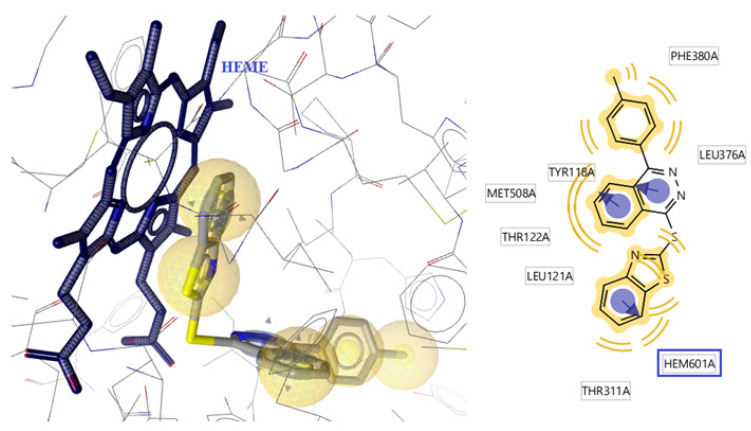
Docked conformation of the most active compound **2d** in lanosterol 14α-demethylase of *C*. *albicans* (CYP51_ca_). Blue arrows aromatic interactions and yellow spheres hydrophobic interactions.

**Figure 6:**
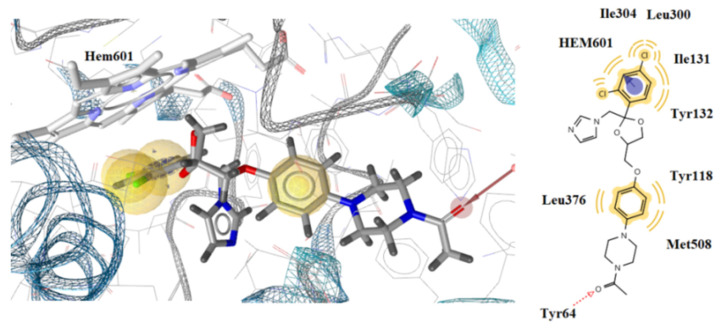
Docked conformation of ketoconazole in lanosterol 14α-demethylase of *C*. *albicans* (CYP51_ca_).

The most active compound **2d** binds the 14a-lanosterole demethylase enzyme at the side of the heme group, forming aromatic and hydrophobic interactions with its benzene ring. Moreover, hydrophobic interactions between Tyr118, Leu121, Tyr122, Thr311, Leu376, Phe380, Met508 and the compound were detected. Aromatic interaction with the heme group was also observed with the benzene ring of ketoconazole (Figure 5 and Figure 6). This property may account for the good antifungal activity of compound **2d**.

### 2.4. Drug Likeness

**Figure 7:**
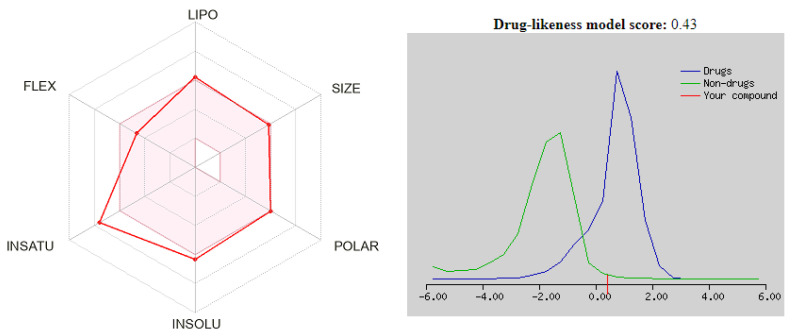
Bioavailability Radar and Drug-likeness model diagram of compound **2j**. The pink area represents the optimal range for each property for oral bioavailability, (Lipophilicity (LIPO): XLOGP3 between −0.7 and +5.0, Molecular weight (SIZE): MW between 150 and 500 g/mol, Polarity (POLAR) TPSA between 20 and 130 Å^2^, Solubility (INSOLU): log S not higher than 6, Saturation (INSATU): fraction of carbons in the sp3 hybridization not less than 0.25, and Flexibility (FLEX): no more than 9 rotatable bonds.

The bioavailability and drug-likeness scores of all compounds are shown in Table 5. According to prediction results, the bioavailability score of all compounds was about 0.55. Moreover, all compounds displayed good-to-excellent Drug-likeness scores (−0.60–0.79). Figure 7 presents the bioavailability radar of most active compound **2j**. The best in the *in-silico* predictions results was achieved for compound **5** with a Drug-likeness score of 0.79 and with no violation of any rule. According to predicted results all compounds except **2g**, **2h**, **2j**, **2m**, **2n**, **3a**, and **3b** can be orally absorbed since their TPSA are < 120 Å.

## 3. Materials and Methods

### 3.1. Chemistry-General Information

NMR ^1^H spectra of all compounds were recorded on a spectrometer Bruker 400 (400 MHz); for compounds **2a**, **2b**, **5**—on Bruker AC-300 in DMSO-*d*_6_ and spectra are presented in Supplementary Material File S1. Chemical shifts of nuclei ^1^H were measured relatively the residual signals of deuteron solvent (δ = 2.50 ppm). Coupling constants (*J*) are reported in Hz. Τhe assignment was based on 2D NMR techniques. Melting points were determined using the Fisher-Johns Melting Point Apparatus (Fisher Scientific) and are uncorrected. Elemental analysis was performed by the classical method of microanalysis. The reaction and purity of the obtained compounds were monitored by TLC (plates with Al_2_O_3_ III activity grade, eluent CHCl_3_, development of TLC plates by exposition to iodine vapors in “iodine chamber”). The solvents were purified according to standard procedures. The starting compounds—4-substituted 1-chlorophthalazine (for **2b**–**2o**)—were provided by InterBioscreen Ltd. (Russia); benzo[d]thiazole-2-thiol **1** (for **2a**, **3a**, **3b**), benzo[*d*]thiazole-6-carboxylic acid 7 (for **8**), and 4-methyl-1-oxophthalazine-2(1*H*)-carboxylic acid **9** (for **10**) are commercially available. L, 2-(Piperidin-4-yl)benzo[*d*]thiazole was obtained similarly to the procedure described in [29,30].

#### 3.1.1. General Procedure for the Synthesis of Compounds **2a**–**o** and **3a**,**b**

Sodium hydride (0.29 g, 0.012 mol) was added to a solution of 2-mercaptobenzothiazole **1** (0.01 mol) in of DMF (15 mL) with stirring in a nitrogen atmosphere at 25–30 °C. The mixture was stirred for 30 min, 2-(chloromethyl)-1-methyl-1*H*-benzo[*d*]imidazole (for **2a**) or a 4-substituted 1-chlorophthalazine (0.01 mol) was added and kept 1 min while boiling (for **2b**–**2h**), 1 h at 100 °C (for **2i**, **2k**, **2l**), 4 h at 50–55 °C (for **2j**), and 30 min at 40–45 °C (for **2m**–**2o**). Then the mixture was cooled, water (40 mL) was added, the precipitate was filtered off, and washed with water (3 × 20 mL).

*2-{[(1-Methyl-1H-benzo[d]imidazol-2-yl)methyl]thio}benzo[d]thiazole* (**2a**). Yield 2.99 g (96%), colorless crystals, m.p. 90–92 °C (EtOAc). ^1^H NMR (400 MHz, DMSO-*d*_6_, δ, ppm): 3.93 (s, 3H, Me), 4.98 (s, 2H, CH_2_), 7.14–7.25 (m, 2H, H-5′, H-6′), 7.29–7.48 (m, 3H, H-5, H-6, H-4′), 7.58 (d, *J* 7.6, 1H, H-7′), 7.85–7.88 (m, 2H, H-4, H-7). ^13^C NMR (100 MHz, DMSO-*d*_6_) δ 165.85 (S-C-S), 152.89 (C-12), 150.16 (C-4), 142.28 (C-15), 136.36 (C-14), 135.32 (C-5), 126.89 (C-8), 125.10 (C-7), 122.80 (C-18), 122.38 (C-19), 122.18 (C-6), 121.68 (C-9), 119.21 (C-17), 110.65 (C-20), 30.56 (CH_3_), 29.46. Found (%): C, 61.45; H, 4.00; N, 13.16; S, 20.72. Calc. for C_16_H_13_N_3_S_2_ (%): C, 61.71; H, 4.21; N, 13.49; S, 20.59.

*2-(4-Methylphthalazin-1-yl)benzo[d]thiazole* (**2b**). Yield 2.47 g (89%), colorless crystals, m.p. 163–165 °C (EtOH). ^1^H NMR (400 MHz, DMSO-*d*_6_, δ, ppm): 2.99 (s, 3H, Me), 7.35–7.56 (m, 2H, H-5, H-6), 8.22–8.38 (m, 2H, H-6′), 8.02–8.15 (m, 3H, H-5′, H-7′, H-8′), 8.22–8.38 (m, 2H, H-4, H-7). ^13^C NMR (100 MHz, DMSO-d_6_) δ 161.14, 159.82, 155.43, 152.07 (C-4), 139.85 (C-10), 136.05 (C-5), 134.74 (C-16), 134.33 (C-17), 132.81 (C-14), 129.65 (C-15), 127.51 (C-8), 126.19 (C-19), 125.24 (C-7), 124.48 (C-18), 122.39 (2C, C-6, C-9), 30.24 (CH_3_). Found (%): C, 68.98; H, 3.68; N, 15.00; S, 11.72. Calc. for C_16_H_11_N_3_S (%): C, 69.29; H, 4.00; N, 15.15; S, 11.56.

2-(4-*Phenylphthalazin*-*1-yl)benzo[d]thiazole* (**2c**). Yield 3.12 g (92%), colorless crystals, m.p. 214–216 °C (methycellosolve). ^1^H NMR (400 MHz, DMSO-*d*_6_, δ, ppm): 7.39–7.59 (m, 2H, H-5, H-6), 7.60–7.85 (m, 5H, H″–H-6″), 7.88–8.20, (m, 2H, 8.22–8.38 (m, 5H, H-7, H-5′–H-8′), 8.35–8.46 (m, 1H, H-4). ^13^C NMR (100 MHz, DMSO-d_6_) δ 161.10 (N=C-S), 159.83 (C-13), 155.32 (C-4), 152.01 (C-10), 139.82 (C-20), 136.03 (C-5), 134.72 (C-14), 134.33 (C-16), 132.81 (C-21), 129.60 (2C, C-25, C-6), 127.52 (C-23), 126.21 (2C, C-22, C-24), 125.23 (C-17), 331 124.29 (2C, C-7, C-8), 122.38 (2C, C-9, C-19), 121.79 (2C, C-15, C-18). Found (%): C, 74.10; H, 3.61; N, 12.11; S, 9.72. Calc. for C_21_H_13_N_3_S (%): C, 74.31; H, 3.86; N, 12.38; S, 9.45.

*2-(4-(p-Tolyl)phthalazin-1-yl)benzo[d]thiazole* (**2d**). Yield 3.11 g (88%), colorless crystals, m.p. 202–205 °C (DMF:EtOAc). ^1^H NMR (400 MHz, DMSO-*d*_6_, δ, ppm): 2.91 (s, 6H, Me, H_2_O), 7.36–7.51 (m, 4H, H-2″, H-3″, H-5″, H-6″), 7.65 (d, 2H, H-5, H-6), 7.91–8.16 (m, 5H, H-5′–H-8′, H-7), 8.31–8.42 (m, 1H, H-4). ^13^C NMR (100 MHz, DMSO-d_6_) δ 167.82 (N-C-S), 159.10 (S-C=N), 158.86 (C-14), 148.14 (C-5), 144.38 (2C, C-13, C-21), 134.09 (2C, C-4, C-24), 132.54 (C-19), 130.07 (2C, C-22, C-26), 127.22 (3C, C-1, C-2, C-3), 126.57 (3C, C-12, C-23, C-25), 124.80 (2C, C-17, C-18), 123.10 (C-6), 116.08 (C-20), 18.87 (CH_3_). Found (%):C, 74.49; H, 4.00; N, 11.48; S, 9.29.Calc. for C_22_H_15_N_3_S (%): C, 74.76; H, 4.28; N, 11.89; S, 9.07.

*2-[4-(4-Chlorophenyl)phthalazin-1-yl]benzo[d]thiazole* (**2e**). Yield 3.14 g (84%), colorless crystals, m.p. 195–196 °C (methycellosolve). ^1^H NMR (400 MHz, DMSO-*d*_6_, δ, ppm): 7.42 (td, *J* 7.5, 1.4, 1H, H-6), 7.50 (td, *J* 7.5, 1.4, 1H, H-5), 7.63–7.68 (m, 2H, H-3″, H-5″), 7.79 (d, *J* 7.5, 2H, H-5′, H-8′), 7.92–7.97 (m, 1H, H-7′), 8.02 (d, *J* 7.9, 1H, H-6), 8.03–8.17 (m, 3H, H-2″, H-6″, H-7), 8.40 (d, 1H, *J* 7.8, H-4). ^13^C NMR (100 MHz, DMSO-d_6_) δ 160.75 (N=C-S), 158.84 (C-13), 155.95 (C-4), 152.01 (C-10), 136.09 (C-20), 135.15 (C-5), 134.51 (C-Cl), 132.32 (3C, C-14, C-21, C-25), 129.21(3C, C-16, C-22, C-24), 127.30 (C-17), 127.02 (C-19), 126.01 (C-8), 125.81 (C-7), 125.08 (C-18), 124.45 (C-15), 122.47 (C-6), 122.39 (C-9). Found (%):C, 67.22; H, 3.01; Cl, 9.72; N, 11.00; S, 8.74. Calc. for C_21_H_12_ClN_3_S (%): C, 67.47; H, 3.24; Cl, 9.48; N, 11.24; S, 8.58.

*4-[4-(Benzo[d]thiazol-2-yl)phthalazin-1-yl]-N,N-diethylbenzamide* (**2f**). Yield 2.81 g (64%), colorless crystals, m.p. 180–181 °C (DMF). ^1^H NMR (400 MHz, DMSO-*d*_6_, δ, ppm): 1.24 (t, *J* 7.0, 6H, 2CH_3_), 3.45 (s, 4H, 2CH_2_), 7.36–7.43 (m, 1H, H-6), 7.44–7.51 (m, 1H, H-5), 7.59 (d, *J* 7.8, 2H, H-5′, H-8′), 7.83 (d, *J* 7.9, 2H, H-2″, H-6″), 7.95 (t, *J* 8.7, 2H, H-6′, H-7′), 8.02–8.19 (m, 3H, H-3″, H-5″, H-7), 8.35–8.42 (m, 1H, H-4). ^13^C NMR (100 MHz, DMSO-d_6_) δ 166.63 (C=O), 165.61 (S-C=N), 152.83 (C-14), 142.40 (C-27), 141.68 (C-2), 139.63 (C-17), 136.58 (C-28), 135.21 (C-5), 133.47 (C-23), 132.26 (2C, C-4, C-6), 130.31 (2C, C-3, C-7), 129.58 (C-21), 128.68 (C-18), 127.98 (C-20), 126.84 (C-31), 125.35 (C-19), 124.97 (C-22), 124.20 (C-30), 124.14 (C-29), 122.36 (C-32), 21.35 (2C, CH_3_). Found (%): C, 71.00; H, 4.82; N, 12.49; S, 7.56. Calc. for C_26_H_22_N_4_OS (%): C, 71.21; H, 5.06; N, 12.78; S, 7.31.

*5-(4-(Benzo[d]thiazol-2-yl)phthalazin-1-yl)-N,N,2-trimethylbenzenesulfonamide* (**2g**). Yield 3.41 g (74%), colorless crystals, m.p. 163–165 °C (PrOH). ^1^H NMR (400 MHz, DMSO-*d*_6_, δ, ppm): 2.74 (s, 3H, CH_3_), 2.83 (s, 6H, N(CH_3_)_2_), 7.44 (td, *J* 7.6, 1.3, 1H, H-6), 7.52 (td, *J* 7.6, 1.5, 1H, H-5), 7.69 (d, *J* 7.9, 1H, H-3″), 7.93–7.96 (m, 2H, H-6′, H-7′), 8.02–8.06 (m, 1H, H-4″), 8.09–8.18 (m, 4H, H-4, H-7, H-5′, H-8′), 8.38–8.43 (m, 1H, H-6″. ^13^C NMR (100 MHz, DMSO-*d*_6_) δ 167.20 (N-C-S), 159.42 (S-C=N), 152.60 (C-5), 146.22 (C-14), 137.35 (C-24), 137.12 (C-23), 134.93 (C-4), 133.42 (C-13), 132.63 (C-21), 132.05 (C-25), 130.50 (C-26), 128.92 (C-22), 128.43 (C-12), 128.31 (C-19), 127.66 (C-17), 126.82 (C-18), 126.58 (C-20), 126.42 (C-1), 124.74 (C-2), 121.95 (C-3), 121.02 (C-6), 36.80 (2C, N-(CH_3_)_2_), 19.88 (C-CH_3_). Found (%): C, 62.32; H, 4.12; N, 12.00; S, 14.26. Calc. for C_24_H_20_N_4_O_2_S_2_ (%): C, 62.59; H, 4.38; N, 12.16; S, 13.92.

*3-{4-[4-(Benzo[d]thiazol-2-ylthio)phthalazin-1-yl]benzoyl}-1,2,3,4,5,6-hexahydro-8H-1,5-methanopyrido[1,2-a]*[1,5]*diazocin-8-one* (**2h**). ^1^H NMR (400 MHz, DMSO-*d*_6_, δ, ppm): 2.01–2.14 (m, 2H, H-5), 2.50–2.57 (m, 1H, H-6, DMSO), 3.00 (s, 2H, H-7), 3.15–3.41 (m, 3H, H-4, H-1), 3.68–3.75 (m, 1H, H-2), 4.05–4.11 (m, 1H, H-2), 6.05 (d, 1H, H-11), 6.33 (dd, *J* 9.1, 1.3, 1H, H-9), 7.03–7.37 (m, 3H, H-5′, H-6′, H-10), 7.40–7.54 (m, 2H, H-6″, H-7″), 7.72 (d, *J* 7.8, 2H, H-5″, H-8″), 7.95 (d, *J* 8.0, 1H, H-5‴), 8.03 (d, *J* 7.9, 1H, H-3‴), 8.07–8.16 (m, 3H, H-4′, H-2‴, H-6‴), 8.40–8.42 (m, 1H, H-10). ^13^C NMR (100 MHz, DMSO-d_6_) δ 175.96 (2C, C=O, C-2), 156.54 (C=O), 153.10 (C-7), 147.82 (2C, C-14, C-19), 144.93 (C-4), 140.62 (C-27), 136.71 (C-24), 134.79 (C-21), 134.54 (C-26), 130.06 (3C, C-15, C-33, C-34), 129.18 (3C, C-31, C-32, C-39), 127.77 (2C, C-35, C-40), 126.52 (2C, C-17, C-36), 125.29 (2C, C-41, C-42), 122.75 (2C, C-28, C-38), 122.61 (C-37), 109.99 (C-25), 56.70 (C-22), 53.17 (2C, C-16, C-20), 40.65 (C-8), 32.37 (C-18), 21.58 (C-23). Found (%): C, 67.12; H, 4.20; N, 11.69; S, 11.22. Calc. for C_33_H_25_N_5_O_2_S_2_ (%): C, 67.44; H, 4.29; N, 11.92; S, 10.91.

*4-[3-(Benzo[d]thiazol-2-ylthio)-4-methylphenyl]-2-methylphthalazin-1(2H)-one* (**2i**). Yield 3.82 g (89%), colorless crystals, m.p. 111–112 °C (DMF). ^1^H NMR (400 MHz, DMSO-*d*_6_, δ, ppm): 2.57 (s, 3H, CH_3_), 3.79 (s, 3H, NCH_3_), 4.74 (s, 1H, CH_2_), 7.30–7.44 (m, 4H, H-5′, H-5″, H-6″, H-8), 7.50–7.61 (m, 2H, H-6′, H-7), 7.67 (d, *J* 7.9, 1H, H-2′), 7.71–7.82 (m, 2H, H-6, H-7′), 7.83–7.88 (m, 1H, H-9), 8.36 (d, *J* 7.9, 1H, H-7″). ^13^C NMR (100 MHz, DMSO-d_6_) δ 166.13 (C-1), 158.62 (C=O), 153.01 (C-5), 145.86 (C-18), 138.40 (C-13), 135.19 (C-6), 134.94 (C-16), 133.56 (C-12), 132.91 (C-14), 132.18 (C-28), 131.14 (C-17), 131.10 (C-27), 129.29 (C-20), 128.90 (C-29), 127.75 (C-19), 126.86 (C-26), 126.73 (C-9), 126.71 (C-15), 125.08 (C-10), 122.31 (C-8), 121.69 (C-7), 39.50 (C-25), 35.33 (C-11), 19.23 (CH_3_). Found (%): C, 66.72; H, 4.18; N, 9.53; S, 15.27. Calc. for C_24_H_19_N_3_OS_2_ (%): C, 67.11; H, 4.46; N, 9.78; S, 14.93.

*2-(Benzo[d]thiazol-2-ylthio)-N-[2-methyl-5-(3-methyl-4-oxo-3,4-dihydrophthalazin-1-yl)benzyl]acetamide* (**2j**). Yield 4.09 g (84%), colorless crystals, m.p. 201–202 °C (DMF). ^1^H NMR (400 MHz, DMSO-*d*_6_, δ, ppm): 2.41 (s, 3H, CH_3_), 3.77 (s, 3H, NCH_3_), 4.10 (s, 2H, SCH_2_), 4.43 (d, *J* 5.7, 2H, NCH_2_) 7.21–7.32 (m, 4H, H-5, H-6, H-3′, H-6″), 7.46 (d, *J* 1.7, H-7‴), 7.59–7.69 (m, 2H, H-7, H-6′), 7.72–7.77 (m, 3H, H-4, H-4, H-8″), 8.27–8.37 (m, 1H, H-5″), 8.56 (s, 1H, NH). ^13^C NMR (100 MHz, DMSO-d_6_) δ 167.17 (S-C-N), 166.46 (C=O), 158.55 (C=O), 152.66 (C-23), 146.23 (C-25), 137.36 (C-5), 137.00 (C-11), 134.95 (C-4), 133.42 (C-24), 132.65 (C-16), 132.07 (C-18), 130.51 (C-19), 128.95 (C-17), 128.42(C-3), 128.29 (C-2), 127.64 (C-30), 126.85 (C-6), 126.56 (C-31), 126.40 (C-33), 124.73 (C-32), 121.98 (C-20), 121.04 (C-21), 41.24 (CH_2_-NH), 36.81 (2C, N-CH_3_), 18.98 (C-CH_3_). Found (%): C, 64.00; H, 4.22; N, 11.18; S, 13.54. Calc. for C_26_H_22_N_4_O_2_S_2_ (%): C, 64.18; H, 4.56; N, 11.51; S, 13.18.

*2-(Benzo[d]thiazol-2-ylthio)-1-[4-(4-phenylphthalazin-1-yl)piperazin-1-yl]ethan-1-one* (**2k**). Yield 3.68 g (74%), colorless crystals, m.p. 162–163 °C (EtOH). ^1^H NMR (400 MHz, DMSO-*d*_6_, δ, ppm): 3.53 (s, 2H, 2H-2′), 3.65 (s, 2H, 2H-6′), 3.88 (s, 2H, H-3′), 3.97 (s, 2H, H-5′), 4.57 (s, 2H, SCH_2_), 7.31 (t, *J* 7.6, H-4‴), 7.42 (t, *J* 7.6, H-6), 7.51–7.60 (m, 3H, H-5, H-3‴, H-5‴), 7.62–7.69 (m, 2H, H-6″, H-7″), 7.59–7.69 (m, 2H, H-7, H-6′), 7.99–7.80 (m, 5H, H-7, H-2‴, H-6‴,H-8″), 8.22 (d, *J* 8.2, 1H, H-4). ^13^C NMR (100 MHz, DMSO-*d*_6_) δ 161.14(S-C=N), 159.82 (C=O), 155.43 (C-20), 152.07 (2C, C-5, C-23), 139.85 (C-30), 136.05 (C-4), 134.74 (C-22), 134.33 (C-33), 132.81 (2C, C-11, C-28), 129.65 (4C, C-16, C-18, C-25, C-31), 127.51 (2C, C-27, C-2), 126.19 (C26), 125.24 (2C, C-1, C-29), 124.48 (2C, C-32, C-34), 122.39 (2C, C-19, C-15), 121.56 (3C, C-3, C-6, C-21). Found (%): C, 65.00; H, 4.39; N, 13.86; S, 12.99. Calc. for C_27_H_23_N_5_OS_2_ (%): C, 65.17; H, 4.66; N, 14.07; S, 12.88.

*2-(Benzo[d]thiazol-2-ylthio)-1-(4-(4-(p-tolyl)phthalazin-1-yl)piperazin-1-yl)ethan-1-one* (**2l**). Yield 3.53 g (69%), colorless crystals, m.p. 155–156 °C (CH_3_CN). ^1^H NMR (400 MHz, DMSO-*d*_6_, δ, ppm): 3.02 (s, 3H, CH_3_), 3.51 (s, 2H, 2H-2′), 3.63 (s, 2H, 2H-6′), 3.87 (s, 2H, H-3′), 3.97 (s, 2H, H-5′), 4.57 (s, 2H, SCH_2_), 7.27–7.47 (m, 4H, H-3‴, H-4‴, H-5‴, H-6‴), 7.52–7.58 (m, 2H, H-5, H-6), 7.78–8.01 (m, 5H, H-7, H-5″–H-8″), 8.21 (d, *J* 8.1, 1H, H-4). ^13^C NMR (100 MHz, DMSO-d_6_) δ 166.50 (S-C-S), 165.94 (C=O), 155.37 (C-10), 152.95 (C-13), 147.79 (C-31), 139.59 (C-22), 135.21 (C-20), 134.37 (C-32), 131.49 (C-14), 129.79 (C-16), 127.77 (2C, C-24, C-25), 126.85 (C-17), 124.98 (C-18), 123.32 (C-15), 122.96 (C-35), 122.32 (3C, C-21, C-23, C-19), 121.51 (2C, C-33, C-34), 118.48 (C-36), 38.24 (4C, C-4, C-6, C-8, C-9), 20.20 (2C, C-2, CH_3_). Found (%): C, 65.48; H, 4.69; N, 13.46; S, 12.74. Calc. for C_28_H_25_N_5_OS_2_ (%): C, 65.73; H, 4.93; N, 13.69; S, 12.53.

*2-(Benzo[d]thiazol-2-ylthio)-N-(3-(6-methyl-[1,2,4]triazolo[3,4-a]phthalazin-3-yl)phenyl)acetamide* (**2m**). Yield 3.74 g (72%), colorless crystals, m.p. 235–237 °(DMFA). ^1^H NMR (400 MHz, DMSO-*d*_6_, δ, ppm): 2.91 (s, 3H, Me), 4.38 (s, 2H, CH_2_), 7.27–7.36 (m, 1H, H-4″), 7.37–7.51 (m, 2H, H-5, H-6), 7.80–7.92 (m, 4H, H-7′–H-10′), 8.00 (t, *J* 7.6, 1H, H-5″), 8.17 (d, *J* 7.9, 2H, H-4, H-7), 8.61 (d, *J* 7.9, 1H, H-6″), 8.67 (d, *J* 2.0, 1H, H-2″), 10.48 (s, 1H, NH). ^13^C NMR (100 MHz, DMSO-d_6_) δ 166.50 (S-C=N), 165.95 (C=O), 155.37 (C-27), 152.95 (C-11), 147.79 (C-CH3), 143.79 (N-C-N), 139.58 (C-18), 135.21 (C-28), 134.37 (C-2), 131.49 (C-1), 129.79 (C-4), 127.77 (C-16), 127.43 (C-14), 126.85 (C-3), 124.98 (C-31), 123.32 (2C, C-6, C-15), 122.97 (C-17), 122.90 (C-5), 122.33 (C-32), 121.51 (C-33), 121.02 (C-30), 118.48 (C-19), 38.23 (CH_2_-C=O), 20.21 (CH_3_). Found (%): C, 62.00; H, 3.41; N, 17.21; S, 13.04. Calc. for C_25_H_18_N_6_OS_2_ (%): C, 62.22; H, 3.76; N, 17.41; S, 13.29.

*2-(Benzo[d]thiazol-2-ylthio)-N-(2-methyl-5-(6-methyl-[1,2,4]triazolo[3,4-a]phthalazin-3-yl)phenyl)acetamide* (**2n**). Yield 3.87 g (78%), colorless crystals, m.p. 251–253 °C (DMFA). ^1^H NMR (400 MHz, DMSO-*d*_6_, δ, ppm): 2.36 (s, 3H, Me), 2.87 (s, 6H, Me, DMSO), 4.39 (s, 2H, CH_2_), 7.32–7.42 (m, 3H, H-5, H-6, H-5″), 7.84–7.89 (m, 3H, H-7, H-8′, H-9′), 7.96–8.03 (m, 1H, H-7), 8.13–8.18 (m, 2H, H-7′, H-10′), 8.61 (d, *J* 8.0, 1H, H-4″), 8.69 (s, 1H, H-2″), 9.67 (s, 1H, NH). ^13^C NMR (100 MHz, DMSO-d_6_) δ 166.53 (S-C=N), 165.92 (C=O), 155.35 (C-27), 152.92 (C-11), 147.80 (C-7), 143.77 (C-10), 139.56 (C-18), 135.18 (C-28), 134.35 (C-16), 131.46 (C-17), 129.80 (C-2), 127.76 (C-1), 127.32 (C-4), 126.91 (C-14), 124.95 (C-3), 123.30 (C-31), 122.99 (2C, C-6, C-32), 122.91 (C-15), 122.34 (C-5), 121.50 (C-33), 121.07 (C-30), 118.46 (C-19), 38.24 (C-23), 24.51 (CH_3_), 20.19 (CH_3_). Found (%): C, 62.56; H, 4.40; N, 17.11; S, 13.15. Calc. for C_26_H_20_N_6_OS_2_ (%): C, 62.88; H, 4.06; N, 16.92; S, 12.91.

*2-(Benzo[d]thiazol-2-ylthio)-N-(5-(p-tolyl)benzo[4,5]imidazo[2,1-a]phthalazin-9-yl)acetamide* (**2o**). Yield 4.68 g (88%), colorless crystals, m.p. 248–250 °C (DMFA). ^1^H NMR (400 MHz, DMSO-*d*_6_, δ, ppm): 2.52 (s, 3H, Me), 4.40 (s, 2H, CH_2_), 7.33–7.44 (m, 3H, H-3″, H-5″, H-10′), 7.57–7.69 (m, 3H, H-2″, H-6″, H-11′), 7.76–8.03 (m, 6H, H-5, H-6, H-1′–H-4′), 8.26 (d, *J* 1.8, 1H, H-8′), 8.74–8.78 (m, 2H, H-4, H-7), 10.47 (s, 1H, NH). ^13^C NMR (100 MHz, DMSO-d_6_) δ 166.59 (S-C=N), 165.60 (2C, N=C-N, C=O), 152.98 (C-33), 142.42 (C-8), 141.68 (C-16), 139.63 (C-24), 136.57 (C-17), 135.21 (C-19), 133.47 (C-34), 132.25 (C-22), 130.20 (2C, C-23, C-25), 129.61 (2C, C-26, C-27), 128.68 (C-11), 127.97 (C-12), 126.86 (C-9), 125.35 (C-10), 124.97 (C-13), 124.21 (C-36), 124.12 (C-37), 122.35 (C-20), 121.51 (C-35), 116.21 (C-38), 111.51 (C-14), 109.66 (2C, C-18, C-21), 38.34(CH_2_-C=O), 21.44 (CH_3_). Found (%): C, 67.42; H, 4.21; N, 13.54; S, 12.36. Calc. for C_30_H_21_N_5_OS_2_ (%): C, 67.77; H, 3.98; N, 13.17; S, 12.06.

*2,2′-{[(2,5-Dimethyl-1,4-phenylene)bis(methylene)]bis(sulfanediyl)}bis(benzo[d]thiazole)* (**3a**). Yield 90%, colorless crystals, m.p. 170–171 °C (methylcellosolve), ^1^H NMR (200 MHz, DMSO-*d*_6_, δ, ppm): 2.32 (s, 6H, 2Me), 4.54 (s, 4H, 2CH_2_), 7.26 (s, 2H, H-3″, H-5″), 7.34–7.37 (m, 2H, H-5, H-5′), 7.43–7.47 (m, 2H, H-6, H-6′), 7.86 (d, *J* 1.1, H-7), 7.88 (d, *J* 1.1, H-7′), 7.92 (d, *J* 1.3, H-4), 7.93 (d, *J* 1.3, H-4′). ^13^C NMR (100 MHz, DMSO-d_6_) δ 166.60 (S-C=N), 165.72 (S-C-S), 152.92 (C-5), 142.40 (C-23), 141.62 (C-14), 136.56 (C-17), 135.23 (C-4), 133.24 (C-22), 130.20 (2C, C-13, C-16), 129.63 (2C, C-12, C-15), 127.97 (C-1), 126.86 (C-2), 125.35 (C-27), 124.97 (C-26), 124.23 (2C, C-3, C-25), 124.11 (2C, C-6, C-28), 37.15 (2C, C-11, C-18), 21.42 (2C, CH_3_). Found (%): C, 62.36; H, 4.56; N, 6.34; S, 27.81. Calc. for C_24_H_20_N_2_S_4_ (%): C, 62.03; H, 4.34; N, 6.03; S, 27.60.

*2,2′-{[(2,5-Dimethoxy-1,4-phenylene)bis(methylene)]bis(sulfanediyl)}bis(benzo[d]thiazole)* (**3b**). Yield 95%, colorless crystals, m.p. 184–186 °C (DMFA). ^1^H NMR (400 MHz, DMSO-*d*_6_, δ, ppm): 3.79 (s, 6H, 2OMe), 4.56 (s, 4H, 2CH_2_), 7.18 (s, 2H, H-3″, H-6″), 7.30–7.36 (m, 2H, H-5, H-5′), 7.41–7.47 (m, 2H, H-6, H-6′), 7.84–7.92 (m, 4H, H-4, H-4′, H-7, H-7′). ^13^C NMR (100 MHz, DMSO-d_6_) δ 166.59 (S-C=N), 166.45 (S-C-S), 159.14 (2C, C-OCH_3_), 152.66 (C-5), 148.82 (C-23), 137.53 (C-4), 136.67 (C-22), 133.48 (C-15), 132.65 (C-12), 132.05 (2C, C-1, C-17), 128.44 (2C, C-2, C-26), 127.65 (2C, C-3, C-25), 124.78 (2C, C-6, C-28), 121.05 (2C, C-13, C-16), 53.28 (2C, O-CH_3_), 36.84 (2C, C-11, C-18). Found (%): C, 58.40; H, 4.29; N, 5.26; S, 25.49. Calc. for C_24_H_20_N_2_O_2_S_4_ (%): C, 58.04; H, 4.06; N, 5.64; S, 25.82.

#### 3.1.2. Synthesis of 9-((4-(Benzo[d]thiazol-2-yl)piperidin-1-yl)sulfonyl)-4-methoxy-6-methyl-5,6,7,8-tetrahydro-[1,3]dioxolo[4,5-g]isoquinoline (**5**)

Compound **5** was obtained from 2-(piperidin-4-yl)benzo[*d*]thiazole **4** and 4-methoxy-6-methyl-5,6,7,8-tetrahydro-[1,3]dioxolo[4,5-g]isoquinoline-9-sulfonyl chloride according to a modified procedure [31].

Yield 2.71 (54%), colorless crystals, m.p. 164–166 °C (EtOAc). ^1^H NMR (400 MHz, DMSO-*d*_6_, δ, ppm): 1.81–1.96 (m, 2H, 1H-3″, 1H-5″), 2.17–2.26 (m, 2H, 1H-3″, 1H-5″), 2.35 (s, 3H, Me), 2.84–2.93 (m, 2H, H-7), 3.01–3.10 (m, 5H, H-2″, H-4″, H-6″), 3.32 (s, 2H, H-8), 3.78–3.82 (m, 2H, H-5), 4.06 (s, 3H, OMe), 6.05 (s, 2H, H-2), 7.33–7.48 (m, 2H, H-5′, H-6′), 7.88–7.96 (m, 2H, H-4′, H-7′). ^13^C NMR (100 MHz, DMSO-d6) δ 169.34 (N=C-S), 168.86 (C-O), 159.42 (C-26), 145.81 (C-3), 144.37 (2C, C-2, C-25), 130.18 (2C, C-29, C-30), 119.22 (2C, C-28, C-31), 113.57 (2C, C-4, C-5), 112.99 (C-6), 94.14 (O-C-O), 74.04 (C-34), 40.90 (3C, C-7, C-9, C-35), 36.65 (2C, C-18, C-22), 31.11 (C-20), 28.15 (2C, C-19, C-21), 19.54 (C-10). Found (%): C, 57.18; H, 5.29; N, 8.15; S, 12.41. Calc. for C_30_H_21_N_5_OS_2_ (%): C, 57,47; H, 5.43; N, 8.38; S, 12.78.

#### 3.1.3. Synthesis of 2-{1-[(2-Tosylpyridin-3-yl)methyl]piperidin-4-yl}benzo[d]thiazole (**6**)

A mixture of 2-(piperidin-4-yl)benzo[*d*]thiazole **4** (2.18 g, 0.01 mol), 3-(chloromethyl)-2-tosylpyridine (2.82g, 0.01 mol), and NaHCO_3_ (1.68 g, 0.02 mol) in DMF (20 mL) was stirred 24 h at 20–25 °C, water (40 mL) was added, the precipitate was filtered off and washed with water (3 × 15 mL).

Yield 3.61 g (78%), colorless crystals, m.p. 153–155 °C (EtOAc). ^1^H NMR (400 MHz, DMSO-*d*_6_, δ, ppm): 1.90–1.98 (m, 2H, 1H-3‴, 1H-5‴), 2.11–2.16 (m, 2H, 1H-3‴, 1H-5‴), 2.33–2.40 (m, 2H, 1H-2‴, 1H-6‴), 2.46 (s, 3H, Me), 2.93–2.97 (m, 2H, 1H-2‴, 1H-6‴), 3.10–3.22 (m, 1H, H-4‴), 4.07 (s, 2H, CH_2_), 4.34–4.48 (m, 4H, H-5, H-6, H-3′, H-5′), 7.54–7.58 (m, 1H, H-5″), 7.80–7.86 (m, 2H, H-2′, H-6′), 7.89–7.94 (m, 2H, H-4, H-7), 8.22 (d, *J* 7.7, 1H, H-4″), 8.38–8.42 (m, 1H, H-6″). ^13^C NMR (100 MHz, DMSO-d6) δ 175.96 (S-C-N), 156.54 (C=N), 153.10 (C-5), 147.82 (C-20), 144.93 (C-29), 140.62 (C-26), 136.71 (C-4), 134.79 (C-18), 134.54 (C-17), 130.06 (2C, C-28, C-30), 129.18 (2C, C-27, C-31), 127.77 (C-1), 126.52 (C-2), 125.29 (C-19), 122.75 (C-6), 122.61 (C-3), 56.70 (C-16), 53.17 (2C, C-12, C-14), 40.65 (C-10), 32.37 (2C, C-11, C-15), 21.58 (CH_3_). Found (%): C, 64.48; H, 5.16; N, 9.15; S, 13.60. Calc. for C_25_H_25_N_3_O_2_S_2_ (%): C, 64.77; H, 5.44; N, 9.06; S, 13.83.

#### 3.1.4. Synthesis of *N*-[6-(4-Bromo-1*H*-pyrazol-1-yl)pyridin-3-yl]benzo[d]thiazole-6-carboxamide (**8**)

A mixture of benzo[d]thiazole-6-carboxylic acid **7** (1.80 g, 0.01 mol), SOCl_2_ (1.43 g, 0.87 mL, 0.012 mol), CHCl_3_ (20 mL) and DMF (0.05 mL) was refluxed until gas evolution stops, cooled, and the resulting solution of benzo[*d*]thiazole-6-carbonyl chloride was added dropwise at 0 °C to the solution of 6-(4-bromo-1*H*-pyrazol-1-yl)pyridin-3-amine (2.39 g, 0.01 mol) in CHCl_3_ (20 mL) and Et_3_N (2.02 g, 2.79 mL, 0.02 mol). In 10 min, a solution of NaHCO_3_ (2.52 g, 0.03 mol) and water (50 mL) were added. The organic layer is separated and dried, and the solvent is distilled off in a vacuum at 35–40 °C. Furthermore, precipitate was filtered off and washed with water (3 × 15 mL).

Yield 3.12 g (78%), colorless crystals, m.p. 247–248 °C (EtOH). ^1^H NMR (400 MHz, DMSO-*d*_6_, δ, ppm): 7.74 (s, 1H, H-2′), 7.91 (d, *J* 8.9, 1H, H-4′), 8.17–8.21 (m, 2H, H-4, H-5), 8.43 (dd, *J* 8.9, 2.5, 1H, H-5′), 8.62 (s, 1H, H-7), 8.78 (s, 1H, H-5″), 8.88 (d, *J* 2.5, 1H, H-3″), 9.46 (d, *J* 1.6, 1H, H-2), 10.58 (s, 1H, NH). ^13^C NMR (100 MHz, DMSO-d6) δ 165.81(C=O), 159.91 (S-CH2-N), 155.48 (C-5), 146.32 (C-16), 142.53 (C-22), 140.24 (C-14), 135.33 (C-4), 134.20 (C-20), 131.74 (C-13), 131.25 (C-2), 127.53 (C-18), 126.22 (C-1), 123.32 (C-3), 123.23 (C-6), 112.24 (C-17), 96.09 (C-Br). Found (%): C, 48.32; H, 2.68; Br + S, 27.64; N, 17.21. Calc. for C_16_H_10_BrN_5_OS (%): C, 48.01; H, 2.52; Br, 19.96; N, 17.50; S, 8.01.

#### 3.1.5. Synthesis of *N*-(6-Bromobenzo[d]thiazol-2-yl)-2-(4-methyl-1-oxophthalazin-2(1H)-yl) acetamide (**10**)

A mixture of 2-(4-methyl-1-oxophthalazin-2(1*H*)-yl)acetic acid **9** (2.18 g, 0.01 mol), SOCl_2_ (1.43 g, 0.87 mL, 0.012 mol), CHCl_3_ (15 mL) and DMF (0.05 mL) was refluxed until gas evolution stops, cooled and the resulting solution of 2-(4-methyl-1-oxophthalazin-2(1*H*)-yl)acetyl chloride was added dropwise at 0 °C to a solution of 6-bromobenzo[*d*]thiazol-2-amine (2.29 g, 0.01 mol) in DMF (15 mL) and piridine (2.23 mL, 0.03 mol). Then the mixture was stirred 0.5 h, NaHCO_3_ (9.5 g), water (100 mL) and petroleum (20 mL) were added. The precipitate was filtered off and washed with water (3 × 15 mL).

Yield 2.23 g (52%), colorless crystals, m.p. 271–272 °C (DMF). ^1^H NMR (400 MHz, DMSO-*d*_6_, δ, ppm): 2.60 (s, 3H, CH_3_), 5.07 (s, 1H, CH_2_), 7.51 (dd, *J* 8.6, 2.0, 1H, H-4), 7.65 (d, *J* 8.6, 1H, H-5), 7.83–7.88 (m, 1H, H-7′), 7.93–7.95 (m, 1H, H-6′), 8.09 (d, *J* 2.0, 1H, H-7), 8.32 (d, *J* 7.7, H-8′), 12.65 (s, 1H, NH). ^13^C NMR (100 MHz, DMSO-d_6_) δ 167.85 (C=O), 159.11 (C=O), 158.87 (C-8), 148.14 (C-5), 144.39 (C-17), 134.09 (2C, C-4, C-24), 130.07 (C-18), 127.22 (2C, C-1, C-19), 126.57 (2C, C-23, C-26), 124.80 (2C, C-3, C-25), 116.08 (2C, C-2, C-6), 54.17 (CH_2_-C=O), 18.87(CH_3_). Found (%): C, 50.12; H, 2.86; Br + S, 26.31; N, 13.05. Calc. for C_18_H_13_BrN_4_O_2_S (%): C, 50.36; H, 3.05; Br, 18.61; N, 13.05; S, 7.47.

### 3.2. Biological Evaluation

#### 3.2.1. Antibacterial Activity

The following Gram-negative bacteria: *Escherichia coli* (ATCC 35210), *Enterobacter cloacae* (clinical isolate), *Salmonella typhimurium* (ATCC 13311), as well as Gram-positive bacteria: *Listeria monocytogenes* (NCTC 7973), *Bacillus cereus* (clinical isolate), and *Staphylococcus aureus* (ATCC 6538) were used. The organisms were obtained from the Mycological Laboratory, Department of Plant Physiology, Institute for Biological Research “Siniša Stankovic”, Belgrade, Serbia. The minimum inhibitory (MIC) and minimum bactericidal (MBC) concentrations were determined by the modified microdilution method as previously reported [32,33].

#### 3.2.2. Antifungal Activity

The evaluation of the antifungal activity against the fungi used was performed as detailed described earlier [5,34,35].

### 3.3. Docking Studies

AutoDock 4.2^®^ software was used for the in silico studies and detailed procedure is reported in our previous paper [36].

### 3.4. Drug Likeness

Five filters were used to predict Drug-likeness [37,38,39,40] by the Molsoft software (San Diego, CA, USA) and SwissADME program (http://swissadme.ch, accessed on 25 October 2022) via the ChemAxon’s Marvin JS structure drawing tool.

## 4. Conclusions

This work presents the synthesis and study of antibacterial and antifungal activities against a panel of bacterial and fungal pathogens of twenty-one new benzohiazole derivatives. The antibacterial activity of tested compounds revealed that they have moderate activity with minimal inhibitory concentration being 0.23–2.5 mg/mL and minimal bactericidal at 0.47–0.75 mg/mL. Compounds appeared to be very active against *En. cloacae* but not against *S. aureus.*

All compounds exhibited good antifungal potency, with an MIC in range of 0.06–0.47 mg/mL and MFC at 0.11–0.94 mg/mL. Compound **2d** demonstrated the best activity among all tested with MIC/MFC at 0.008–0.17/0.11–0.23 mg/mL, respectively. The most sensitive fungal to compounds tested was *T. viride*, while *A. fumigatus* was the most resistant one. The behavior of bacteria and fungi toward our compounds was different probably due to the differences in organization of their genetic material as well as a consistence of the cell wall.

According to docking results it seems that inhibition of the MurB enzyme is a putative mechanism of antibacterial activity, whereas inhibition of CYP51 reductase is suggested to be responcible for antifungal activity of the compounds.

## Figures and Tables

**Table 1:** Antibacterial activity of heteroaryl derivatives of benzothiazole (mg/mL).

No.		*S.a.*	*B.c.*	*L.m.*	*E.c.*	*S.t.*	*En.cl.*
**2a**	MIC	1.88 ± 0.00	0.94 ± 0.00	0.94 ± 0.00	0.35 ± 0.08	0.70 ± 0.19	0.70 ± 0.19
	MBC	3.75 ± 0.00	1.88 ± 0.00	1.88 ± 0.00	0.47 ± 0.00	0.94 ± 0.00	0.94 ± 0.00
**2b**	MIC	1.41 ± 0.38	0.47 ± 0.00	0.47 ± 0.00	>3.75	0.23 ± 0.00	0.47 ± 0.00
	MBC	1.88 ± 0.00	0.94 ± 0.00	0.94 ± 0.00	>3.75	0.47 ± 0.00	0.94 ± 0.00
**2c**	MIC	0.70 ± 0.19	0.70 ± 0.19	0.70 ± 0.19	0.70 ± 0.19	0.70 ± 0.19	0.70 ± 0.19
	MBC	0.94 ± 0.00	0.94 ± 0.00	0.94 ± 0.00	0.94 ± 0.00	0.94 ± 0.00	0.94 ± 0.00
**2d**	MIC	1.41 ± 0.38	0.35 ± 0.08	0.70 ± 0.19	0.70 ± 0.19	0.70 ± 0.19	0.70 ± 0.19
	MBC	1.88 ± 0.00	0.47 ± 0.00	0.94 ± 0.00	0.94 ± 0.00	0.94 ± 0.00	0.94 ± 0.00
**2e**	MIC	1.41 ± 0.38	0.94 ± 0.00	0.70 ± 0.19	0.47 ± 0.00	0.23 ± 0.00	0.47 ± 0.00
	MBC	1.88 ± 0.00	1.88 ± 0.00	0.94 ± 0.00	0.94 ± 0.00	0.47 ± 0.00	0.94 ± 0.00
**2f**	MIC	2.50 ± 0.88	1.41 ± 0.38	0.94 ± 0.00	0.70 ± 0.19	1.41 ± 0.38	2.50 ± 0.88
	MBC	3.75 ± 0.00	1.88 ± 0.00	1.88 ± 0.00	0.94 ± 0.00	1.88 ± 0.00	3.75 ± 0.00
**2g**	MIC	1.41 ± 0.38	0.70 ± 0.19	0.70 ± 0.19	0.23 ± 0.00	0.70 ± 0.19	0.70 ± 0.19
	MBC	1.88 ± 0.00	0.94 ± 0.00	0.94 ± 0.00	0.47 ± 0.00	0.94 ± 0.00	0.94 ± 0.00
**2h**	MIC	0.94 ± 0.00	0.70 ± 0.19	0.70 ± 0.19	0.70 ± 0.19	0.47 ± 0.00	0.70 ± 0.19
	MBC	1.88 ± 0.00	0.94 ± 0.00	0.94 ± 0.00	0.94 ± 0.00	0.94 ± 0.00	0.94 ± 0.00
**2i**	MIC	1.41 ± 0.38	0.70 ± 0.19	0.70 ± 0.19	0.35 ± 0.08	0.70 ± 0.19	0.94 ± 0.00
	MBC	1.88 ± 0.00	0.94 ± 0.00	0.94 ± 0.00	0.47 ± 0.00	0.94 ± 0.00	1.88 ± 0.00
**2j**	MIC	0.94 ± 0.00	0.47 ± 0.00	0.47 ± 0.00	0.23 ± 0.00	0.35 ± 0.08	0.47 ± 0.00
	MBC	1.88 ± 0.00	0.94 ± 0.00	0.94 ± 0.00	0.47 ± 0.00	0.47 ± 0.00	0.94 ± 0.00
**2k**	MIC	0.94 ± 0.00	0.23 ± 0.00	0.35 ± 0.08	>3.75	>3.75	0.23 ± 0.00
	MBC	1.88 ± 0.00	0.47 ± 0.00	0.47 ± 0.00	>3.75	>3.75	0.47 ± 0.00
**2l**	MIC	1.41 ± 0.38	0.94 ± 0.00	0.70 ± 0.19	0.70 ± 0.19	0.70 ± 0.19	0.94 ± 0.00
	MBC	1.88 ± 0.00	1.88 ± 0.00	0.94 ± 0.00	0.94 ± 0.00	0.94 ± 0.00	1.88 ± 0.00
**2m**	MIC	1.41 ± 0.38	>3.75	0.70 ± 0.19	0.47 ± 0.00	0.35 ± 0.08	0.70 ± 0.19
	MBC	1.88 ± 0.00	>3.75	0.94 ± 0.00	0.94 ± 0.00	0.47 ± 0.00	0.94 ± 0.00
**2n**	MIC	1.41 ± 0.38	0.70 ± 0.19	0.70 ± 0.19	>3.75	0.47 ± 0.00	0.70 ± 0.19
	MBC	1.88 ± 0.00	0.94 ± 0.00	0.94 ± 0.00	>3.75	0.94 ± 0.00	0.94 ± 0.00
**2o**	MIC	1.41 ± 0.38	0.70 ± 0.19	0.70 ± 0.19	>3.75	0.47 ± 0.00	0.47 ± 0.00
	MBC	1.88 ± 0.00	0.94 ± 0.00	0.94 ± 0.00	>3.75	0.94 ± 0.00	0.94 ± 0.00
**3a**	MIC	0.94 ± 0.00	0.47 ± 0.00	0.70 ± 0.19	0.70 ± 0.19	0.47 ± 0.00	0.70 ± 0.19
	MBC	3.75 ± 0.00	1.88 ± 0.00	1.88 ± 0.00	0.94 ± 0.00	0.94 ± 0.00	1.88 ± 0.00
**3b**	MIC	1.41 ± 0.38	0.70 ± 0.19	0.70 ± 0.19	>3.75	0.70 ± 0.19	0.70 ± 0.19
	MBC	1.88 ± 0.00	0.94 ± 0.00	0.94 ± 0.00	>3.75	0.94 ± 0.00	0.94 ± 0.00
**5**	MIC	1.41 ± 0.38	0.47 ± 0.00	0.70 ± 0.19	0.35 ± 0.08	0.70 ± 0.19	1.41 ± 0.38
	MBC	1.88 ± 0.00	0.94 ± 0.00	0.94 ± 0.00	0.47 ± 0.00	0.94 ± 0.00	1.88 ± 0.00
**6**	MIC	0.94 ± 0.00	0.70 ± 0.19	0.70 ± 0.19	>3.75	0.70 ± 0.19	0.47 ± 0.00
	MBC	1.88 ± 0.00	0.94 ± 0.00	0.94 ± 0.00	>3.75	0.94 ± 0.00	0.94 ± 0.00
**8**	MIC	2.50 ± 0.88	1.41 ± 0.38	1.41 ± 0.00	0.70 ± 0.19	0.70 ± 0.19	0.94 ± 0.00
	MBC	3.75 ± 0.00	1.88 ± 0.00	1.88 ± 0.00	0.94 ± 0.00	0.94 ± 0.00	1.88 ± 0.00
**10**	MIC	1.41 ± 0.38	0.23 ± 0.00	0.70 ± 0.19	0.70 ± 0.19	0.23 ± 0.00	0.70 ± 0.19
	MBC	1.88 ± 0.00	0.47 ± 0.00	0.94 ± 0.00	0.94 ± 0.00	0.47 ± 0.00	0.94 ± 0.00
Streptomycin	MIC	0.10 ± 0.00	0.02 ± 0.00	0.15 ± 0.00	0.10 ± 0.00	0.10 ± 0.00	0.02 ± 0.00
	MBC	0.20 ± 0.01	0.05 ± 0.00	0.30 ± 0.01	0.20 ± 0.00	0.20 ± 0.01	0.05 ± 0.00
Ampicillin	MIC	0.10 ± 0.00	0.10 ± 0.00	0.15 ± 0.00	0.15 ± 0.00	0.10 ± 0.00	0.10 ± 0.00
	MBC	0.15 ± 0.00	0.15 ± 0.00	0.30 ± 0.02	0.20 ± 0.01	0.20 ± 0.00	0.15 ± 0.01

*S.a.*—*Staphylococcus aureus*, *B.c Bacillus cereus*, *l.m.*—*Listeria monocytogenes*, *E.c.*—*Escherichia coli*, *S.t.*—*Salmonella typhimurium*, *En.c.*—*Enterobacter cloacae*, Relative standard deviations were all < 2.0. *Amp.*: *Ampicillin*, *Strept.*: *Streptomycin*. This experiment was performed in duplicate.

**Table 2:** Antifungal activity of heteroaryl derivatives of benzothiazole (mg/mL).

No.		*A.f.*	*A.n.*	*A.v.*	*P.f.*	*T.v.*	*P.v.c.*
**2a**	MIC	0.23 ± 0.00	0.23 ± 0.00	0.11 ± 0.00	0.35 ± 0.08	0.11 ± 0.00	0.11 ± 0.00
	MFC	0.47 ± 0.00	0.47 ± 0.00	0.23 ± 0.00	0.47 ± 0.00	0.23 ± 0.00	0.23 ± 0.00
**2b**	MIC	0.23 ± 0.00	0.23 ± 0.00	0.23 ± 0.00	0.23 ± 0.00	0.11 ± 0.00	0.23 ± 0.00
	MFC	0.47 ± 0.00	0.47 ± 0.00	0.47 ± 0.00	0.47 ± 0.00	0.23 ± 0.00	0.47 ± 0.00
**2c**	MIC	0.23 ± 0.00	0.23 ± 0.00	0.23 ± 0.00	0.35 ± 0.08	0.23 ± 0.00	0.23 ± 0.00
	MFC	0.47 ± 0.00	0.47 ± 0.00	0.47 ± 0.00	0.47 ± 0.00	0.47 ± 0.00	0.47 ± 0.00
**2d**	MIC	0.17 ± 0.05	0.11 ± 0.00	0.17 ± 0.05	0.17 ± 0.05	0.08 ± 0.00	0.17 ± 0.05
	MFC	0.23 ± 0.00	0.23 ± 0.00	0.23 ± 0.00	0.23 ± 0.00	0.11 ± 0.00	0.23 ± 0.00
**2e**	MIC	0.23 ± 0.00	0.23 ± 0.00	0.35 ± 0.08	0.35 ± 0.08	0.17 ± 0.05	0.35 ± 0.08
	MFC	0.47 ± 0.00	0.47 ± 0.00	0.47 ± 0.00	0.47 ± 0.00	0.23 ± 0.00	0.47 ± 0.00
**2f**	MIC	0.47 ± 0.00	0.23 ± 0.00	0.23 ± 0.00	0.23 ± 0.00	0.17 ± 0.05	0.23 ± 0.00
	MFC	0.94 ± 0.00	0.47 ± 0.00	0.47 ± 0.00	0.47 ± 0.00	0.23 ± 0.00	0.47 ± 0.00
**2g**	MIC	0.47 ± 0.00	0.17 ± 0.05	0.23 ± 0.00	0.35 ± 0.08	0.17 ± 0.05	0.35 ± 0.08
	MFC	0.94 ± 0.00	0.23 ± 0.00	0.47 ± 0.00	0.47 ± 0.00	0.23 ± 0.00	0.47 ± 0.00
**2h**	MIC	0.47 ± 0.00	0.23 ± 0.00	0.35 ± 0.08	0.35 ± 0.08	0.11 ± 0.00	0.70 ± 0.19
	MFC	0.94 ± 0.00	0.47 ± 0.00	0.47 ± 0.00	0.47 ± 0.00	0.23 ± 0.00	0.94 ± 0.00
**2i**	MIC	0.23 ± 0.00	0.11 ± 0.00	0.23 ± 0.00	0.23 ± 0.00	0.06 ± 0.00	0.23 ± 0.00
	MFC	0.47 ± 0.00	0.23 ± 0.00	0.47 ± 0.00	0.47 ± 0.00	0.11 ± 0.00	0.47 ± 0.00
**2j**	MIC	0.23 ± 0.00	0.23 ± 0.00	0.11 ± 0.00	0.23 ± 0.00	0.11 ± 0.00	0.23 ± 0.00
	MFC	0.47 ± 0.00	0.47 ± 0.00	0.23 ± 0.00	0.47 ± 0.00	0.23 ± 0.00	0.47 ± 0.00
**2k**	MIC	0.23 ± 0.00	0.47 ± 0.00	0.47 ± 0.00	0.47 ± 0.00	0.23 ± 0.00	0.47 ± 0.00
	MFC	0.47 ± 0.00	0.94 ± 0.00	0.94 ± 0.00	0.94 ± 0.00	0.47 ± 0.00	0.94 ± 0.00
**2l**	MIC	0.23 ± 0.00	0.17 ± 0.05	0.23 ± 0.00	0.23 ± 0.00	0.11 ± 0.00	0.23 ± 0.00
	MFC	0.47 ± 0.00	0.23 ± 0.00	0.47 ± 0.00	0.47 ± 0.00	0.23 ± 0.00	0.47 ± 0.00
**2m**	MIC	0.17 ± 0.05	0.11 ± 0.00	0.11 ± 0.00	0.23 ± 0.00	0.17 ± 0.05	0.35 ± 0.08
	MFC	0.23 ± 0.00	0.23 ± 0.00	0.23 ± 0.00	0.47 ± 0.00	0.23 ± 0.00	0.47 ± 0.00
**2n**	MIC	0.47 ± 0.00	0.23 ± 0.00	0.23 ± 0.00	0.35 ± 0.08	0.23 ± 0.00	0.35 ± 0.08
	MFC	0.94 ± 0.00	0.47 ± 0.00	0.47 ± 0.00	0.47 ± 0.00	0.47 ± 0.00	0.47 ± 0.00
**2o**	MIC	0.17 ± 0.05	0.17 ± 0.05	0.11 ± 0.00	0.23 ± 0.00	0.17 ± 0.05	0.23 ± 0.00
	MFC	0.23 ± 0.00	0.23 ± 0.00	0.23 ± 0.00	0.47 ± 0.00	0.23 ± 0.00	0.47 ± 0.00
**3a**	MIC	0.17 ± 0.05	0.11 ± 0.00	0.23 ± 0.00	0.17 ± 0.05	0.11 ± 0.00	0.23 ± 0.00
	MFC	0.23 ± 0.00	0.23 ± 0.00	0.47 ± 0.00	0.23 ± 0.00	0.23 ± 0.00	0.47 ± 0.00
**3b**	MIC	0.23 ± 0.00	0.23 ± 0.00	0.17 ± 0.05	0.17 ± 0.05	0.06 ± 0.00	0.17 ± 0.05
	MFC	0.47 ± 0.00	0.47 ± 0.00	0.23 ± 0.00	0.23 ± 0.00	0.11 ± 0.00	0.23 ± 0.00
**5**	MIC	0.23 ± 0.00	0.35 ± 0.08	0.23 ± 0.00	0.23 ± 0.00	0.11 ± 0.00	0.23 ± 0.00
	MFC	0.47 ± 0.00	0.47 ± 0.00	0.47 ± 0.00	0.47 ± 0.00	0.23 ± 0.00	0.47 ± 0.00
**6**	MIC	0.23 ± 0.00	0.35 ± 0.08	0.23 ± 0.00	0.23 ± 0.00	0.06 ± 0.00	0.23 ± 0.00
	MFC	0.47 ± 0.00	0.47 ± 0.00	0.47 ± 0.00	0.47 ± 0.00	0.11 ± 0.00	0.47 ± 0.00
**8**	MIC	3.75 ± 0.00	3.75 ± 0.00	3.75 ± 0.00	3.75 ± 0.00	3.75 ± 0.00	3.75 ± 0.00
	MFC	>3.75	>3.75	>3.75	>3.75	>3.75	>3.75
**10**	MIC	1.88 ± 0.00	0.47 ± 0.00	0.70 ± 0.019	1.41 ± 0.38	0.70 ± 0.19	2.50 ± 0.88
	MFC	3.75 ± 0.00	0.94 ± 0.00	0.94 ± 0.00	1.88 ± 0.00	0.94 ± 0.00	3.75 ± 0.00
Bifonazole	MIC	0.15 ± 0.00	0.15 ± 0.00	0.10 ± 0.00	0.20 ± 0.00	0.15 ± 0.00	0.10 ± 0.00
	MFC	0.20 ± 0.00	0.20 ± 0.00	0.20 ± 0.00	0.25 ± 0.00	0.20 ± 0.00	0.20 ± 0.00
Ketoconazole	MIC	0.20 ± 0.00	0.20 ± 0.00	0.20 ± 0.00	0.20 ± 0.00	1.00 ± 0.01	0.20 ± 0.00
	MFC	0.50 ± 0.00	0.50 ± 0.00	0.50 ± 0.00	0.50 ± 0.00	1.50 ± 0.00	0.30 ± 0.010

*A.f.*—*A. fumigatus*, *A.n.*—*A. niger*, *A.v.*—*A. versicolor*, *P.f.*—*P. funiculosum*, *T.v.*—*T. viride*, *P.v.c.*—*P. cyclopium var. verucosum*. Relative standard deviations were all < 2.20. This experiment was performed in duplicate.

**Table 3:** Molecular docking free binding energies (kcal/mol) to antibacterial targets.

Comp.	Est. Binding Energy (kcal/mol)					I-H *E. coli* MurB	Residues *E. coli* MurB
	*E. coli* Gyrase 1KZN	Thymidylate Kinase 4QGG	*E. coli* Primase 1DDE	*E. coli* MurA JV4T	*E. coli* MurB 2Q85		
**2a**	−4.52	-	-	−3.62	−8.03	1	Ser229
**2b**	−5.28	-	−1.23	−4.27	−7.86	1	Arg158
**2c**	−4.39	−2.55	-	−5.19	−10.13	2	Ser50, Ser229
**2d**	−5.19	−1.03	−2.26	−6.52	−9.64	2	Arg158, Arg213
**2e**	−4.55	-	−1.39	−5.24	−9.61	2	Arg213, Ser229
**2f**	−4.37	-	-	−5.37	−6.53	1	Arg213
**2g**	−5.37	−1.54	−2.33	−6.72	−10.02	2	Arg158, Arg213
**2h**	−4.19	-	-	−5.68	−9.71	2	Arg213, Ser229
**2i**	−5.63	−1.28	−1.30	−5.22	−9.34	2	Ser50, Ser229
**2j**	−5.27	-	-	−6.34	−10.75	3	Ser50, Ser116, Ile173
**2k**	−4.23	-	-	−4.53	−8.76	1	Ser229
**2l**	−4.96	-	−2.38	−6.59	−9.11	2	Ser50, Ser229
**2m**	−5.23	-	-	−4.56	−7.80	1	Arg158
**2n**	−4.31	−1.85	-	−3.11	−7.30	1	Arg213
**2o**	−3.62	-	-	−2.54	−7.35	1	Arg213
**3a**	−4.35	-	-	−3.64	−8.45	1	Ser229
**3b**	−2.32	−1.68	-	−4.52	−7.18	1	Arg158
**5**	−5.12	-	−1.30	−5.57	−9.53	2	Arg213, Ser229
**6**	−3.66	-	-	−2.50	−7.42	1	Arg213
**8**	−5.10	−1.52	−2.46	−6.22	−9.90	2	Arg158, Arg213
**10**	−4.28	-	-	−3.55	−6.92	1	Arg213
Naphthyl Tetronic Acid inhibitor	-	-	-	-	−8.82	-	Asn233

**Table 4:** Molecular docking free binding energies (kcal/mol) to antifungal targets.

	Est. Binding Energy(kcal/mol)		Residues Involved in H-Bond Formation	Residues Involved in Hydrophobic Interactions	Residues Involved in Aromatic Interactions	Interactions with HEM601
N/N	DNA TopoIV 1S16	CYP51 of *C. albicans* 5V5Z				
**2a**	−1.38	−9.85	Tyr132	Tyr118, Leu121, Thr311, Phe380, Met508, Hem601	Hem601	Hydrophobic, aromatic
**2b**	−3.59	−8.82	Tyr132	Tyr118, Tyr122, Ile304, Thr311, Hem601	Tyr118	Hydrophobic
**2c**	−2.64	−8.03	-	Tyr118, Thr311, Leu376, Met508, Hem601	Hem601	Hydrophobic, aromatic
**2d**	−3.57	−11.32	-	Tyr118, Leu121, Tyr122, Thr311, Leu376, Phe380, Met508, Hem601	Tyr118, Hem601	Hydrophobic, aromatic
**2e**	−3.15	−8.50	Tyr118	Tyr118, Leu376, Met508, Hem601	Tyr118	Hydrophobic
**2f**	−1.29	−7.46	-	Met508, Hem601	Hem601	Hydrophobic, aromatic
**2g**	-	−7.93	-	Ile304, Thr311, Met508, Hem601	-	Hydrophobic
**2h**	−2.44	−7.21	-	Tyr118, Leu376, Met508, Hem601	Tyr118	Hydrophobic
**2i**	-	−9.82	Tyr132	Tyr118, Phe380, Met508, Hem601	-	Hydrophobic
**2j**	−3.83	−9.56	-	Tyr118, Tyr122, Thr311, Leu376, Met508, Hem601	Tyr118, Hem601	Hydrophobic, aromatic
**2k**	-	−7.39	-	Tyr118, Met508, Hem601	-	Hydrophobic
**2l**	−1.27	9.52	-	Tyr118, Tyr122, Leu376, Met508, Hem601	Tyr122, Hem601	Hydrophobic, aromatic
**2m**	−3.21	−10.02	Tyr64	Tyr118, Tyr122, Thr311, Leu376, Phe380, Hem601	Tyr118	Hydrophobic
**2n**	-	−7.25	-	Tyr118, Leu376, Met508, Hem601	-	Hydrophobic
**2o**	−2.50	−10.25	-	Tyr118, Leu121, Tyr122, Thr311, Hem601	Hem601	Hydrophobic, aromatic
**3a**	−2.75	−10.31	-	Tyr118, Leu121, Leu376, Phe380, Met508, Hem601	Hem601	Hydrophobic, aromatic
**3b**	−3.23	−10.87	-	Tyr118, Tyr122, Thr311, Leu376, Met508, Hem601	Tyr118, Hem601	Hydrophobic, aromatic
**5**	-	−8.62	-	Tyr118, Tyr122, Thr311, Met508, Hem601	Hem601	Hydrophobic, aromatic
**6**	−2.45	−9.10	-	Tyr118, Tyr122, Ile304, Thr311, Leu376, Hem601	Ile131, Hem601	Hydrophobic, aromatic
**8**	−2.06	−8.21	-	Tyr118, Tyr122, Ile131, Leu376, Met508, Hem601	-	Hydrophobic
**10**	−2.41	−7.20	-	Tyr118, Leu376, Met508, Hem601	Tyr118	Hydrophobic
ketoconazole	-	−8.23	Tyr64	Tyr118, Ile131, Tyr132, Leu300, Ile304, Leu376, Met508, Hem601	Hem601	Hydrophobic, aromatic

**Table 5:** Drug likeness predictions of tested compounds.

No.	MW	Number of HBA ^a^	Number of HBD ^b^	Log *P*_o/w_ (iLOGP) ^c^	Log S ^d^	TPSA ^e^	BBB Permeant ^f^	Lipinski, Ghose, Veber, Egan, and Muegge Violations	Bioavailability Score	Drug-Likeness Model Score
**2a**	311.42	2	0	2.81	Poorly soluble	84.25	No	0	0.55	−0.08
**2b**	309.41	3	0	3.19	Poorly soluble	92.21	No	0	0.55	−0.19
**2c**	371.48	3	0	3.69	Poorly soluble	92.21	No	0	0.55	−0.35
**2d**	385.50	3	0	3.86	Poorly soluble	92.21	No	0	0.55	−0.12
**2e**	405.92	3	0	4.03	Poorly soluble	92.21	No	0	0.55	0.20
**2f**	470.61	4	0	4.30	Poorly soluble	112.52	No	2 *	0.55	0.65
**2g**	492.64	6	0	4.13	Poorly soluble	137.97	No	3 **	0.55	−0.22
**2h**	587.71	5	0	4.65	Insoluble	134.52	No	2 ***	0.17	0.37
**2i**	429.56	3	0	3.65	Poorly soluble	101.32	No	0	0.55	0.06
**2j**	486.61	4	1	3.73	Poorly soluble	130.42	No	0	0.55	0.43
**2k**	497.63	4	0	4.36	Poorly soluble	115.76	No	0	0.55	0.21
**2l**	511.66	4	0	4.31	Poorly soluble	115.76	No	0	0.55	0.42
**2m**	482.58	5	1	3.61	Poorly soluble	138.61	No	0	0.55	0.32
**2n**	496.61	5	1	4.14	Poorly soluble	138.61	No	0	0.55	0.34
**2o**	531.65	4	1	4.38	Poorly soluble	125.72	No	2 ***	0.17	−0.03
**3a**	464.69	2	0	4.75	Poorly soluble	132.86	No	0	0.55	−0.05
**3b**	496.69	4	0	4.87	Poorly soluble	151.32	No	0	0.55	0.43
**5**	501.62	8	0	3.85	Poorly soluble	117.82	No	0	0.55	0.79
**6**	463.61	5	0	3.56	Poorly soluble	99.78	No	0	0.55	0.49
**8**	400.25	4	1	2.93	Poorly soluble	100.94	No	0	0.55	−0.60
**10**	429.29	4	1	2.65	Poorly soluble	105.12	No	0	0.55	0.44

^a^ number of hydrogen bond acceptors; ^b^ number of hydrogen bond donors; ^c^ lipophilicity; ^d^ Water solubility (SILICOS-IT [S = Soluble]); ^e^ topological polar surface area (Å^2^); ^f^ Blood Brain Barrier permeant; * Ghose 2 violations: WLOGP > 5.6, MR > 130, ** Ghose 3 violations: MW > 480, WLOGP > 5.6, MR > 130, *** Lipinski: 2 violations: MW > 500, MLOGP > 4.15.

## Data Availability

Not applicable.

## Supplementary Materials

The following supporting information can be downloaded at: https://www.mdpi.com/article/10.3390/antibiotics11111654/s1, File S1: ^1^H-NMR and ^13^C-NMR of compounds.
