# Supplementary material for: Experimental and In Silico Evaluation of New Heteroaryl Benzothiazole Derivatives as Antimicrobial Agents
Source: Antibiotics (Basel). 2022 Nov 18;11(11):1654. doi: 10.3390/antibiotics11111654 (PMC9686863; doi:10.3390/antibiotics11111654)
Supplement: Supplementary file 1 [file antibiotics-11-01654-s001.zip › antibiotics-2021630-supplementary.pdf]

^{1}H-NMR and ^{13}C-NMR of compounds

Compound 2a

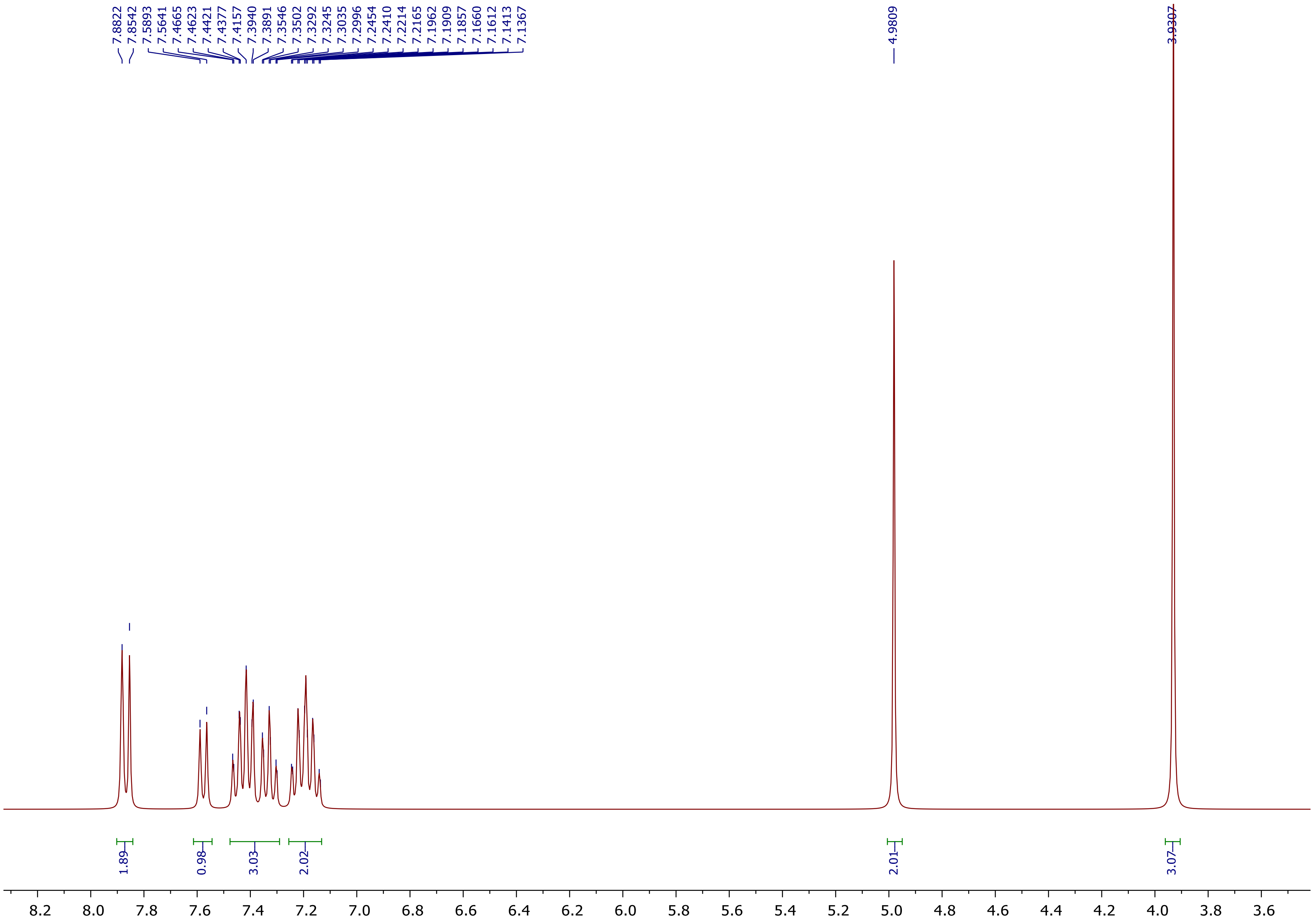

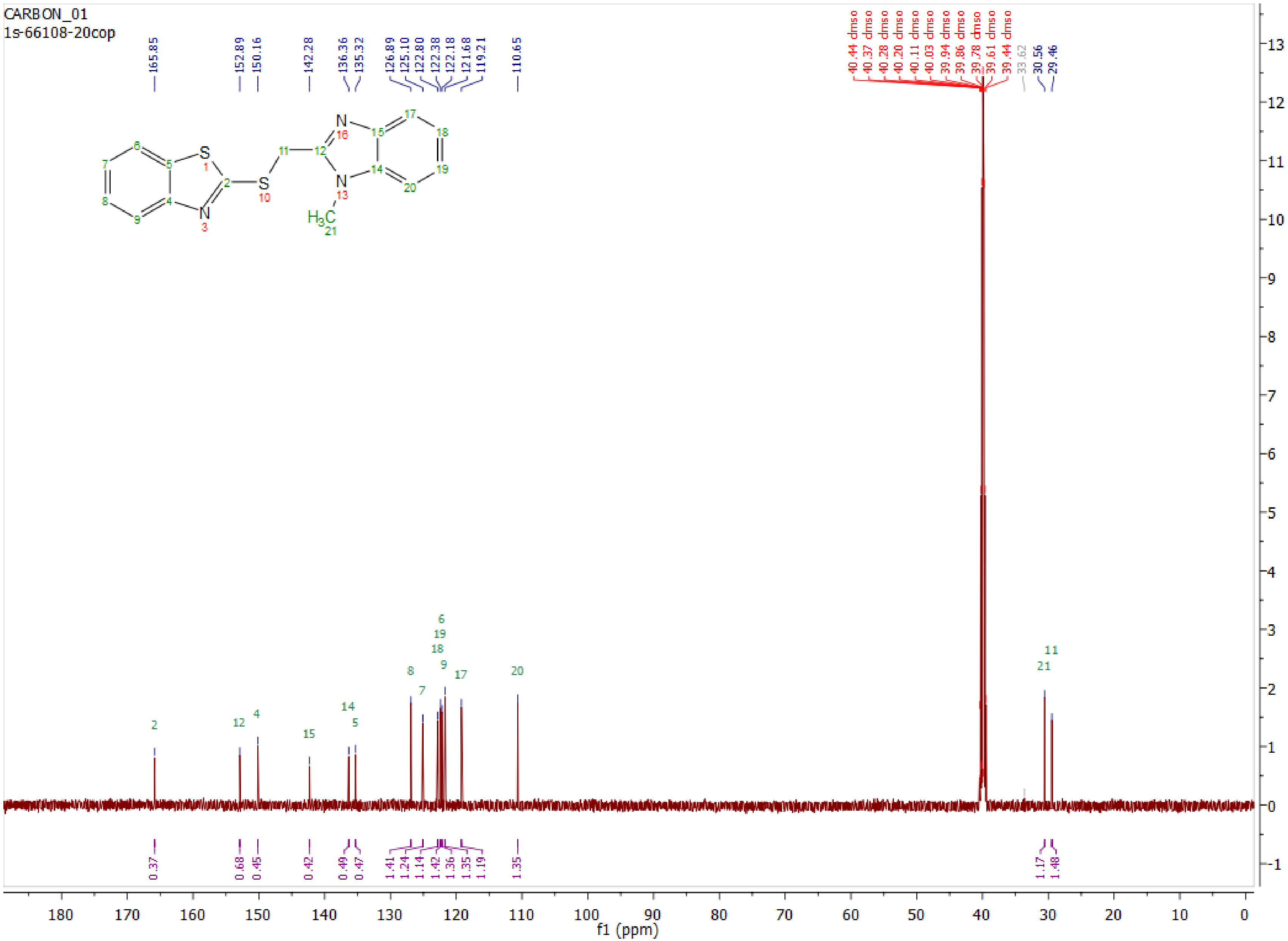
CARBON_01
1s-66108-20cop
165.85
152.89
150.16
142.28
136.36
135.32
126.89
125.10
122.80
122.38
122.18
121.68
119.21
110.65
40.44 dmso
40.37 dmso
40.28 dmso
40.20 dmso
40.11 dmso
40.03 dmso
39.94 dmso
39.86 dmso
39.78 dmso
39.61 dmso
39.44 dmso
33.62
30.56
29.46
H_3C
f1 (ppm)

Compound 2b

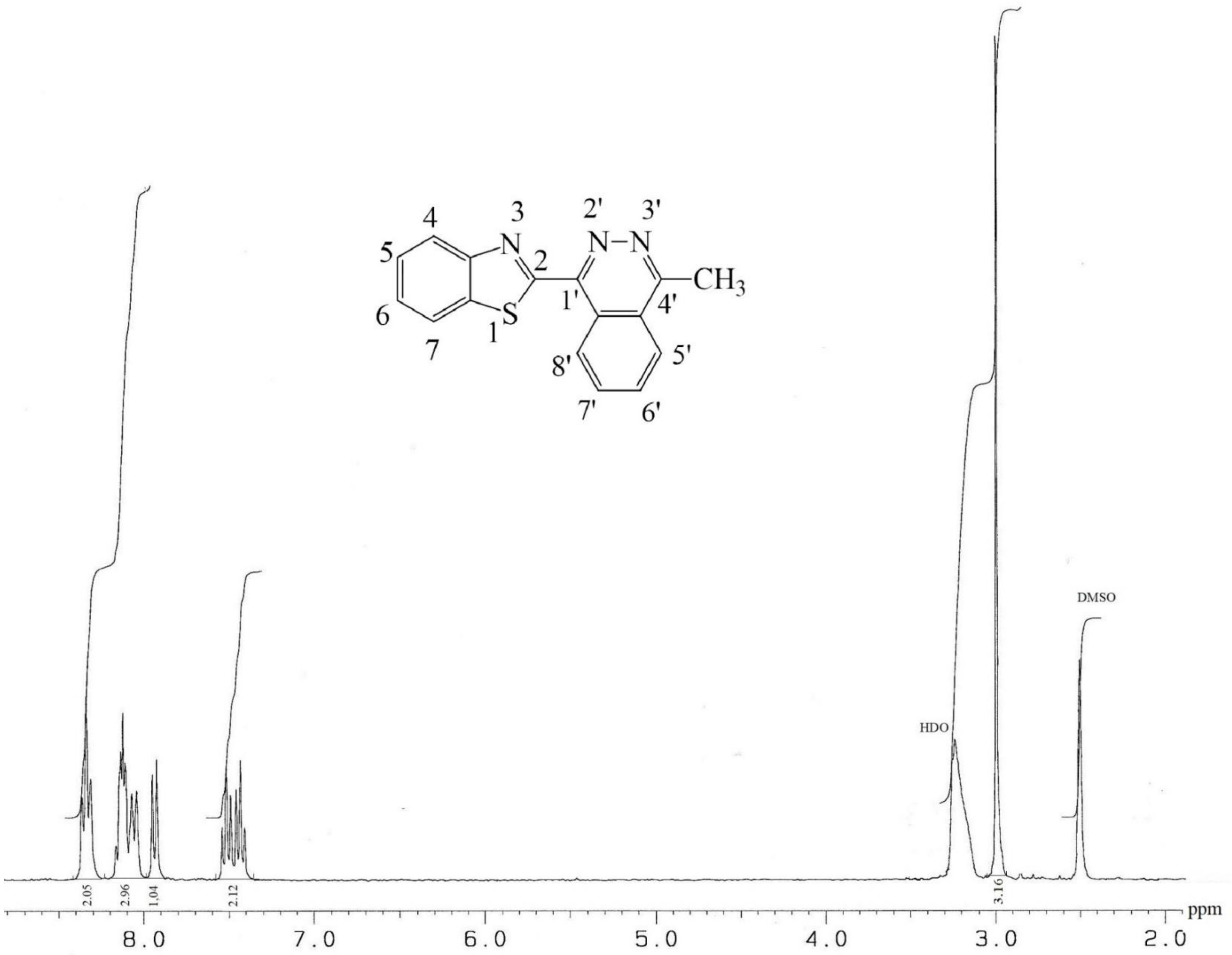

4
3
2'
3'
5
N
N–N
2
CH3
1'
4'
6
S
1
7
8'
5'
7'
6'
DMSO
HDO
2.05
2.96
1.04
2.12
3.16
8.0
7.0
6.0
5.0
4.0
3.0
2.0
ppm

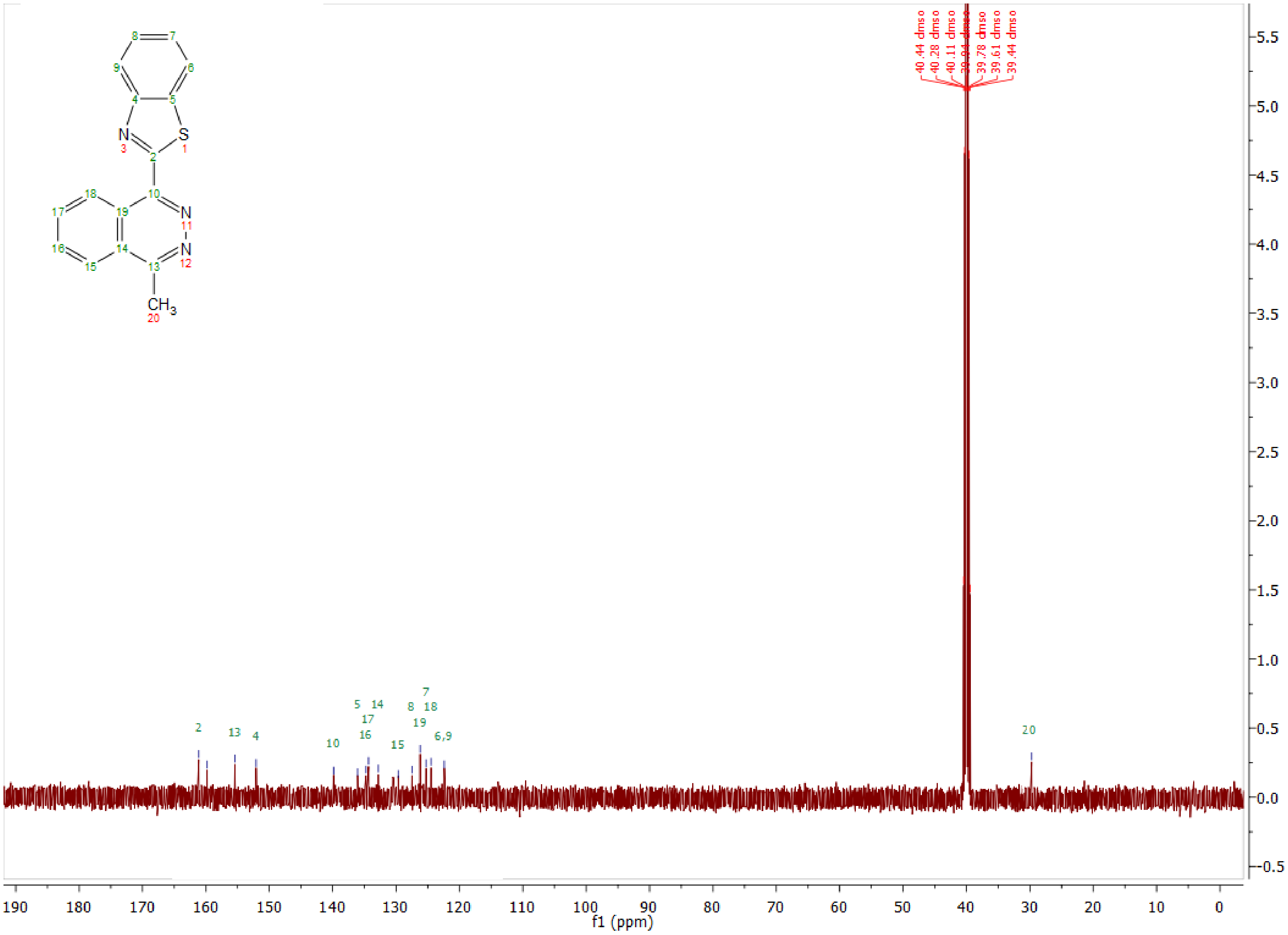
40.44 dmso
40.28 dmso
40.11 dmso
39.78 dmso
39.61 dmso
39.44 dmso
N
S
CH3
2
13
4
10
5
14
17
16
15
8
7
18
19
6,9
20
f1 (ppm)

Compound 2c

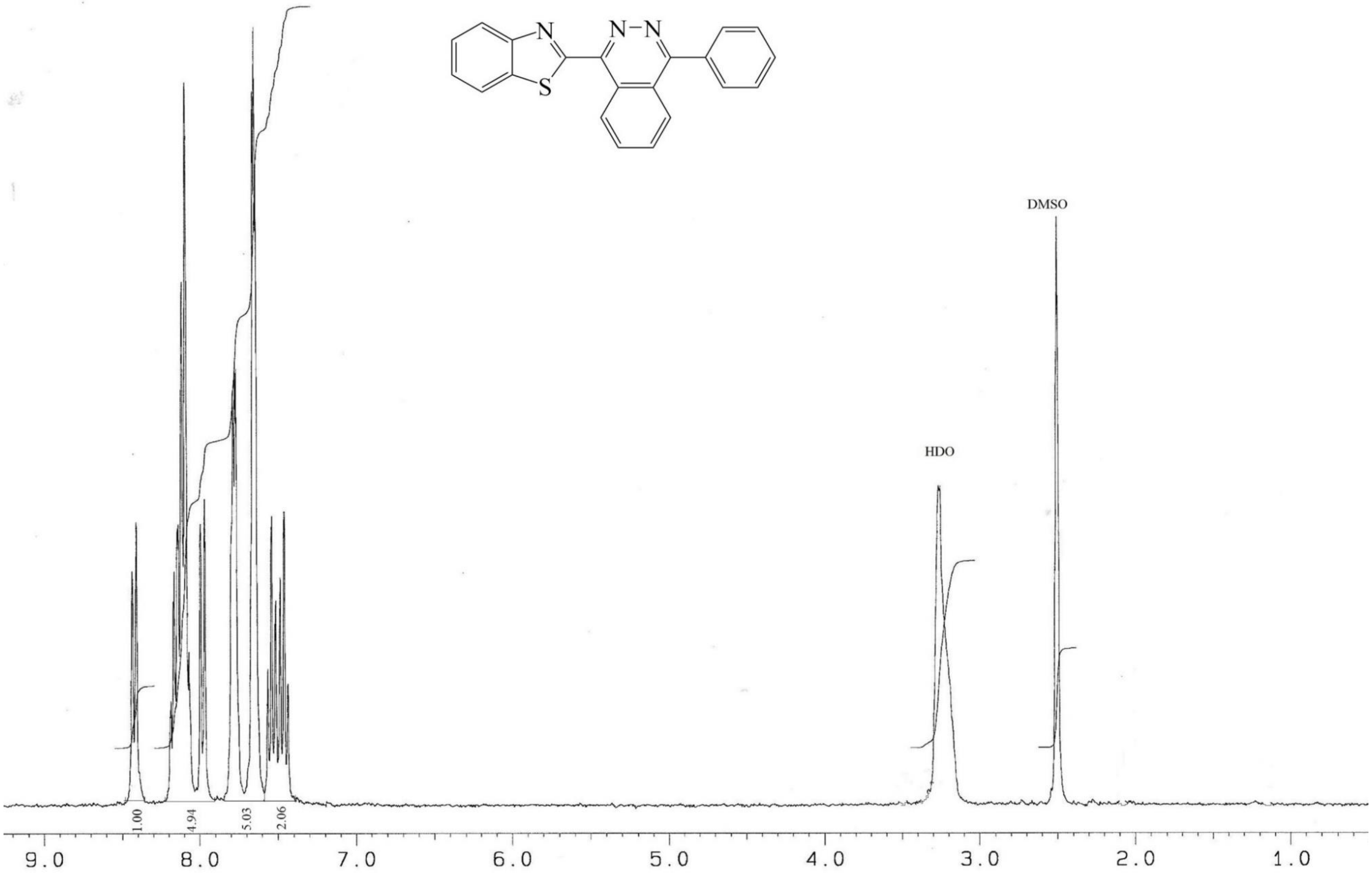

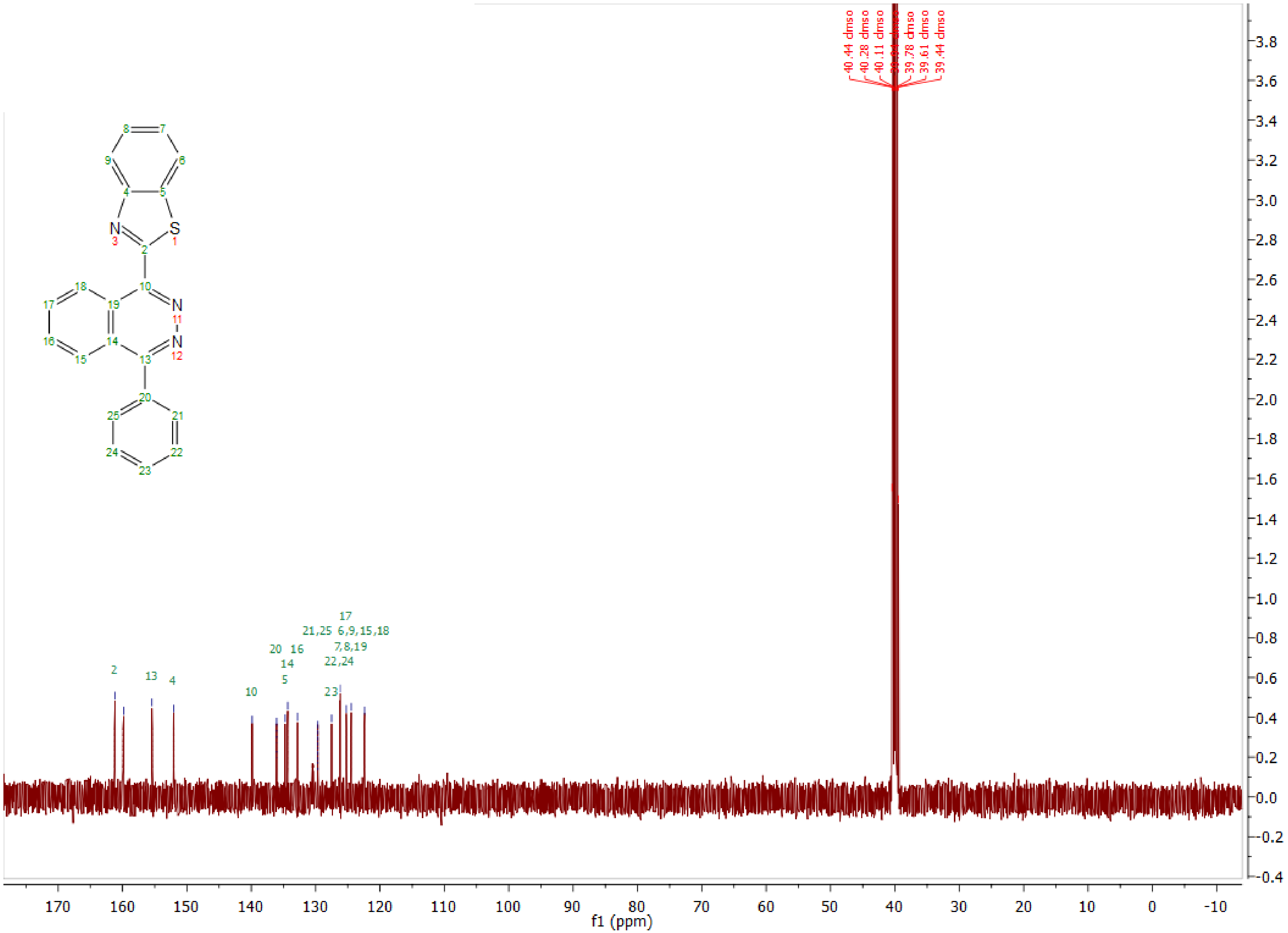

40.44 dmso
40.28 dmso
40.11 dmso
39.78 dmso
39.61 dmso
39.44 dmso
2
13
4
10
5
20
14
16
21,25
17
6,9,15,18
7,8,19
22,24
23
N
S
f1 (ppm)

Compound 2d

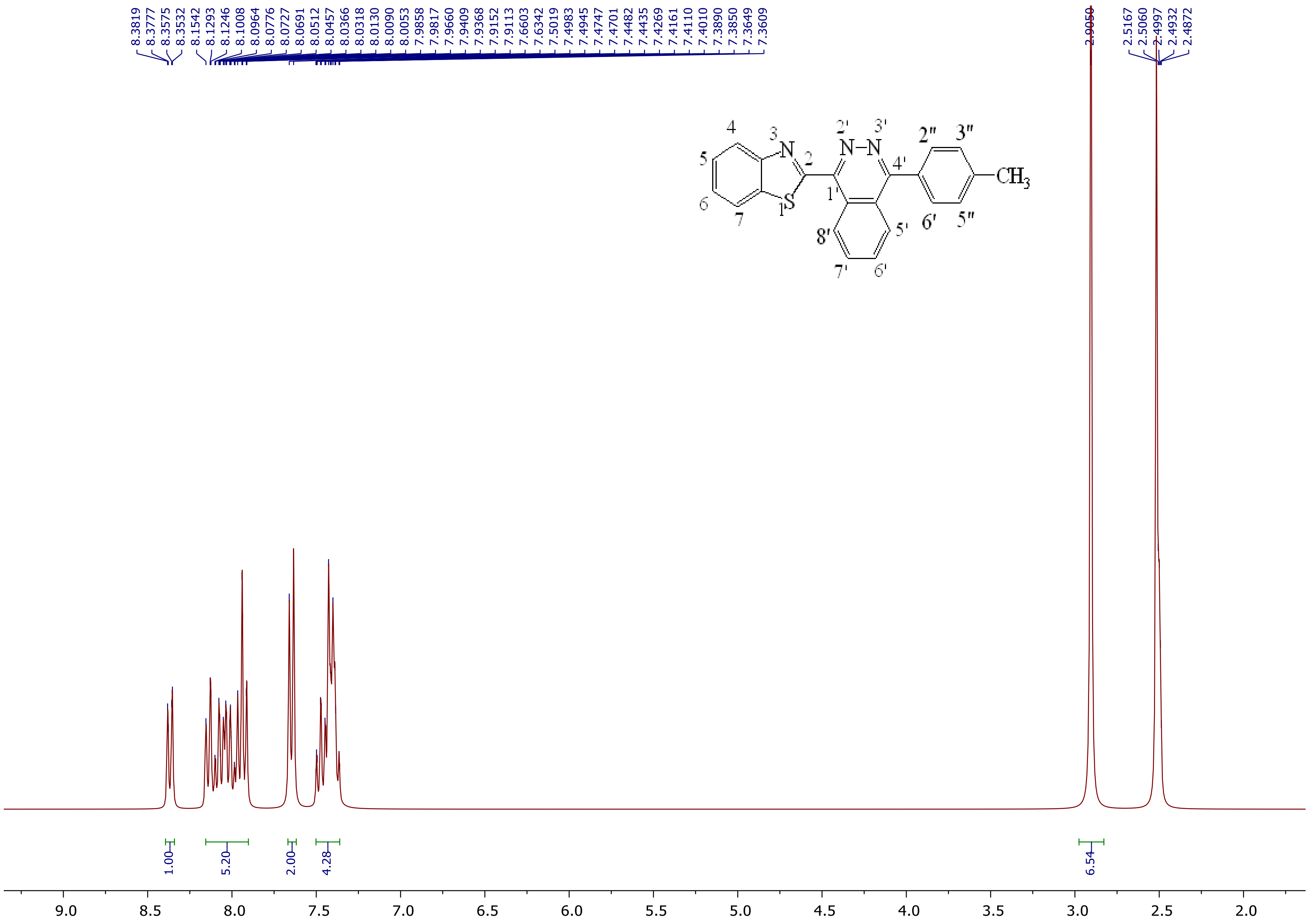
8.3819
8.3777
8.3575
8.3532
8.1542
8.1293
8.1246
8.1008
8.0964
8.0776
8.0727
8.0691
8.0512
8.0457
8.0366
8.0318
8.0130
8.0090
8.0053
7.9858
7.9817
7.9660
7.9409
7.9368
7.9152
7.9113
7.6603
7.6342
7.5019
7.4983
7.4945
7.4747
7.4701
7.4482
7.4435
7.4269
7.4161
7.4110
7.4010
7.3890
7.3850
7.3649
7.3609
2.9050
2.5167
2.5060
2.4997
2.4932
2.4872
1.00
5.20
2.00
4.28
6.54
9.0
8.5
8.0
7.5
7.0
6.5
6.0
5.5
5.0
4.5
4.0
3.5
3.0
2.5
2.0
CH3

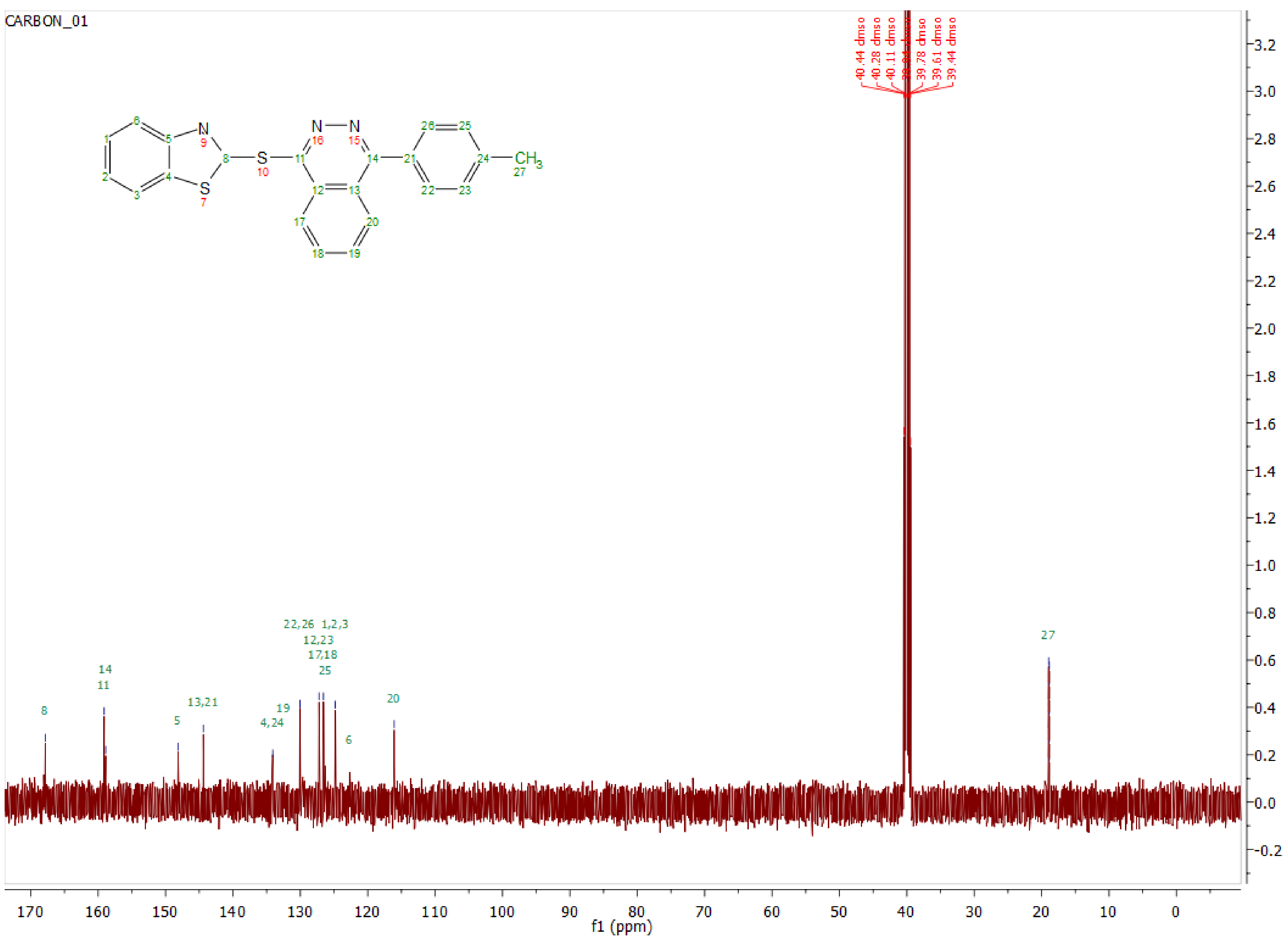

CARBON_01
40.44 dmso
40.28 dmso
40.11 dmso
39.78 dmso
39.61 dmso
39.44 dmso
N
N
S
S
N
N
CH3
8
14
11
5
13,21
19
4,24
22,26
1,2,3
12,23
17,18
25
6
20
27
f1 (ppm)

Compound 2e

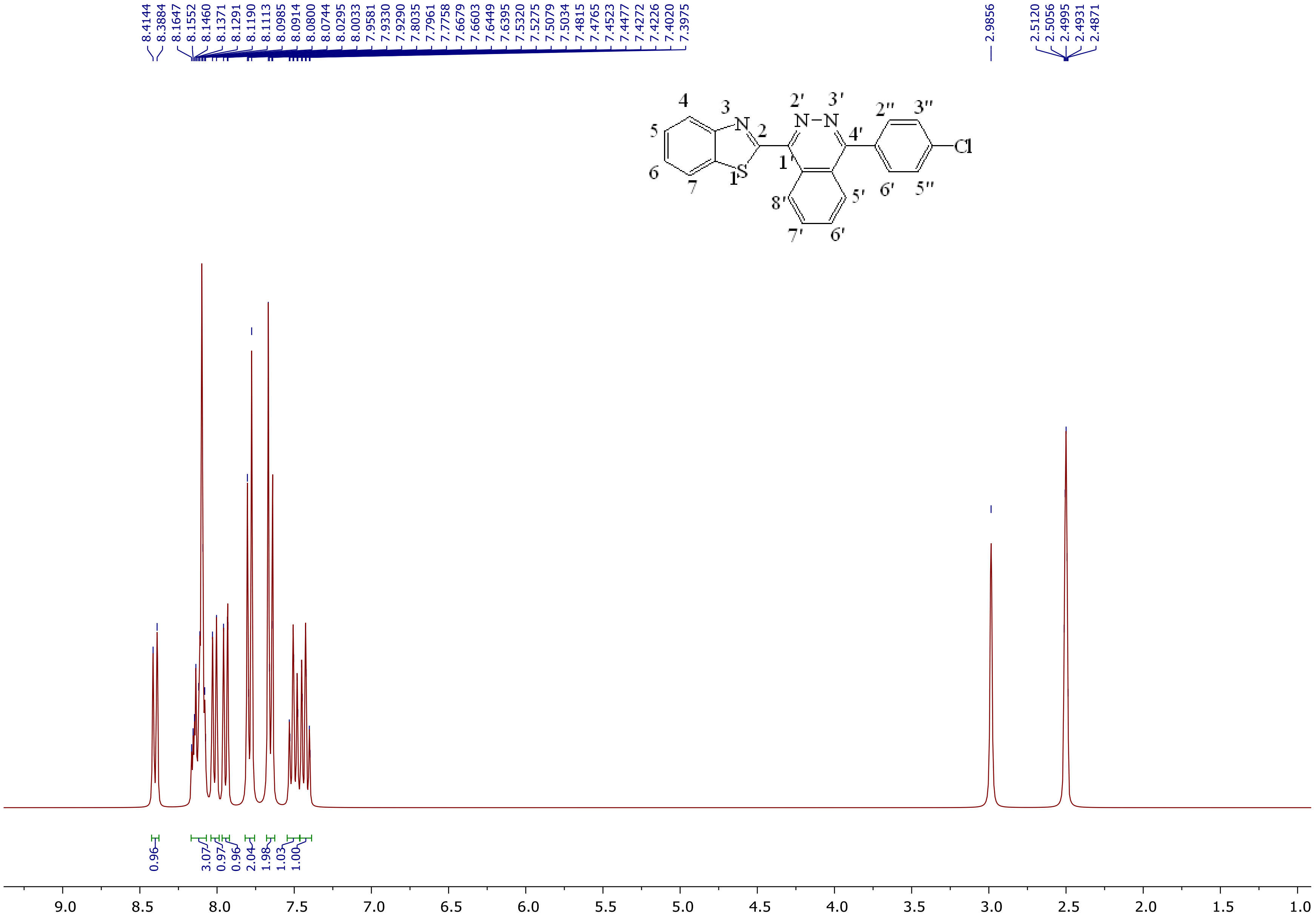

8.4144
8.3884
8.1647
8.1552
8.1460
8.1371
8.1291
8.1190
8.1113
8.0985
8.0914
8.0800
8.0744
8.0295
8.0033
7.9581
7.9330
7.9290
7.8035
7.7961
7.7758
7.6679
7.6603
7.6449
7.6395
7.5320
7.5275
7.5079
7.5034
7.4815
7.4765
7.4523
7.4477
7.4272
7.4226
7.4020
7.3975
2.9856
2.5120
2.5056
2.4995
2.4931
2.4871
0.96
3.07
0.97
0.96
2.04
1.98
1.03
1.00
9.0
8.5
8.0
7.5
7.0
6.5
6.0
5.5
5.0
4.5
4.0
3.5
3.0
2.5
2.0
1.5
1.0

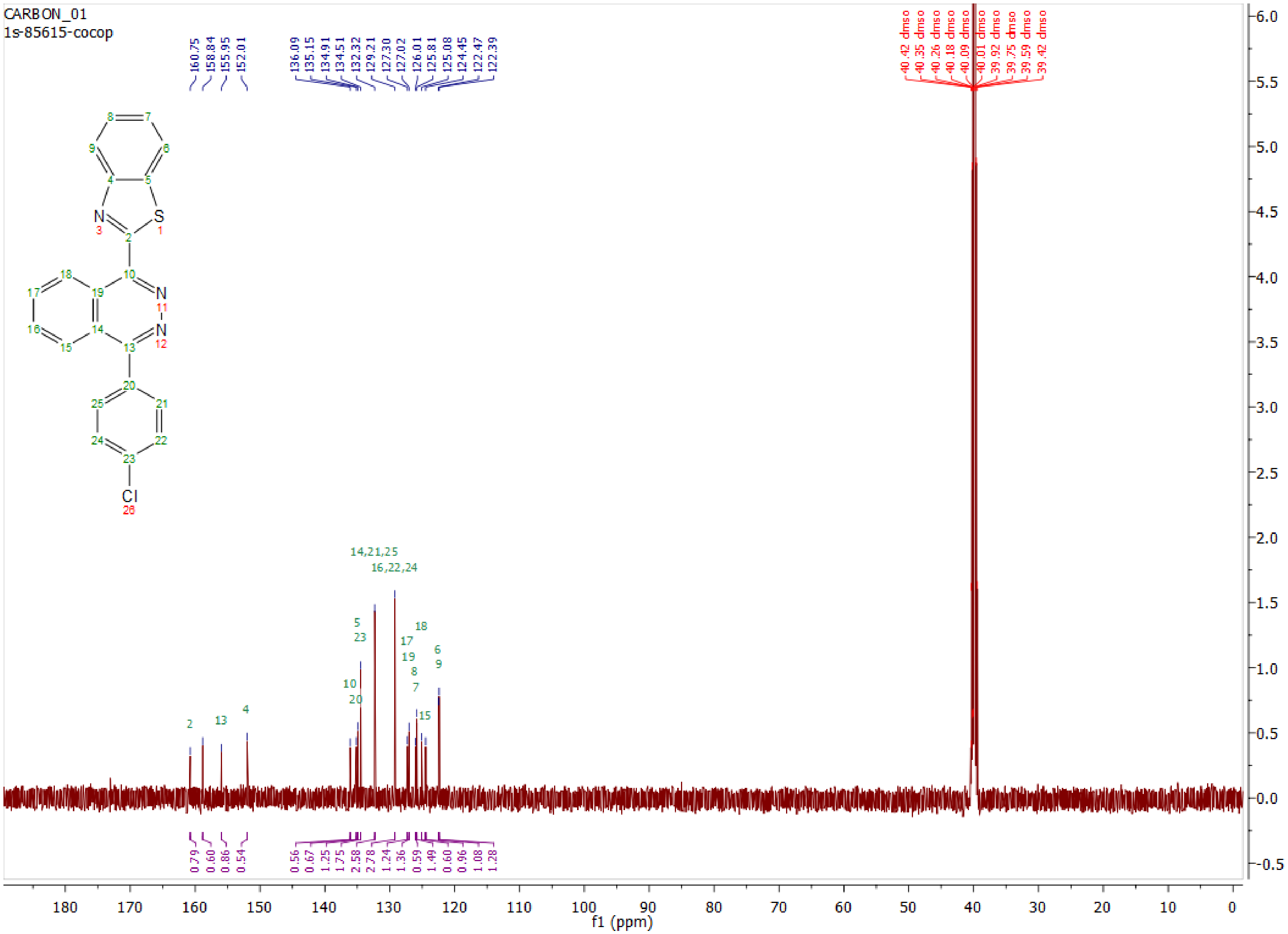

CARBON_01
1s-85615-cocop
160.75
158.84
155.95
152.01
136.09
135.15
134.91
134.51
132.32
129.21
127.30
127.02
126.01
125.81
125.08
124.45
122.47
122.39
40.42 dmso
40.35 dmso
40.26 dmso
40.18 dmso
40.09 dmso
40.01 dmso
39.92 dmso
39.75 dmso
39.59 dmso
39.42 dmso
S
N
N
N
Cl
14,21,25
16,22,24
0.79
0.60
0.86
0.54
0.56
0.67
1.25
1.75
2.58
2.78
1.24
1.36
0.59
1.49
0.60
0.96
1.08
1.28
f1 (ppm)

Compound 2f

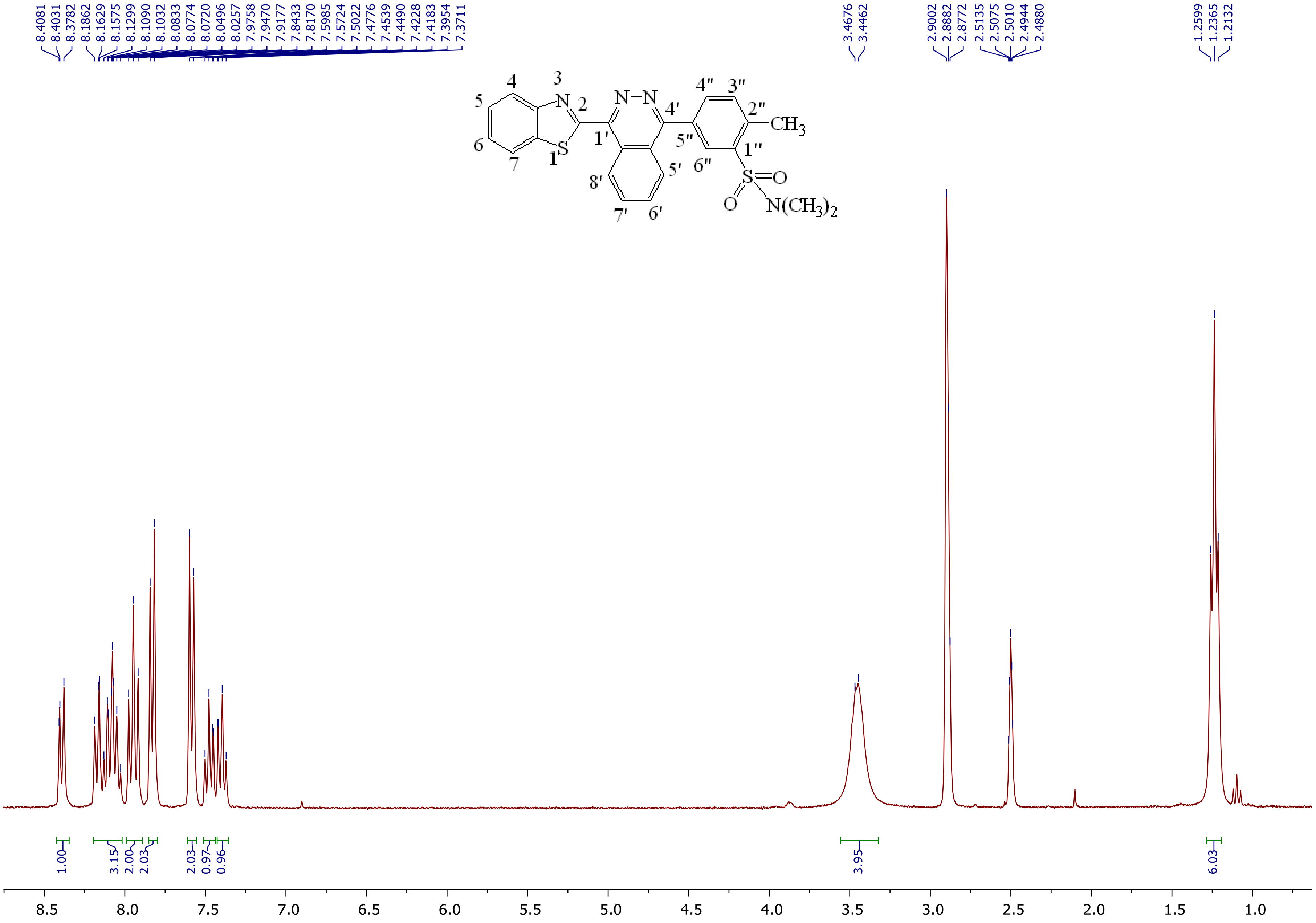

8.4081
8.4031
8.3782
8.1862
8.1629
8.1575
8.1299
8.1090
8.1032
8.0833
8.0774
8.0720
8.0496
8.0257
7.9758
7.9470
7.9177
7.8433
7.8170
7.5985
7.5724
7.5022
7.4776
7.4539
7.4490
7.4228
7.4183
7.3954
7.3711
3.4676
3.4462
2.9002
2.8882
2.8772
2.5135
2.5075
2.5010
2.4944
2.4880
1.2599
1.2365
1.2132
1.00
3.15
2.00
2.03
2.03
0.97
0.96
3.95
6.03
8.5
8.0
7.5
7.0
6.5
6.0
5.5
5.0
4.5
4.0
3.5
3.0
2.5
2.0
1.5
1.0

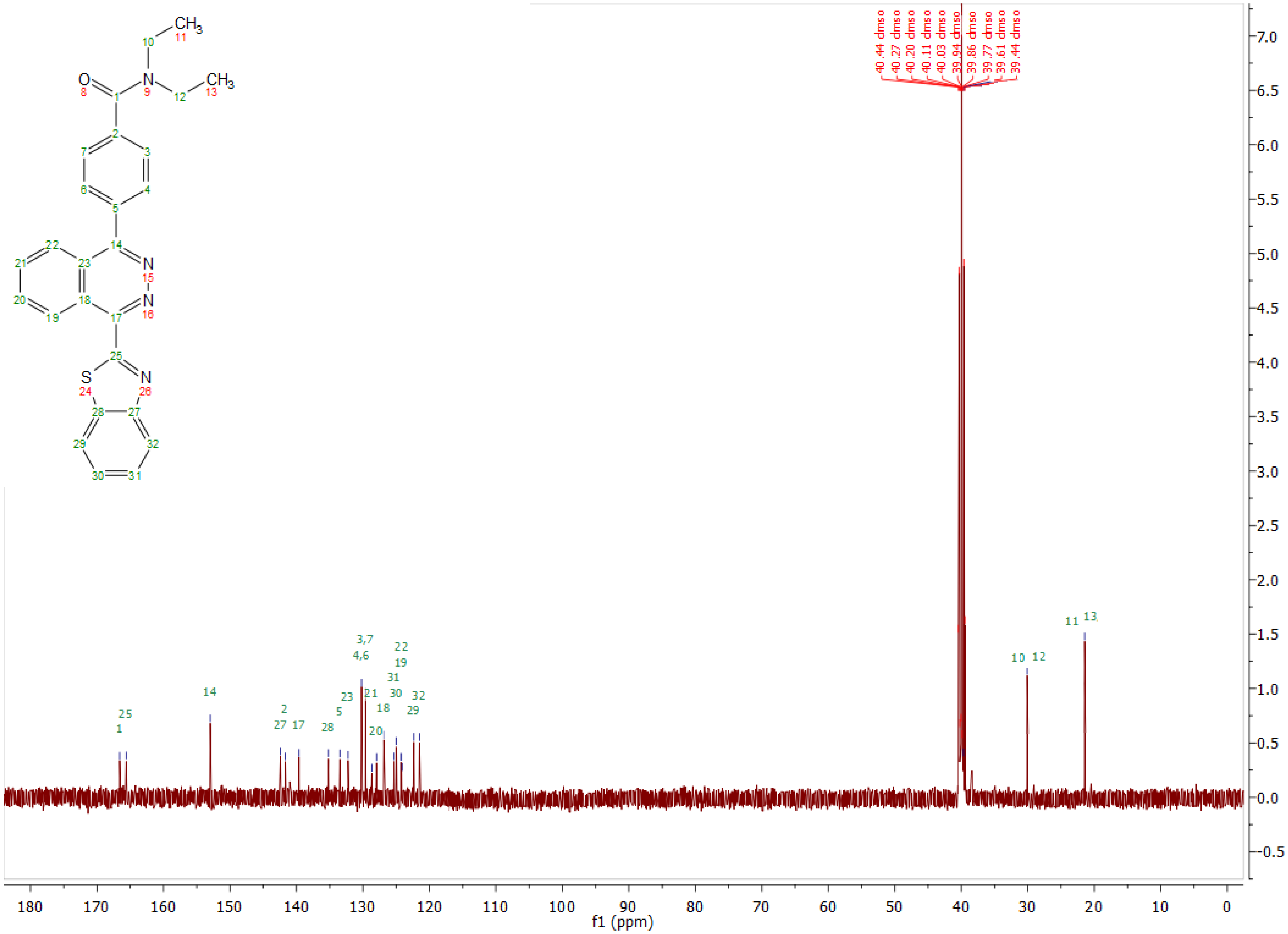

40.44 dmso
40.27 dmso
40.20 dmso
40.11 dmso
40.03 dmso
39.94 dmso
39.86 dmso
39.77 dmso
39.61 dmso
39.44 dmso
f1 (ppm)

Compound 2g

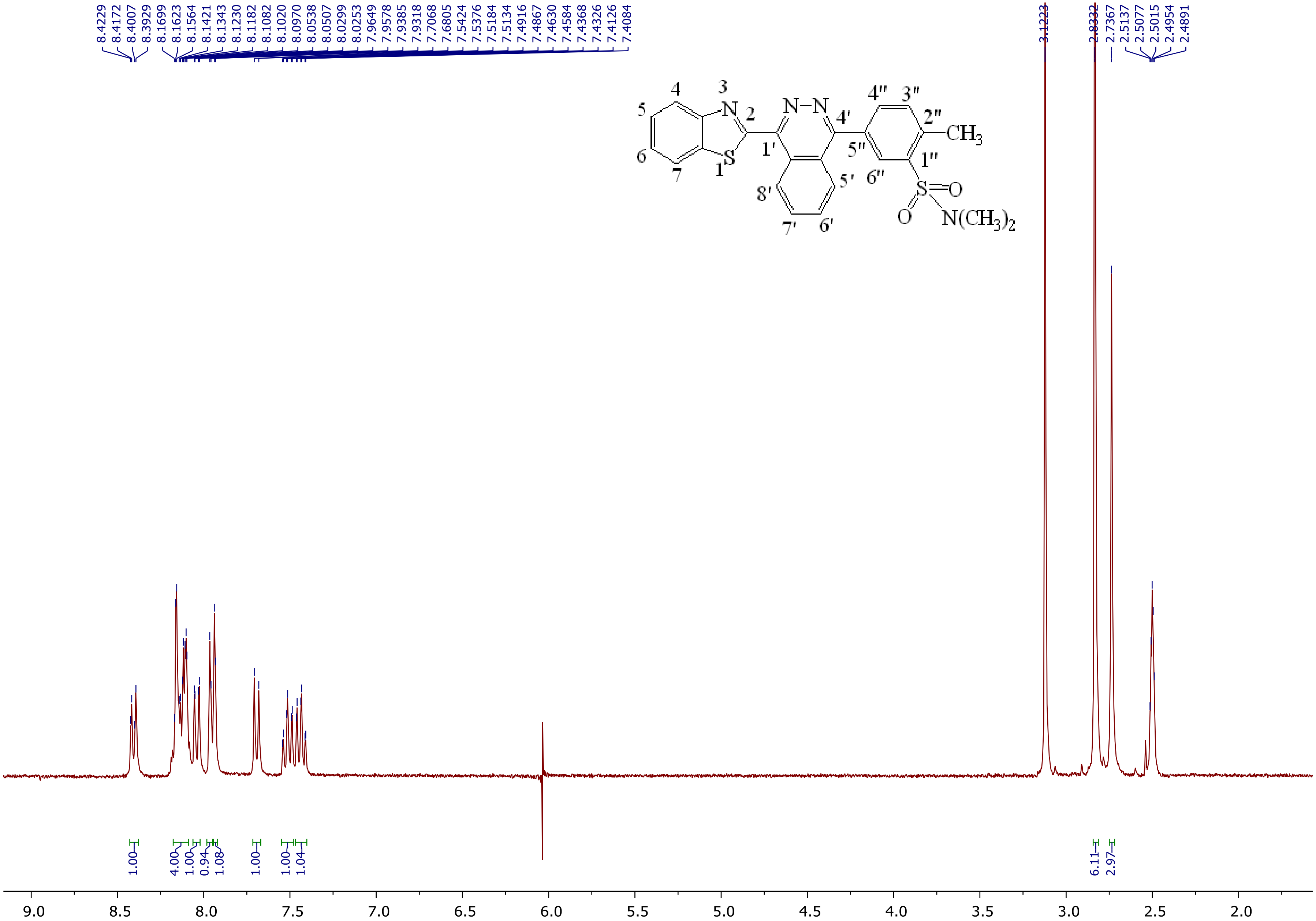

8.4229
8.4172
8.4007
8.3929
8.1699
8.1623
8.1564
8.1421
8.1343
8.1230
8.1182
8.1082
8.1020
8.0970
8.0538
8.0507
8.0299
8.0253
7.9649
7.9578
7.9385
7.9318
7.7068
7.6805
7.5424
7.5376
7.5184
7.5134
7.4916
7.4867
7.4630
7.4584
7.4368
7.4326
7.4126
7.4084
3.1223
2.8332
2.7367
2.5137
2.5077
2.5015
2.4954
2.4891
N–N
CH3
S=O
O
N(CH3)2
1.00
4.00
1.00
0.94
1.08
1.00
1.00
1.04
6.11
2.97
9.0
8.5
8.0
7.5
7.0
6.5
6.0
5.5
5.0
4.5
4.0
3.5
3.0
2.5
2.0

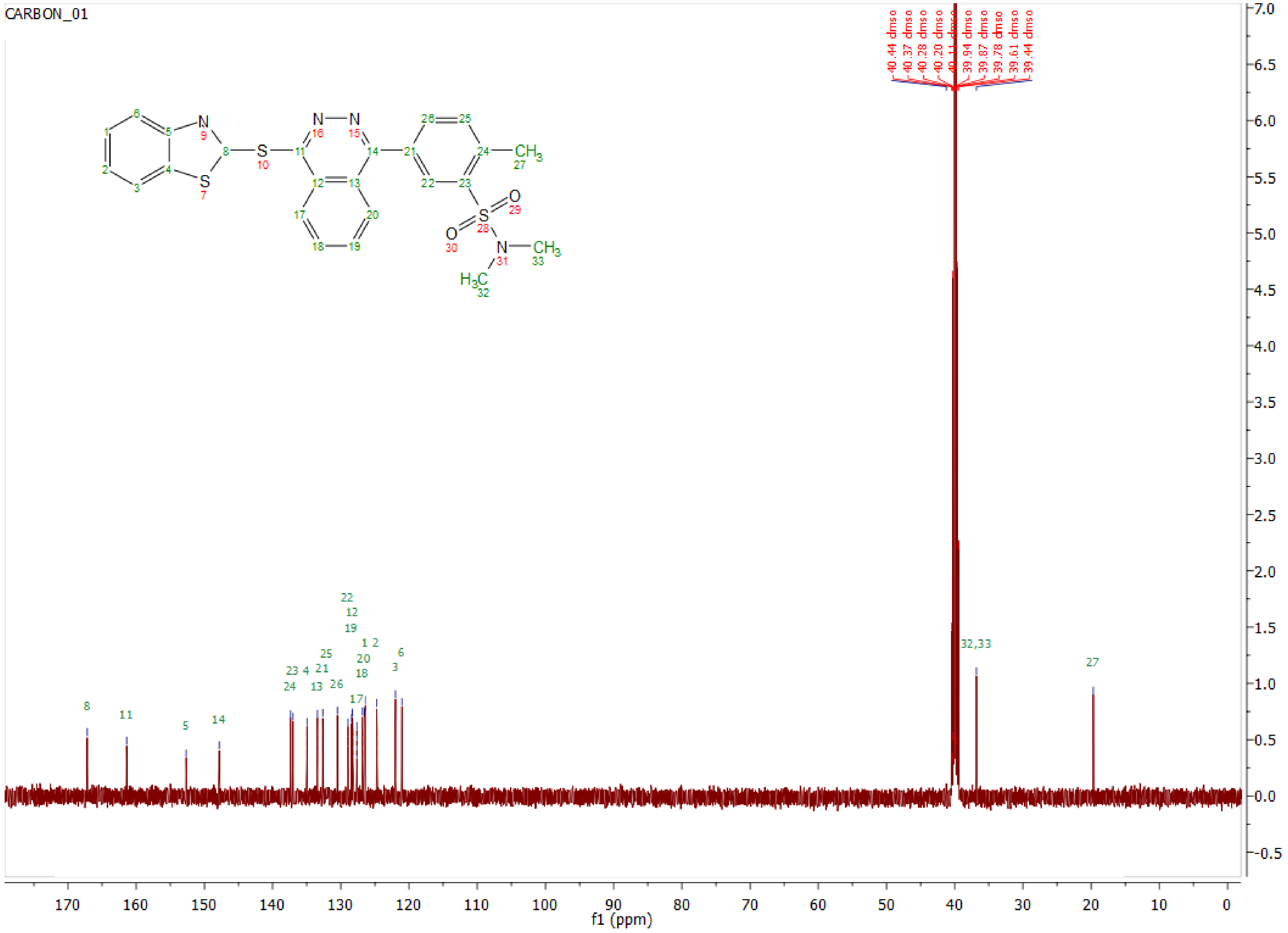
CARBON_01
40.44 dmso
40.37 dmso
40.28 dmso
40.20 dmso
40.11 dmso
39.94 dmso
39.87 dmso
39.78 dmso
39.61 dmso
39.44 dmso
8
11
5
14
23 4
24
13
25
21
26
22
12
19
1 2
20
18
17
6
3
32,33
27
f1 (ppm)

Compound 2h

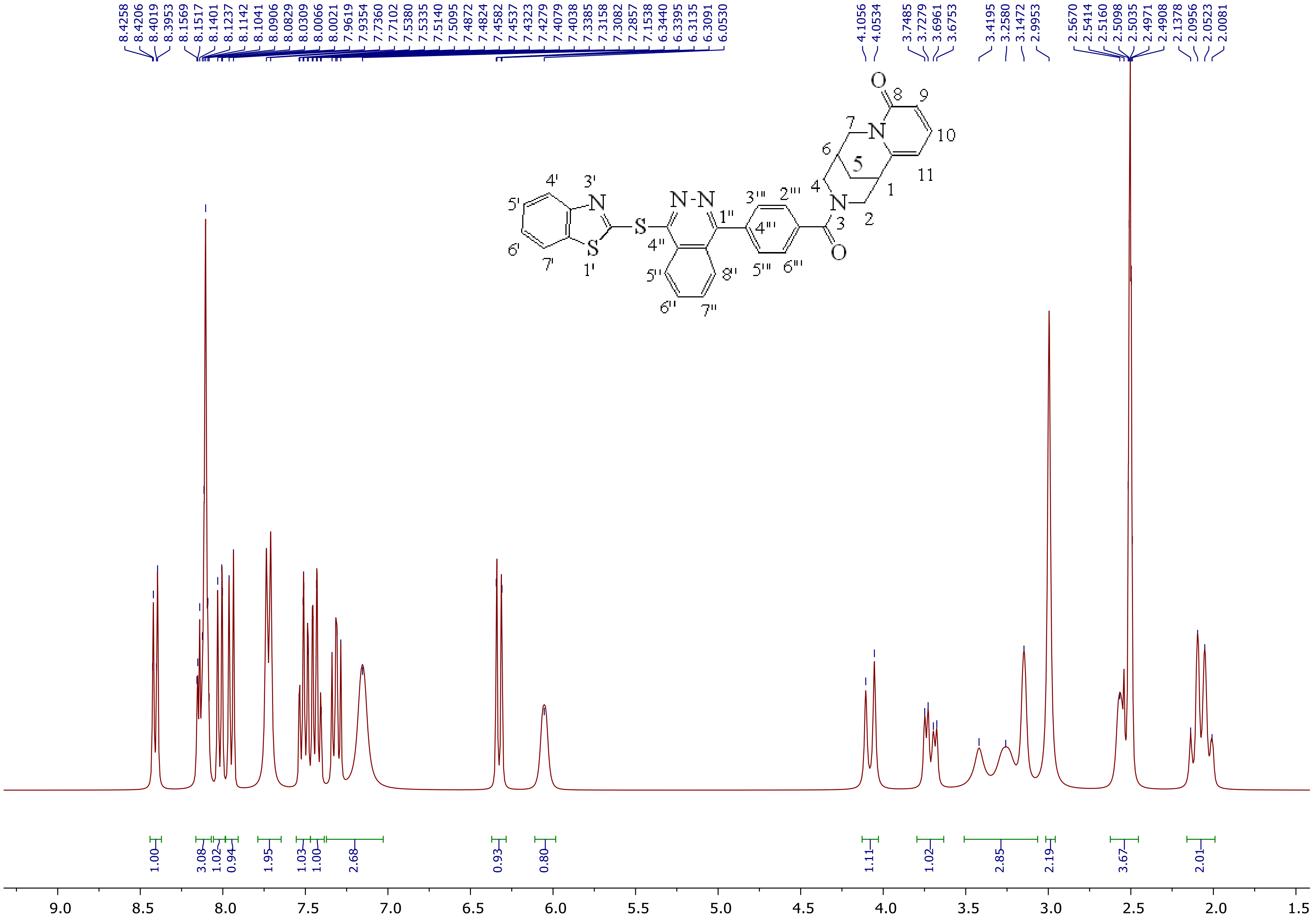
8.4258
8.4206
8.4019
8.3953
8.1569
8.1517
8.1401
8.1237
8.1142
8.1041
8.0906
8.0829
8.0309
8.0066
8.0021
7.9619
7.9354
7.7360
7.7102
7.5380
7.5335
7.5140
7.5095
7.4872
7.4824
7.4582
7.4537
7.4323
7.4279
7.4079
7.4038
7.3385
7.3158
7.3082
7.2857
7.1538
6.3440
6.3395
6.3135
6.3091
6.0530
4.1056
4.0534
3.7485
3.7279
3.6961
3.6753
3.4195
3.2580
3.1472
2.9953
2.5670
2.5414
2.5160
2.5098
2.5035
2.4971
2.4908
2.1378
2.0956
2.0523
2.0081
1.00
3.08
1.02
0.94
1.95
1.03
1.00
2.68
0.93
0.80
1.11
1.02
2.85
2.19
3.67
2.01
9.0
8.5
8.0
7.5
7.0
6.5
6.0
5.5
5.0
4.5
4.0
3.5
3.0
2.5
2.0
1.5

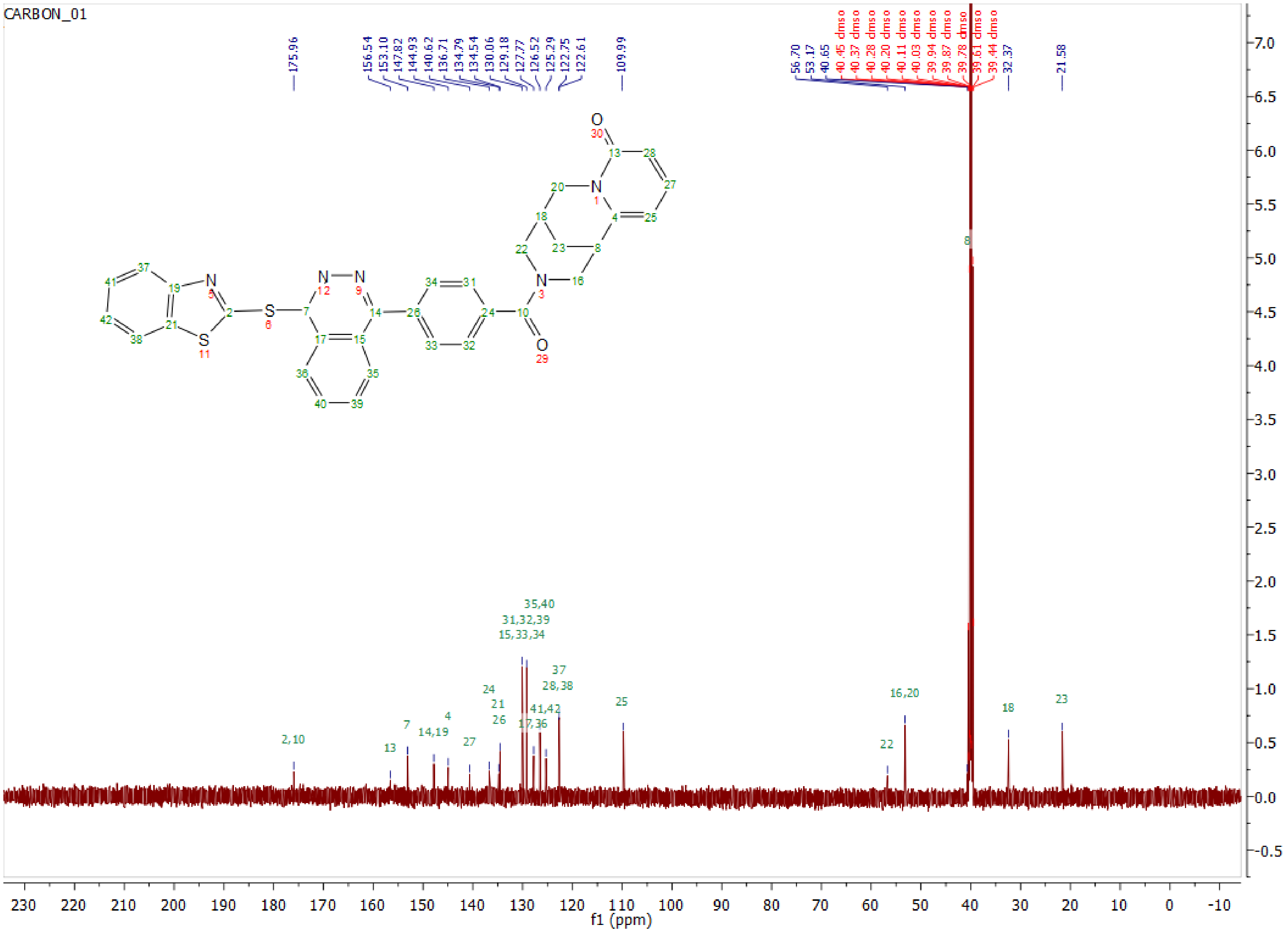

CARBON_01
175.96
156.54
153.10
147.82
144.93
140.62
136.71
134.79
134.54
130.06
129.18
127.77
126.52
125.29
122.75
122.61
109.99
56.70
53.17
40.65
40.45 dmso
40.37 dmso
40.28 dmso
40.20 dmso
40.11 dmso
40.03 dmso
39.94 dmso
39.87 dmso
39.78 dmso
39.61 dmso
39.44 dmso
32.37
21.58
2,10
13
7
14,19
4
27
24
21
26
15,33,34
31,32,39
35,40
17,36
41,42
28,38
37
25
22
16,20
8
18
23
f1 (ppm)

Compound 2i

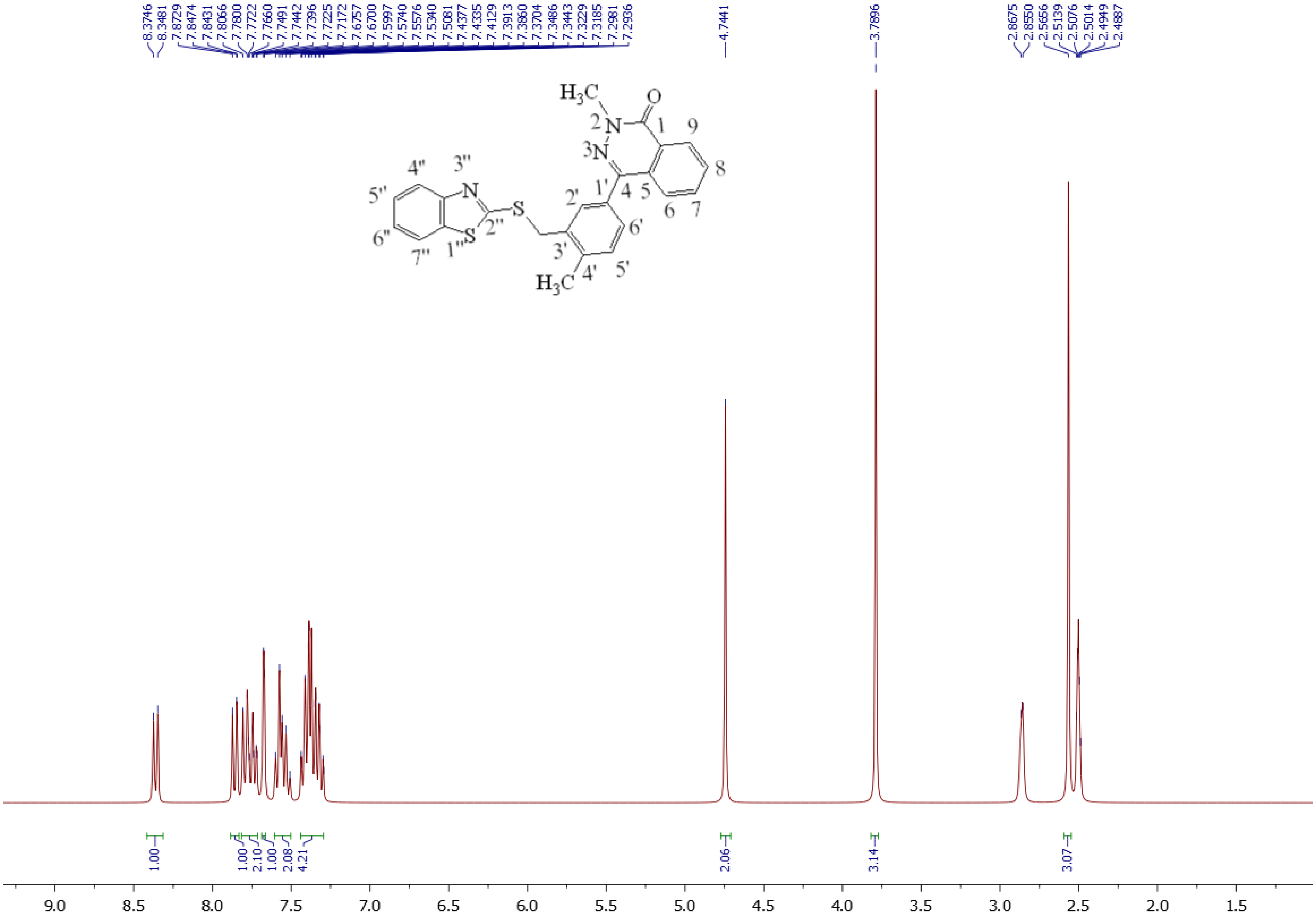
8.3746
8.3481
7.8729
7.8474
7.8431
7.8066
7.7800
7.7722
7.7660
7.7491
7.7442
7.7396
7.7225
7.7172
7.6757
7.6700
7.5997
7.5740
7.5576
7.5340
7.5081
7.4377
7.4335
7.4129
7.3913
7.3860
7.3704
7.3486
7.3443
7.3229
7.3185
7.2981
7.2936
4.7441
3.7896
2.8675
2.8550
2.5656
2.5139
2.5076
2.5014
2.4949
2.4887
H3C
O
N
N
1
2
3
4
5
6
7
8
9
1'
2'
3'
4'
5'
6'
H3C
S
S
N
1''
2''
3''
4''
5''
6''
7''
1.00
1.00
2.10
1.00
2.08
4.21
2.06
3.14
3.07
9.0
8.5
8.0
7.5
7.0
6.5
6.0
5.5
5.0
4.5
4.0
3.5
3.0
2.5
2.0
1.5

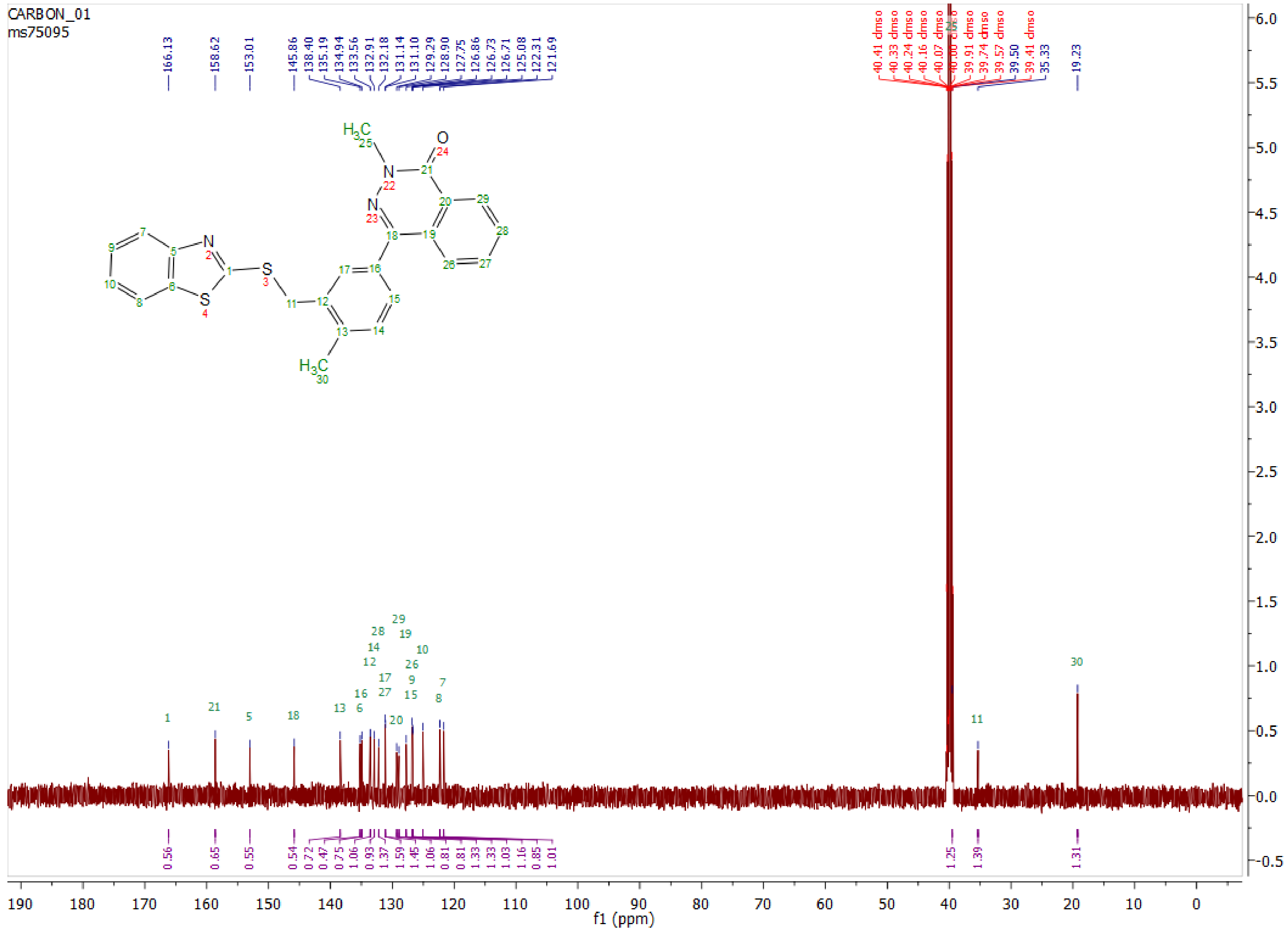
CARBON_01
ms75095
166.13
158.62
153.01
145.86
138.40
135.19
134.94
133.56
132.91
132.18
131.14
131.10
129.29
128.90
127.75
126.86
126.73
126.71
125.08
122.31
121.69
40.41 dmso
40.33 dmso
40.24 dmso
40.16 dmso
40.07 dmso
40.00 dmso
39.91 dmso
39.74 dmso
39.57 dmso
39.50
39.41 dmso
35.33
19.23
f1 (ppm)

Compound 2j

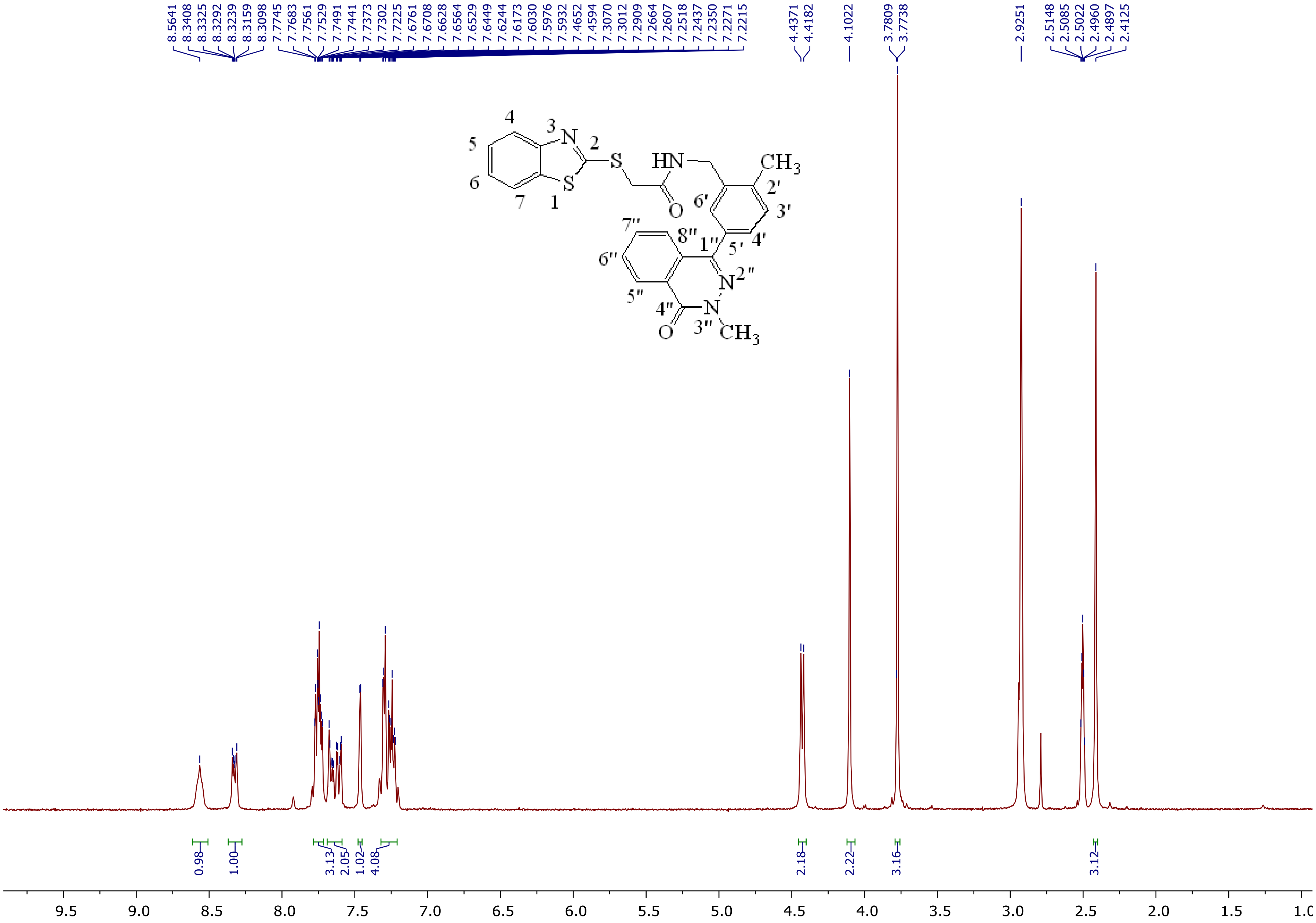

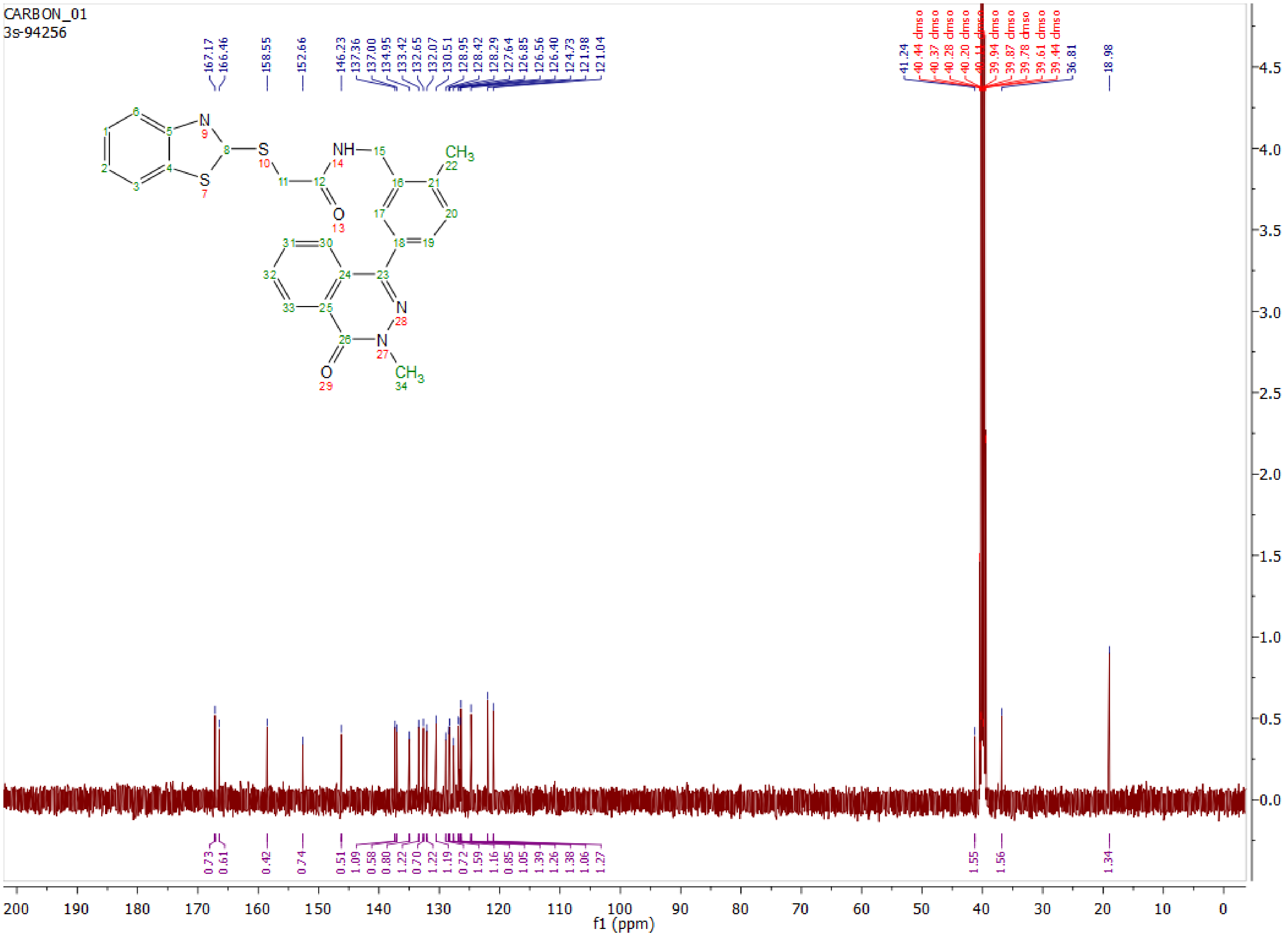
CARBON_01
3s-94256
167.17
166.46
158.55
152.66
146.23
137.36
137.00
134.95
133.42
132.65
132.07
130.51
128.95
128.42
128.29
127.64
126.85
126.56
126.40
124.73
121.98
121.04
41.24
40.44 dmso
40.37 dmso
40.28 dmso
40.20 dmso
39.94 dmso
39.87 dmso
39.78 dmso
39.61 dmso
39.44 dmso
36.81
18.98
CH3
NH
S
N
O
f1 (ppm)

Compound 2k

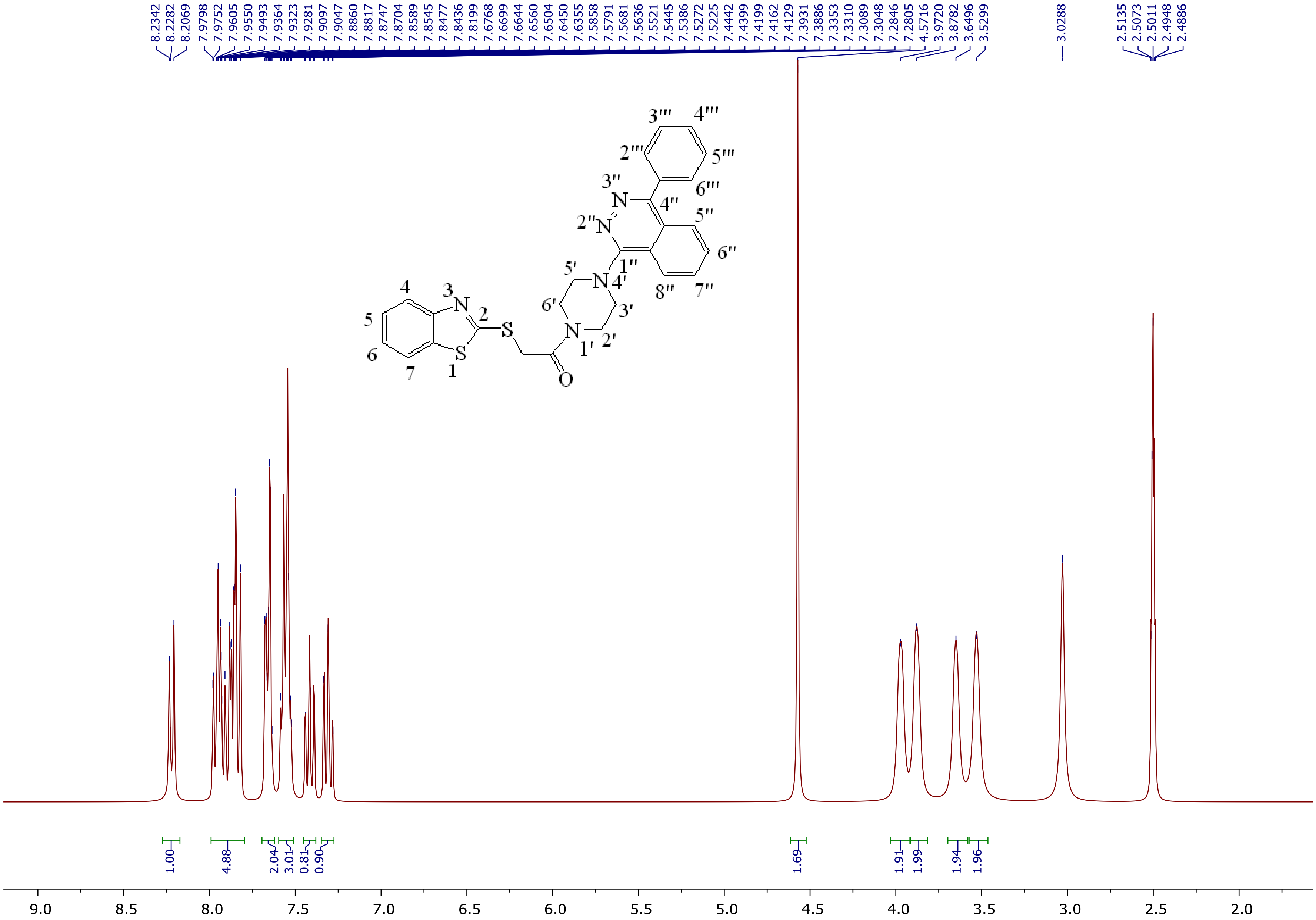

8.2342
8.2282
8.2069
7.9798
7.9752
7.9605
7.9550
7.9493
7.9364
7.9323
7.9281
7.9097
7.9047
7.8860
7.8817
7.8747
7.8704
7.8589
7.8545
7.8477
7.8436
7.8199
7.6768
7.6699
7.6644
7.6560
7.6504
7.6450
7.6355
7.5858
7.5791
7.5681
7.5636
7.5521
7.5445
7.5386
7.5272
7.5225
7.4442
7.4399
7.4199
7.4162
7.4129
7.3931
7.3886
7.3353
7.3310
7.3089
7.3048
7.2846
7.2805
4.5716
3.9720
3.8782
3.6496
3.5299
3.0288
2.5135
2.5073
2.5011
2.4948
2.4886
1.00
4.88
2.04
3.01
0.81
0.90
1.69
1.91
1.99
1.94
1.96
9.0
8.5
8.0
7.5
7.0
6.5
6.0
5.5
5.0
4.5
4.0
3.5
3.0
2.5
2.0

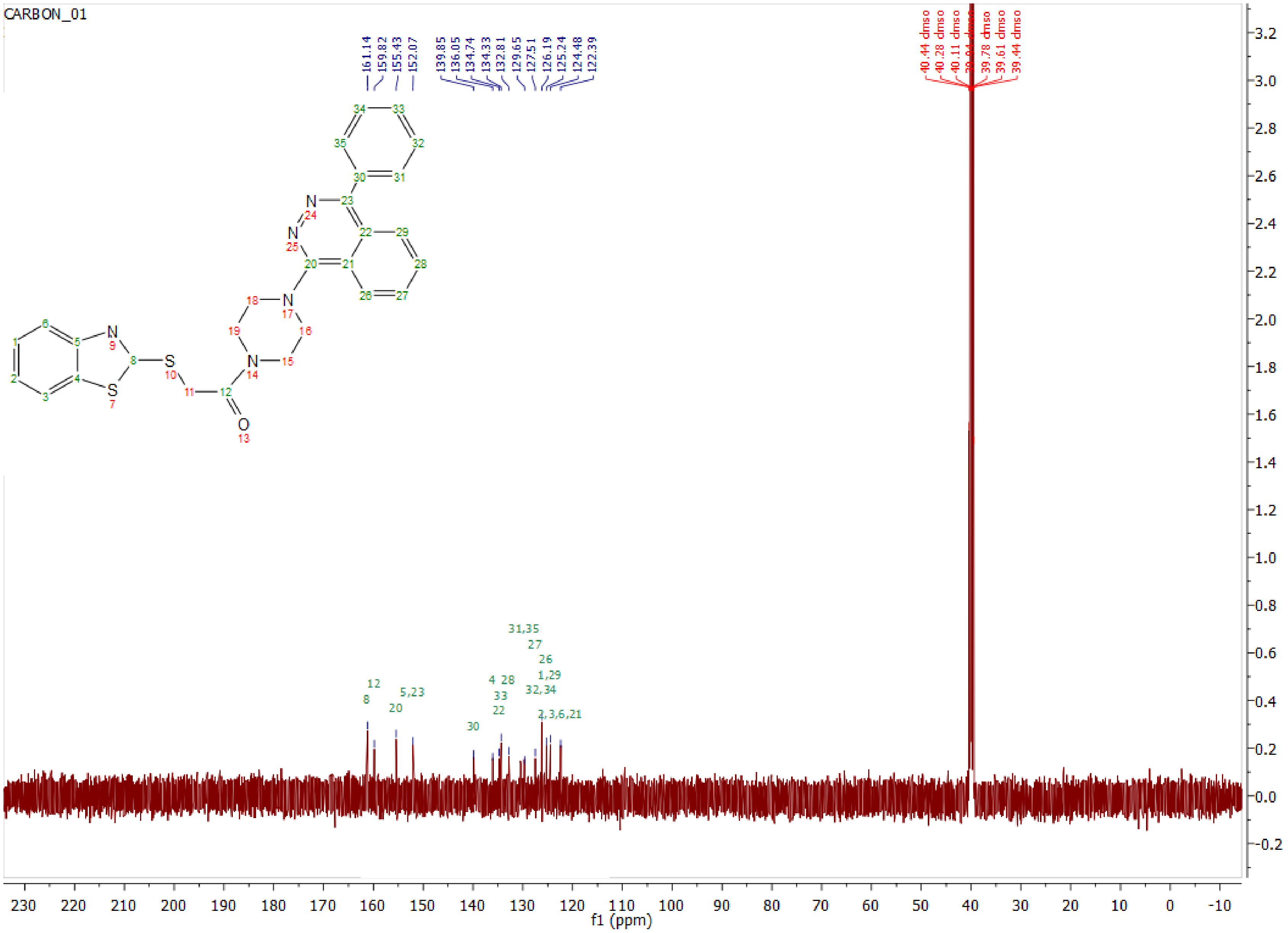

CARBON_01
161.14
159.82
155.43
152.07
139.85
136.05
134.74
134.33
132.81
129.65
127.51
126.19
125.24
124.48
122.39
40.44 dmso
40.28 dmso
40.11 dmso
39.78 dmso
39.61 dmso
39.44 dmso
f1 (ppm)

Compound 2l

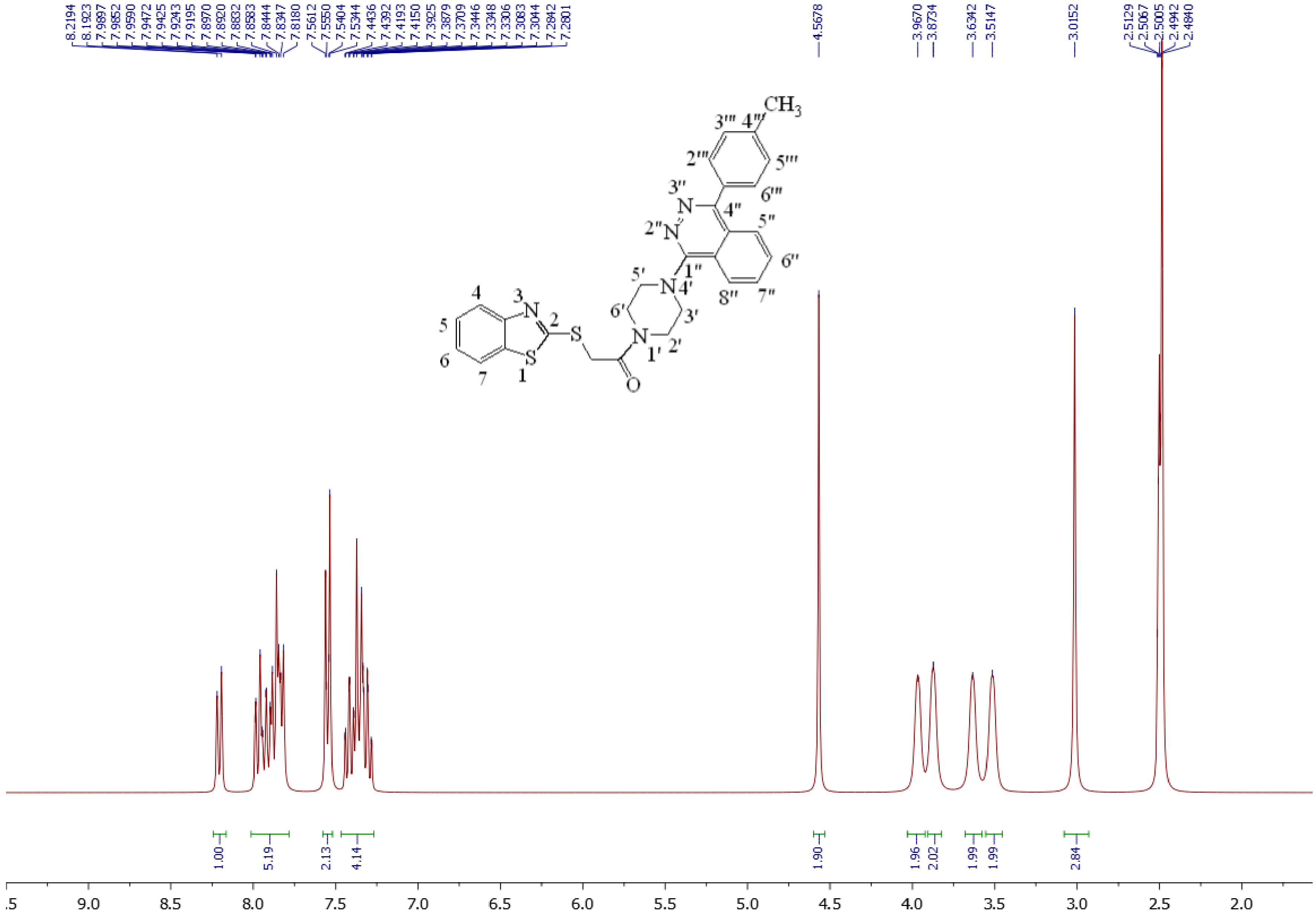
8.2194
8.1923
7.9897
7.9852
7.9590
7.9472
7.9425
7.9243
7.9195
7.8970
7.8920
7.8832
7.8583
7.8444
7.8347
7.8180
7.5612
7.5550
7.5404
7.5344
7.4436
7.4392
7.4193
7.4150
7.3925
7.3879
7.3709
7.3446
7.3348
7.3306
7.3083
7.3044
7.2842
7.2801
4.5678
3.9670
3.8734
3.6342
3.5147
3.0152
2.5129
2.5067
2.5005
2.4942
2.4840
CH3
N
S
O
1.00
5.19
2.13
4.14
1.90
1.96
2.02
1.99
1.99
2.84
9.0
8.5
8.0
7.5
7.0
6.5
6.0
5.5
5.0
4.5
4.0
3.5
3.0
2.5
2.0

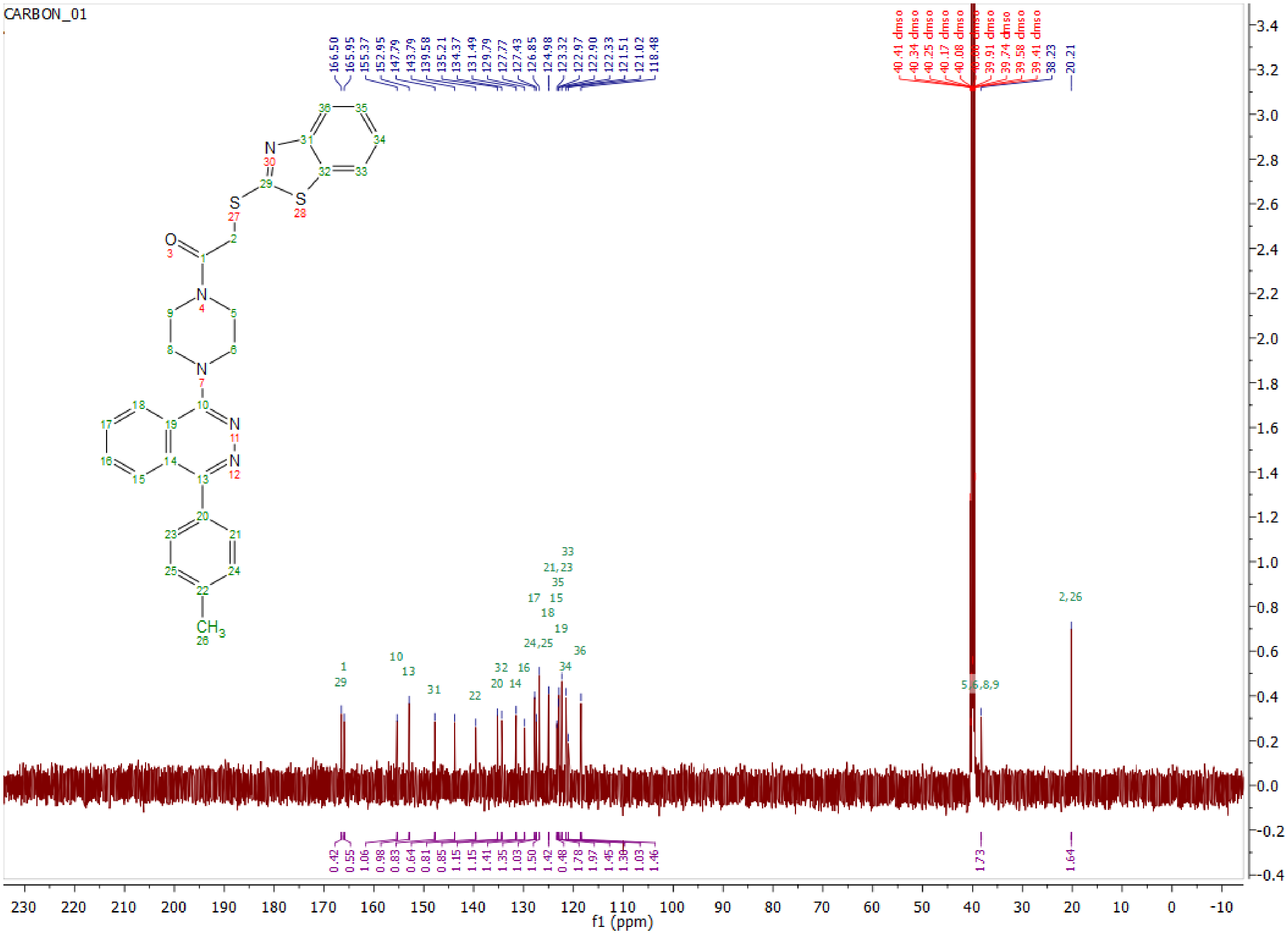

CARBON_01
f1 (ppm)

Compound 2m

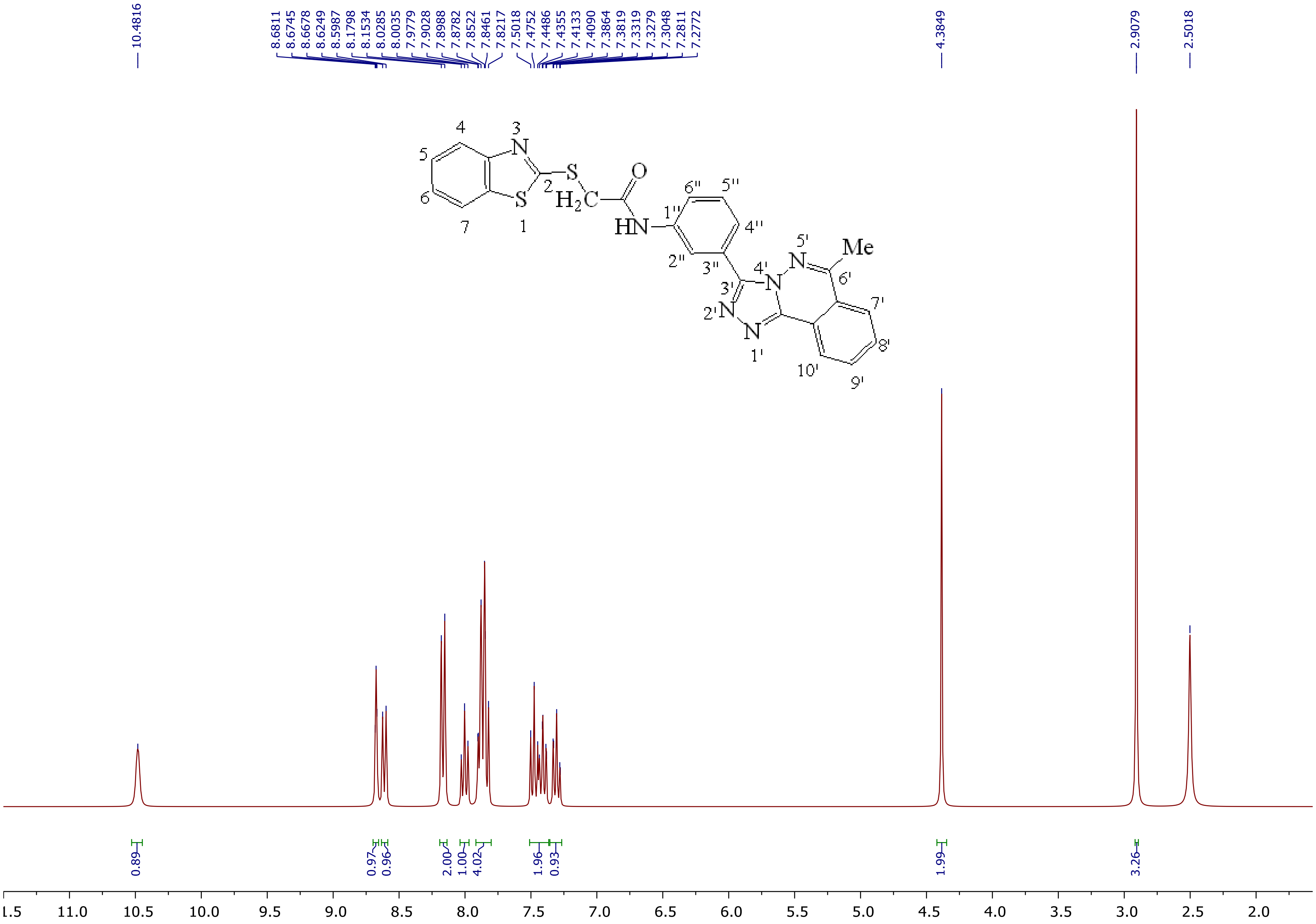

10.4816
8.6811
8.6745
8.6678
8.6249
8.5987
8.1798
8.1534
8.0285
8.0035
7.9779
7.9028
7.8988
7.8782
7.8522
7.8461
7.8217
7.5018
7.4752
7.4486
7.4355
7.4133
7.4090
7.3864
7.3819
7.3319
7.3279
7.3048
7.2811
7.2772
4.3849
2.9079
2.5018
0.89
0.97
0.96
2.00
1.00
4.02
1.96
0.93
1.99
3.26
11.0
10.5
10.0
9.5
9.0
8.5
8.0
7.5
7.0
6.5
6.0
5.5
5.0
4.5
4.0
3.5
3.0
2.5
2.0

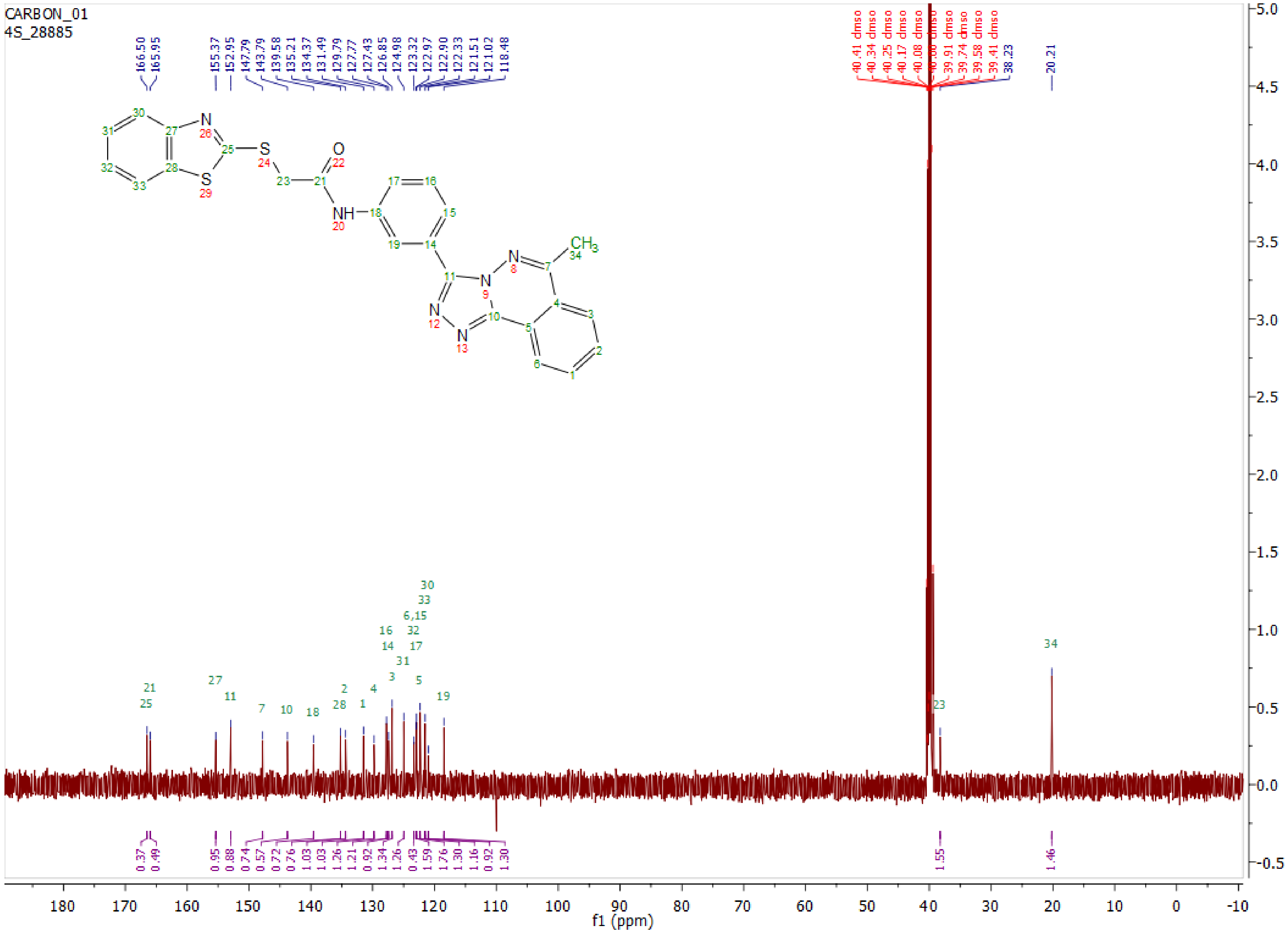
CARBON_01
4S_28885
166.50
165.95
155.37
152.95
147.79
143.79
139.58
135.21
134.37
131.49
129.79
127.77
127.43
126.85
124.98
123.32
122.97
122.90
122.33
121.51
121.02
118.48
40.41 dmso
40.34 dmso
40.25 dmso
40.17 dmso
40.08 dmso
40.00 dmso
39.91 dmso
39.74 dmso
39.58 dmso
39.41 dmso
38.23
20.21
CH3
f1 (ppm)

Compound 2n

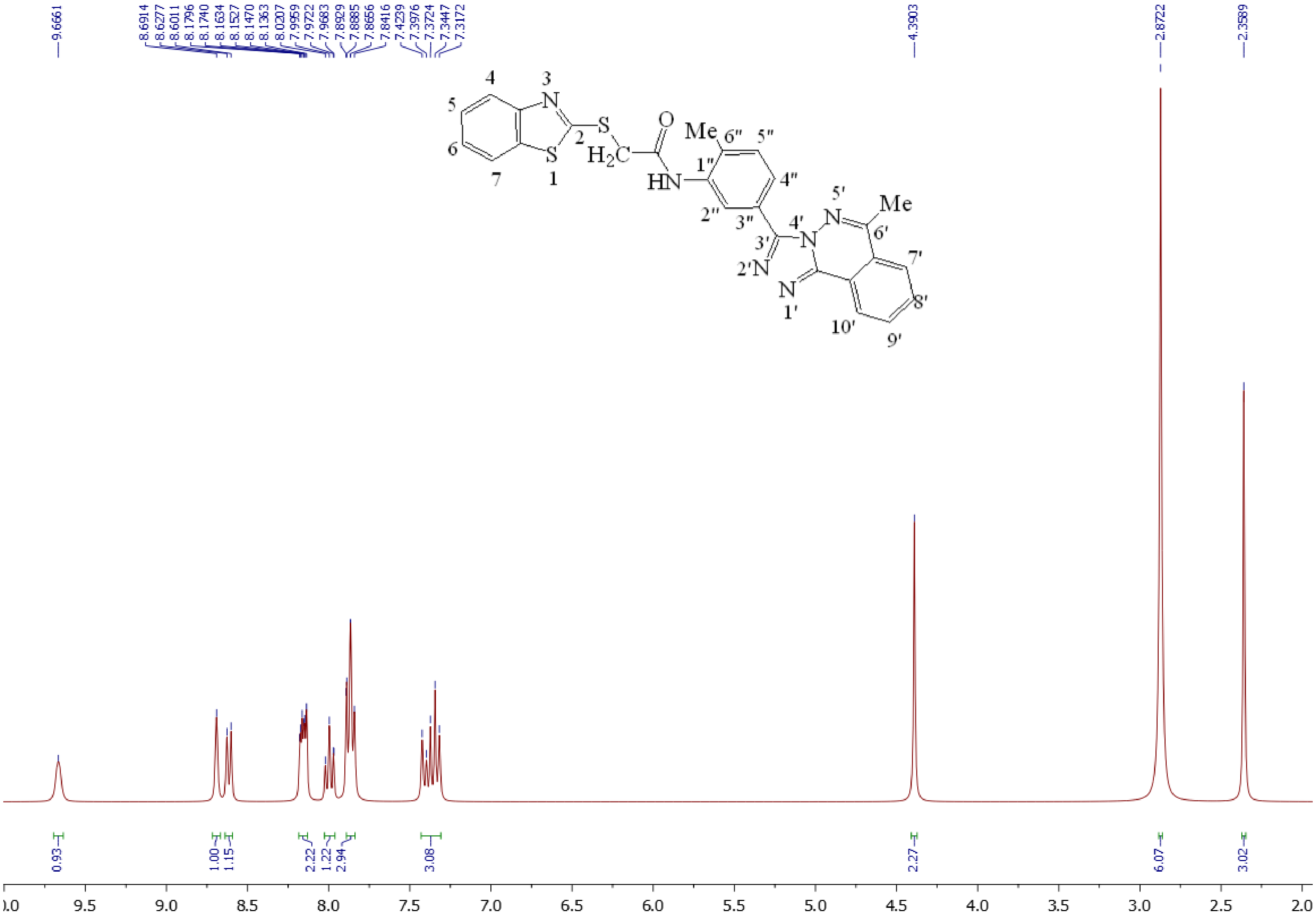

9.6661
8.6914
8.6277
8.6011
8.1796
8.1740
8.1634
8.1527
8.1470
8.1363
8.0207
7.9959
7.9722
7.9683
7.8929
7.8885
7.8656
7.8416
7.4239
7.3976
7.3724
7.3447
7.3172
4.3903
2.8722
2.3589
Me
Me
HN
H$_2$C
O
S
N
0.93
1.00
1.15
2.22
1.22
2.94
3.08
2.27
6.07
3.02
9.5
9.0
8.5
8.0
7.5
7.0
6.5
6.0
5.5
5.0
4.5
4.0
3.5
3.0
2.5
2.0

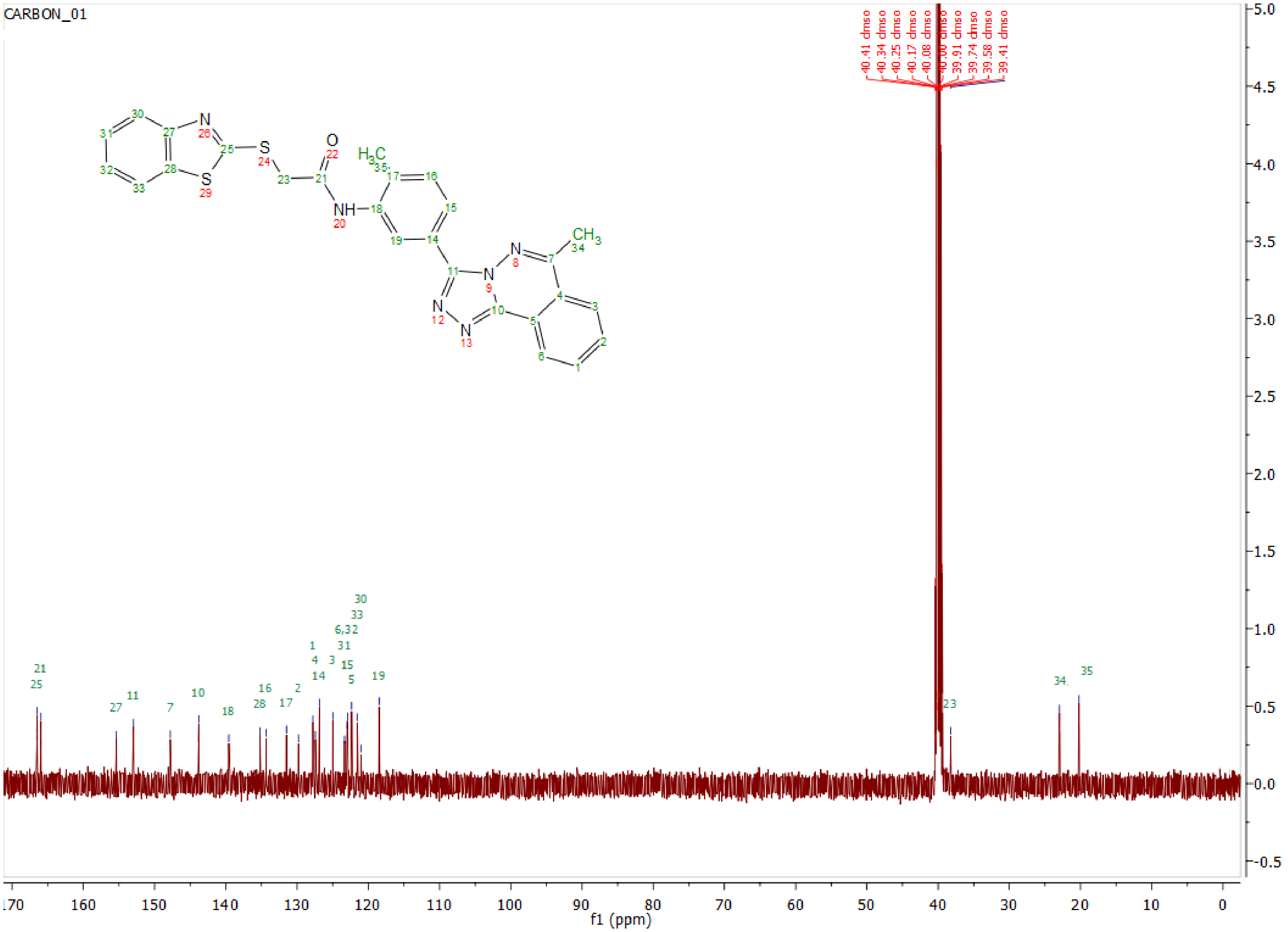

CARBON_01
40.41 dmso
40.34 dmso
40.25 dmso
40.17 dmso
40.08 dmso
40.00 dmso
39.91 dmso
39.74 dmso
39.58 dmso
39.41 dmso
f1 (ppm)

Compound 2o

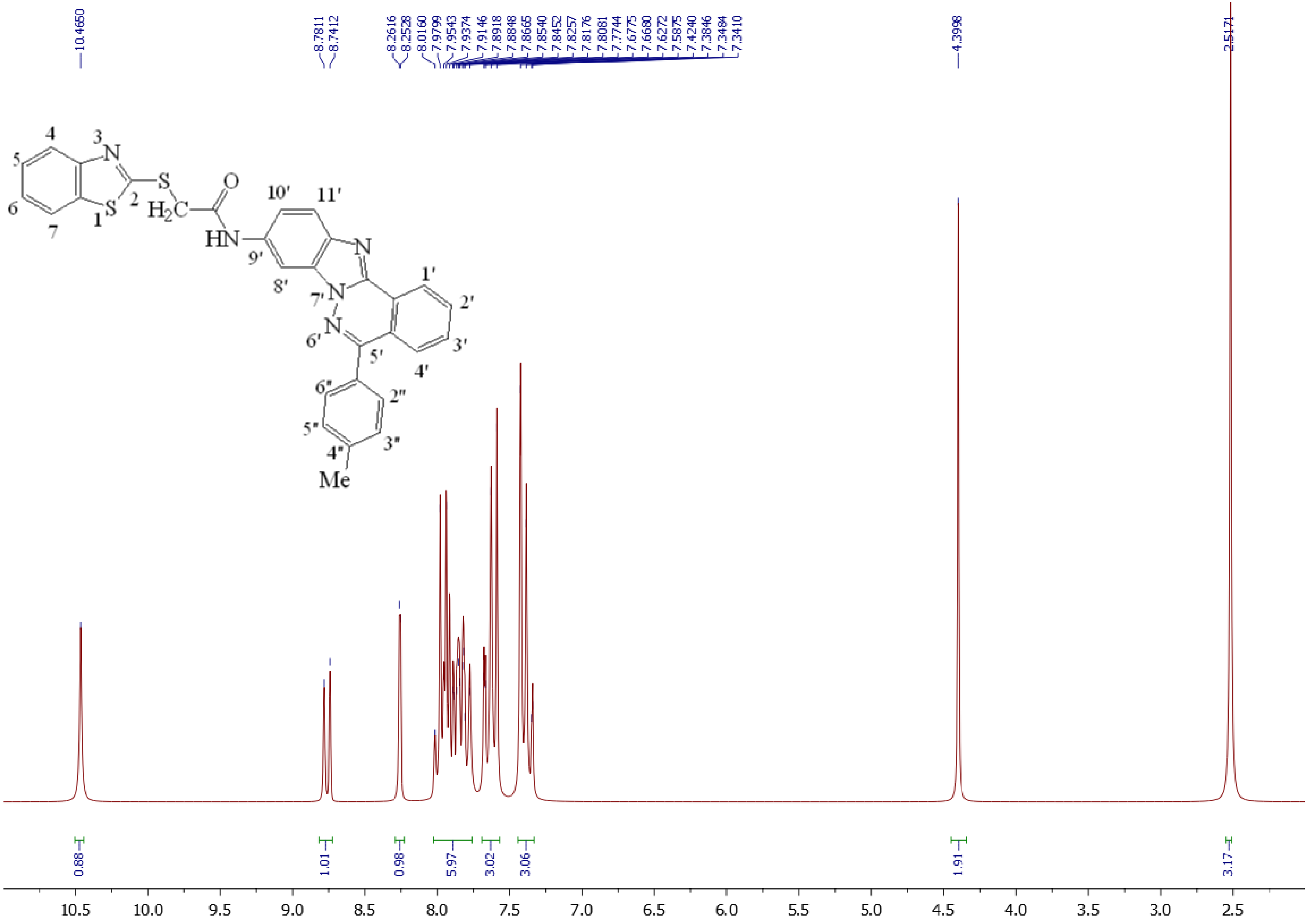

10.4650
8.7811
8.7412
8.2616
8.2528
8.0160
7.9799
7.9543
7.9374
7.9146
7.8918
7.8848
7.8665
7.8540
7.8452
7.8257
7.8176
7.8081
7.7744
7.6775
7.6680
7.6272
7.5875
7.4240
7.3846
7.3484
7.3410
4.3998
2.5171
0.88
1.01
0.98
5.97
3.02
3.06
1.91
3.17
10.5
10.0
9.5
9.0
8.5
8.0
7.5
7.0
6.5
6.0
5.5
5.0
4.5
4.0
3.5
3.0
2.5
HN
Me

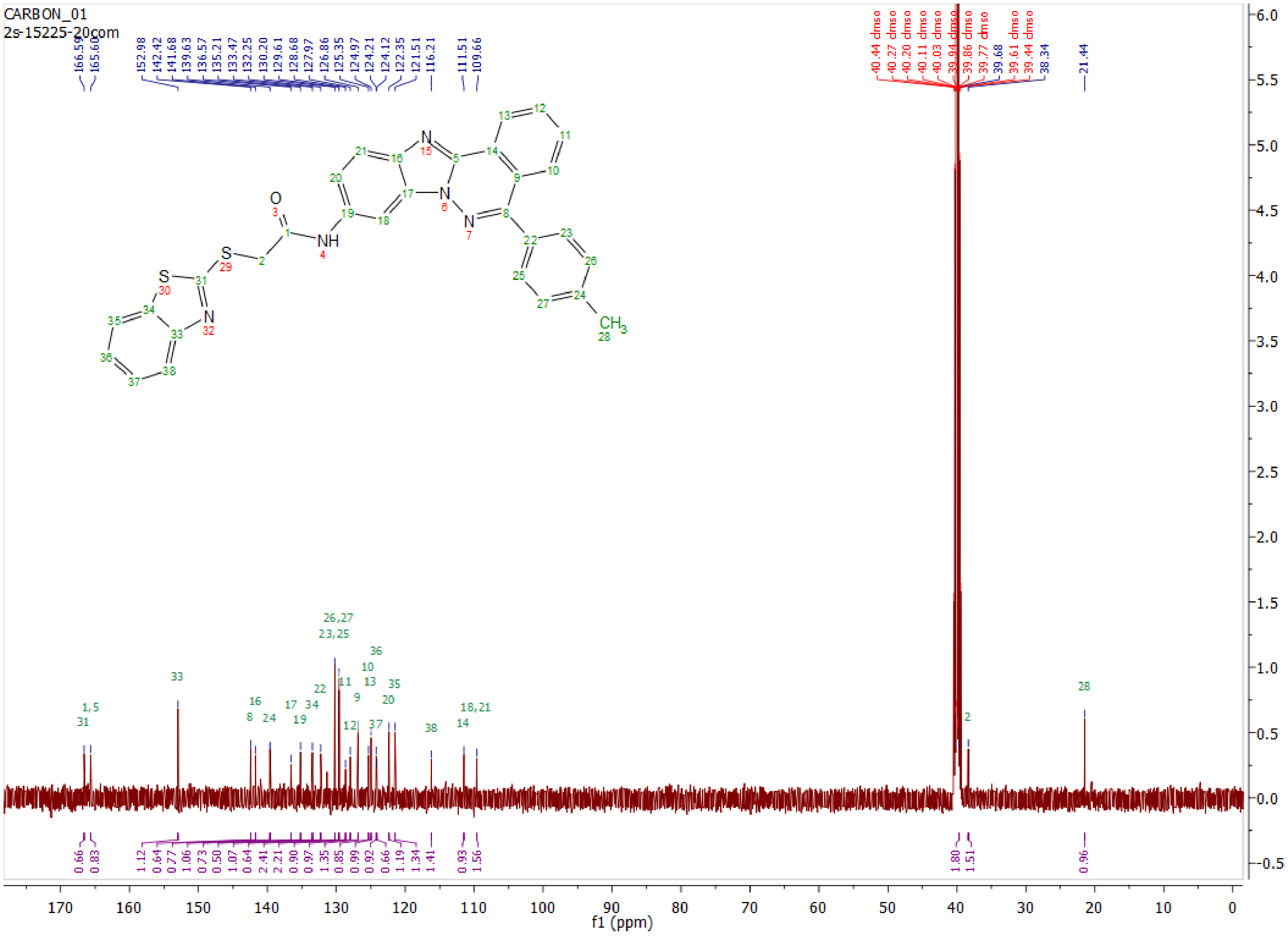
CARBON_01
2s-15225-20com
f1 (ppm)

Compound 3a

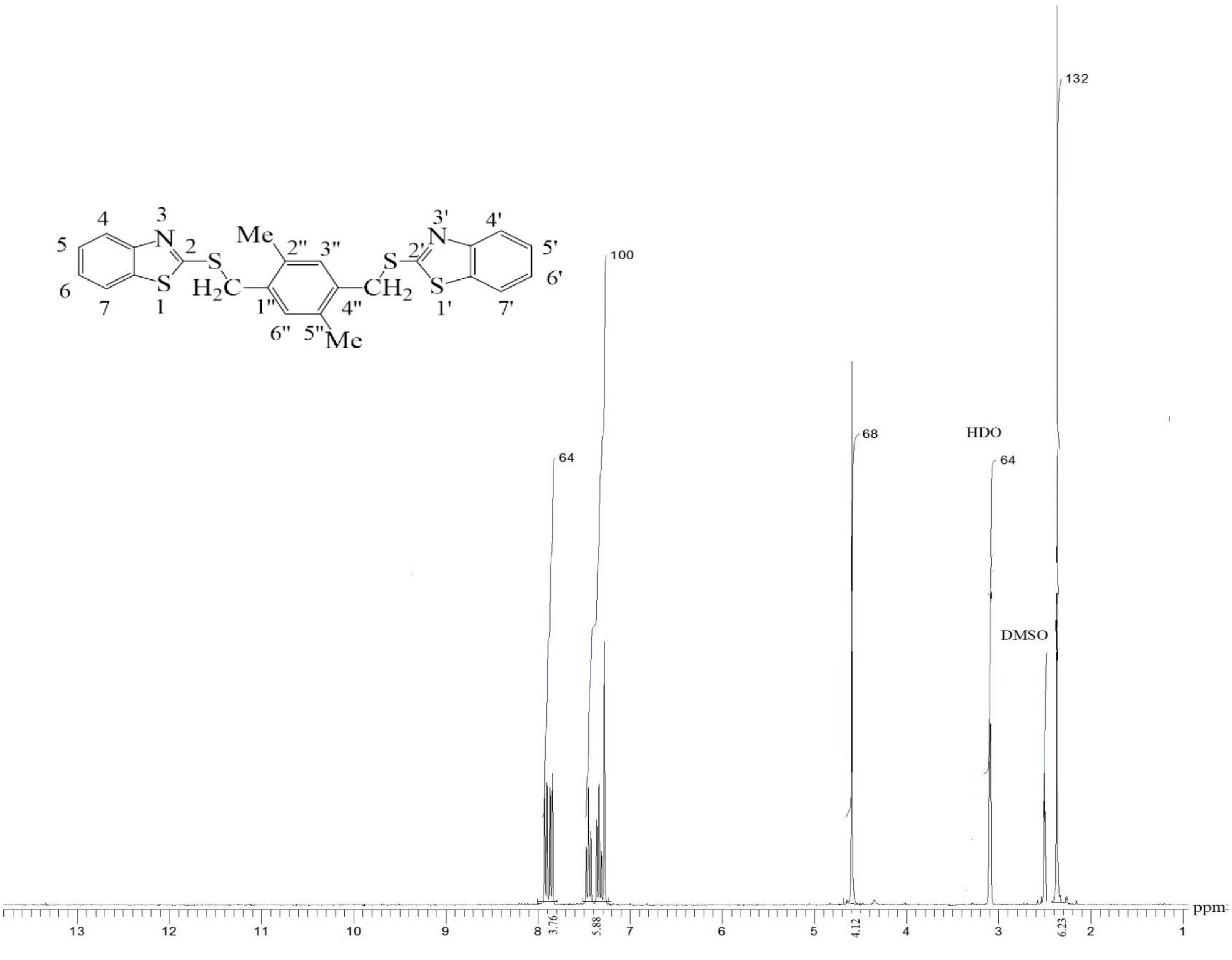

132
100
68
64
64
HDO
DMSO
3.76
5.88
4.12
6.23
13
12
11
10
9
8
7
6
5
4
3
2
1
ppm

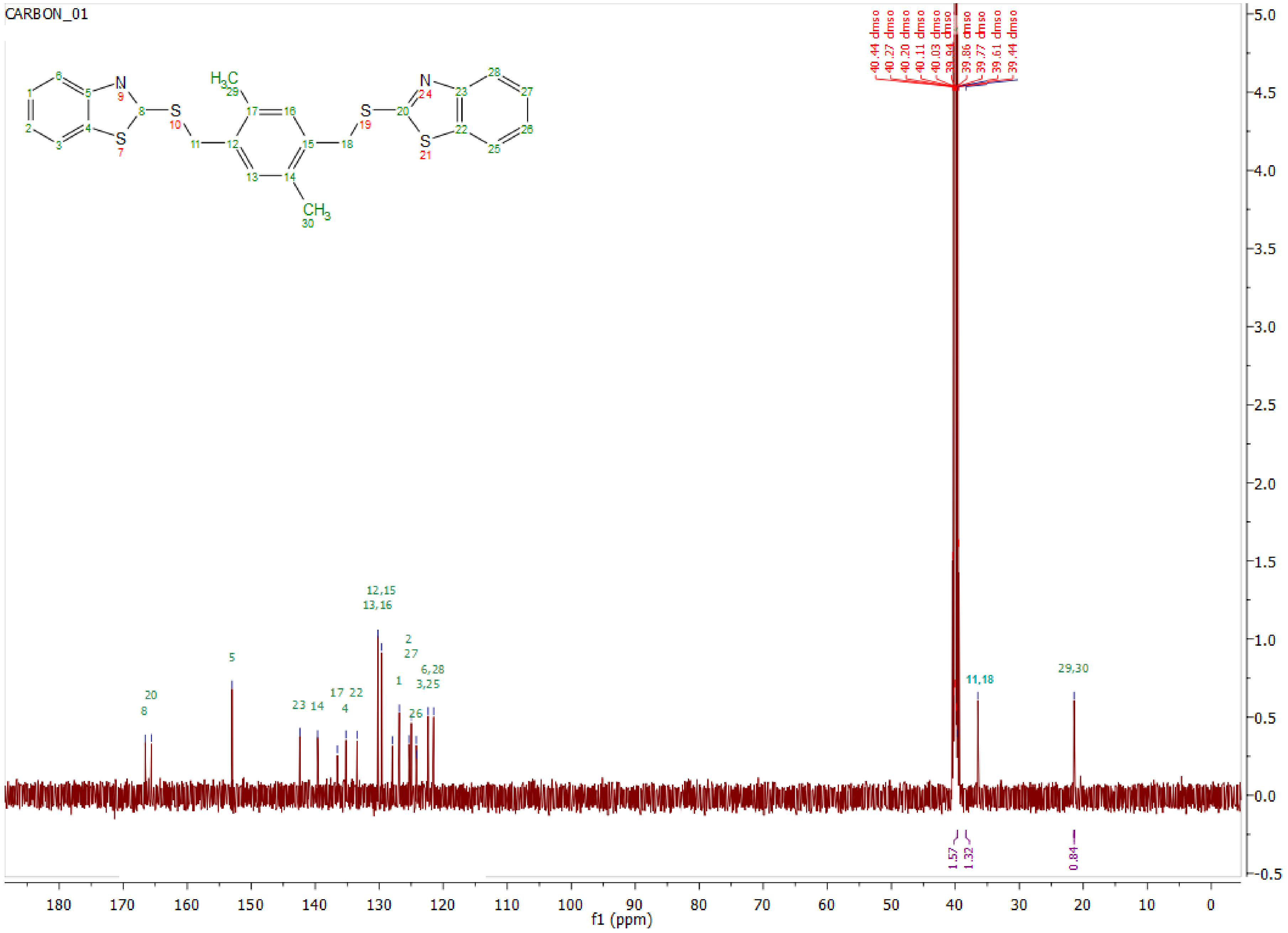

CARBON_01
40.44 dmso
40.27 dmso
40.20 dmso
40.11 dmso
40.03 dmso
39.94 dmso
39.86 dmso
39.77 dmso
39.61 dmso
39.44 dmso
20
8
5
23 14
17 22
4
12,15
13,16
1
2
27
26
6,28
3,25
11,18
29,30
1.57
1.32
0.84
f1 (ppm)

Compound 3b

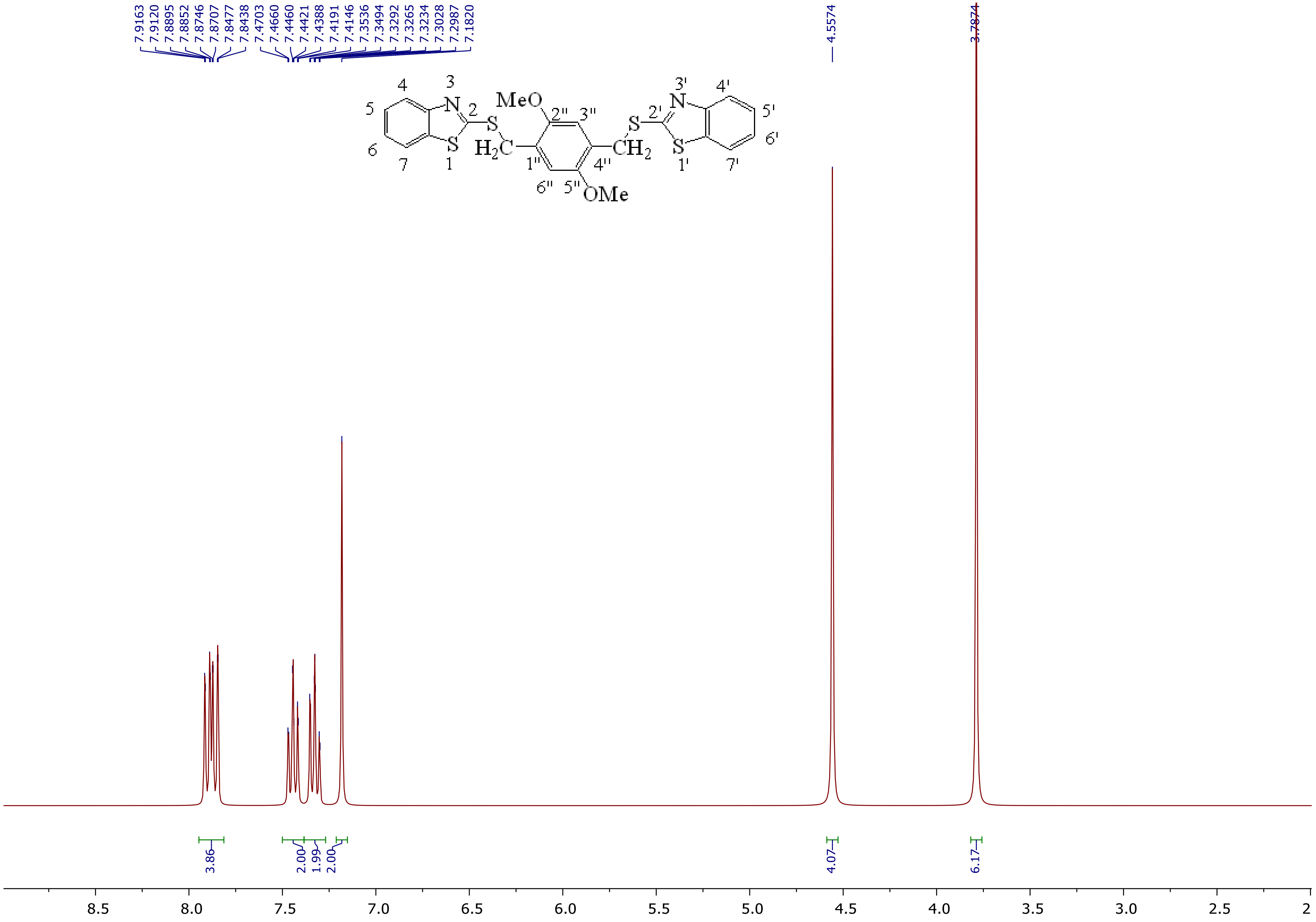

7.9163
7.9120
7.8895
7.8852
7.8746
7.8707
7.8477
7.8438
7.4703
7.4660
7.4460
7.4421
7.4388
7.4191
7.4146
7.3536
7.3494
7.3292
7.3265
7.3234
7.3028
7.2987
7.1820
4.5574
3.7874
3.86
2.00
1.99
2.00
4.07
6.17
8.5
8.0
7.5
7.0
6.5
6.0
5.5
5.0
4.5
4.0
3.5
3.0
2.5
2

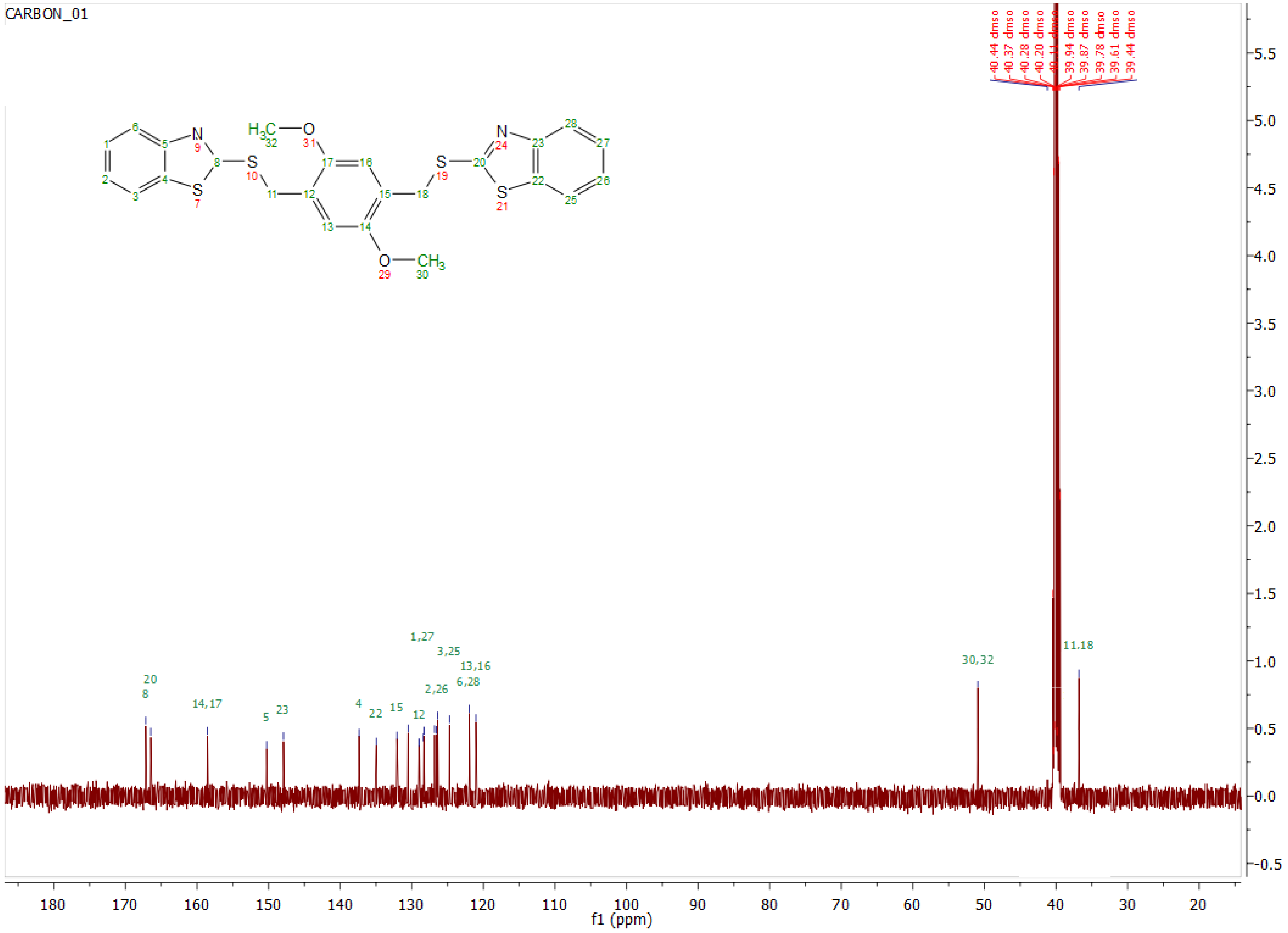
CARBON_01
40.44 dmso
40.37 dmso
40.28 dmso
40.20 dmso
39.94 dmso
39.87 dmso
39.78 dmso
39.61 dmso
39.44 dmso
20
8
14,17
5
23
4
22
15
12
1,27
3,25
2,26
13,16
6,28
30,32
11.18
f1 (ppm)

Compound 5

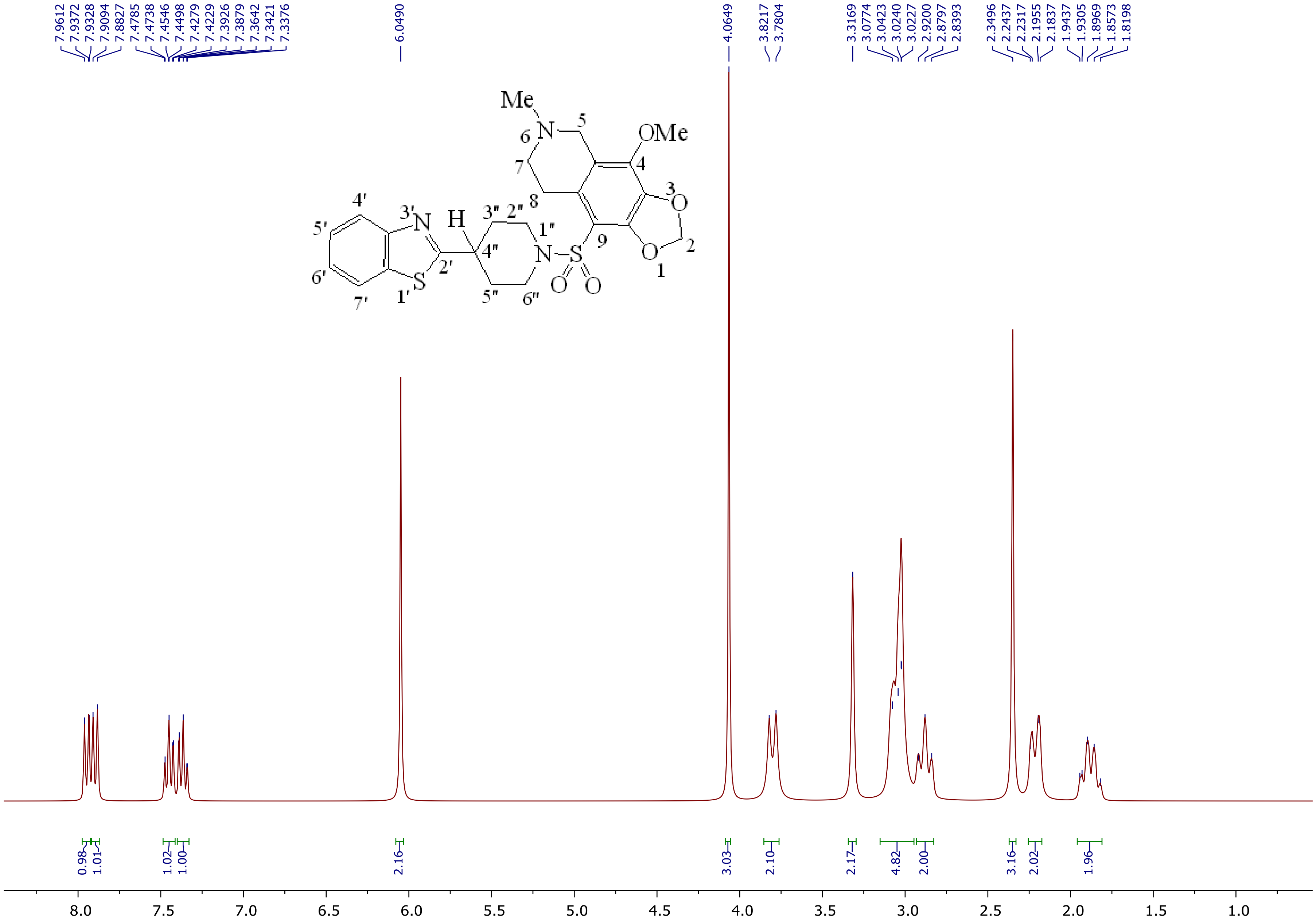

7.9612
7.9372
7.9328
7.9094
7.8827
7.4785
7.4738
7.4546
7.4498
7.4279
7.4229
7.3926
7.3879
7.3642
7.3421
7.3376
6.0490
4.0649
3.8217
3.7804
3.3169
3.0774
3.0423
3.0240
3.0227
2.9200
2.8797
2.8393
2.3496
2.2437
2.2317
2.1955
2.1837
1.9437
1.9305
1.8969
1.8573
1.8198
Me
OMe
0.98
1.01
1.02
1.00
2.16
3.03
2.10
2.17
4.82
2.00
3.16
2.02
1.96
8.0
7.5
7.0
6.5
6.0
5.5
5.0
4.5
4.0
3.5
3.0
2.5
2.0
1.5
1.0

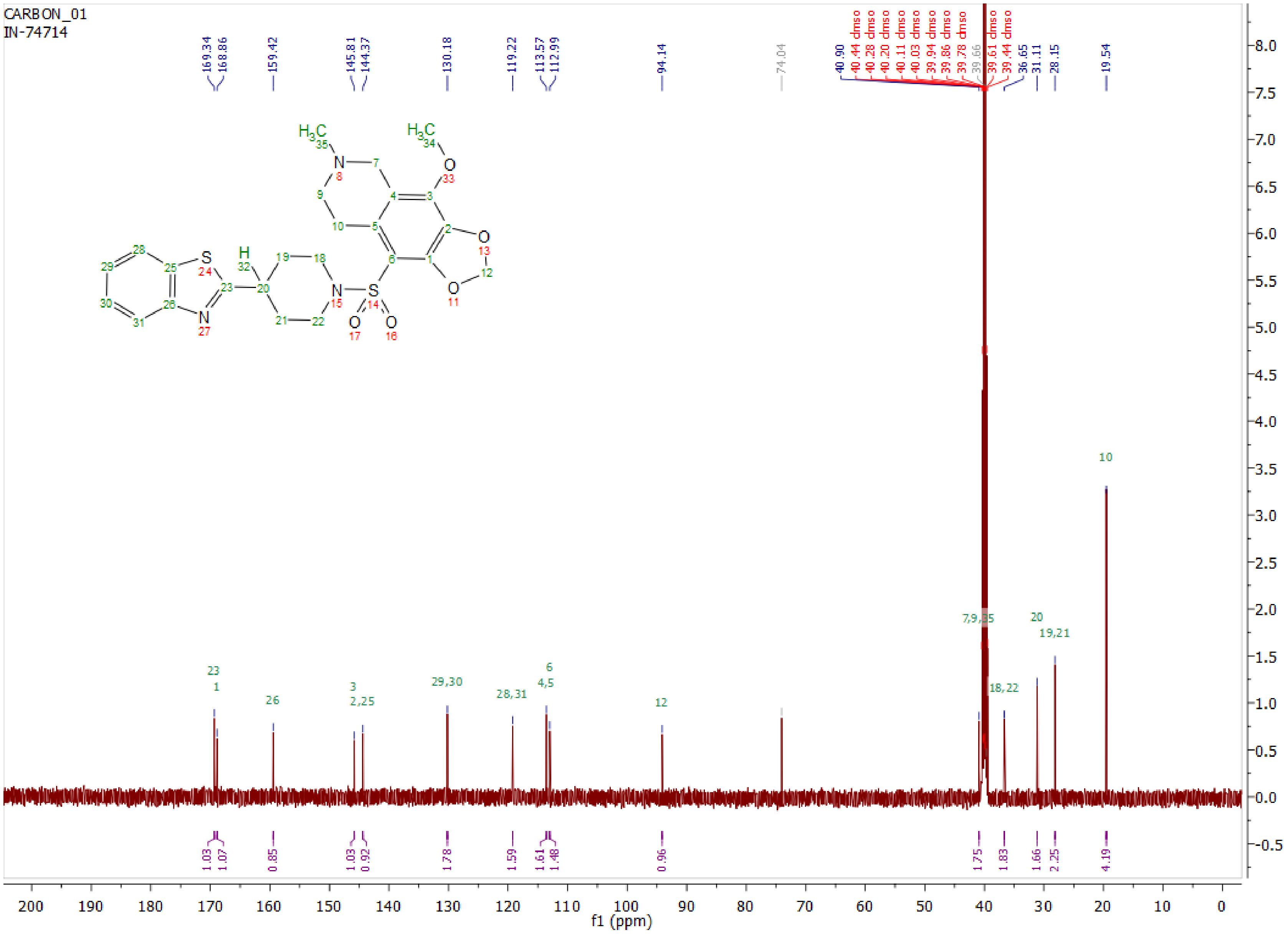

CARBON_01
IN-74714
169.34
168.86
159.42
145.81
144.37
130.18
119.22
113.57
112.99
94.14
74.04
40.90
40.44 dmso
40.28 dmso
40.20 dmso
40.11 dmso
40.03 dmso
39.94 dmso
39.86 dmso
39.78 dmso
39.66
39.61 dmso
39.44 dmso
36.65
31.11
28.15
19.54
f1 (ppm)

Compound 6

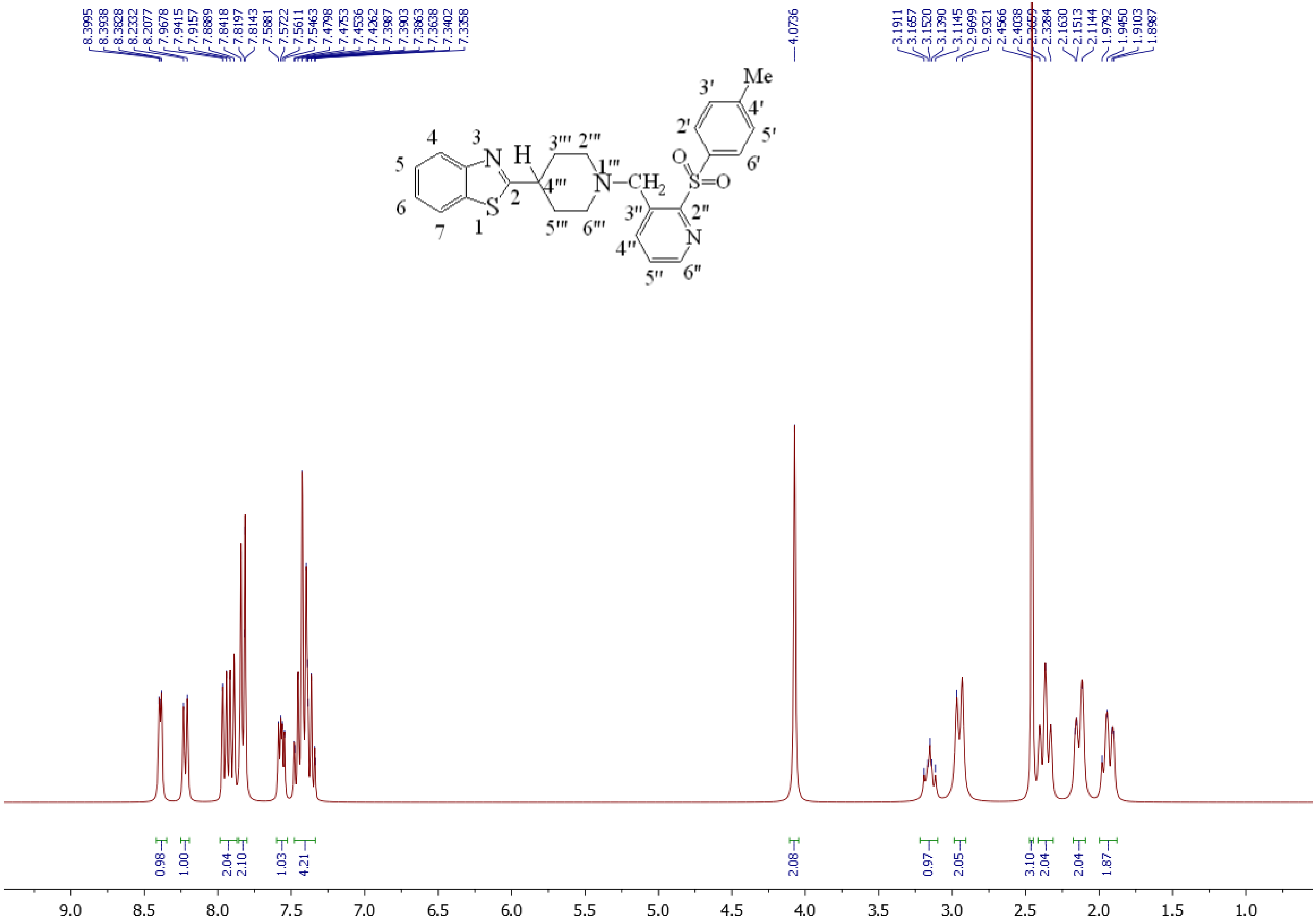

8.3995
8.3938
8.3828
8.2332
8.2077
7.9678
7.9415
7.9157
7.8889
7.8418
7.8197
7.8143
7.5881
7.5722
7.5611
7.5463
7.4798
7.4753
7.4536
7.4262
7.3987
7.3903
7.3863
7.3638
7.3402
7.3358
4.0736
3.1911
3.1657
3.1520
3.1390
3.1145
2.9699
2.9321
2.4566
2.4038
2.3284
2.1630
2.1513
2.1144
1.9792
1.9450
1.9103
1.8987
Me
3′
4′
2′
5′
6′
4
3
N
H
3‴
2‴
1‴
5
2
4‴
N
CH_2
O
S
O
6
S
1
7
5‴
6‴
3″
2″
N
4″
5″
6″
0.98
1.00
2.04
2.10
1.03
4.21
2.08
0.97
2.05
3.10
2.04
2.04
1.87
9.0
8.5
8.0
7.5
7.0
6.5
6.0
5.5
5.0
4.5
4.0
3.5
3.0
2.5
2.0
1.5
1.0

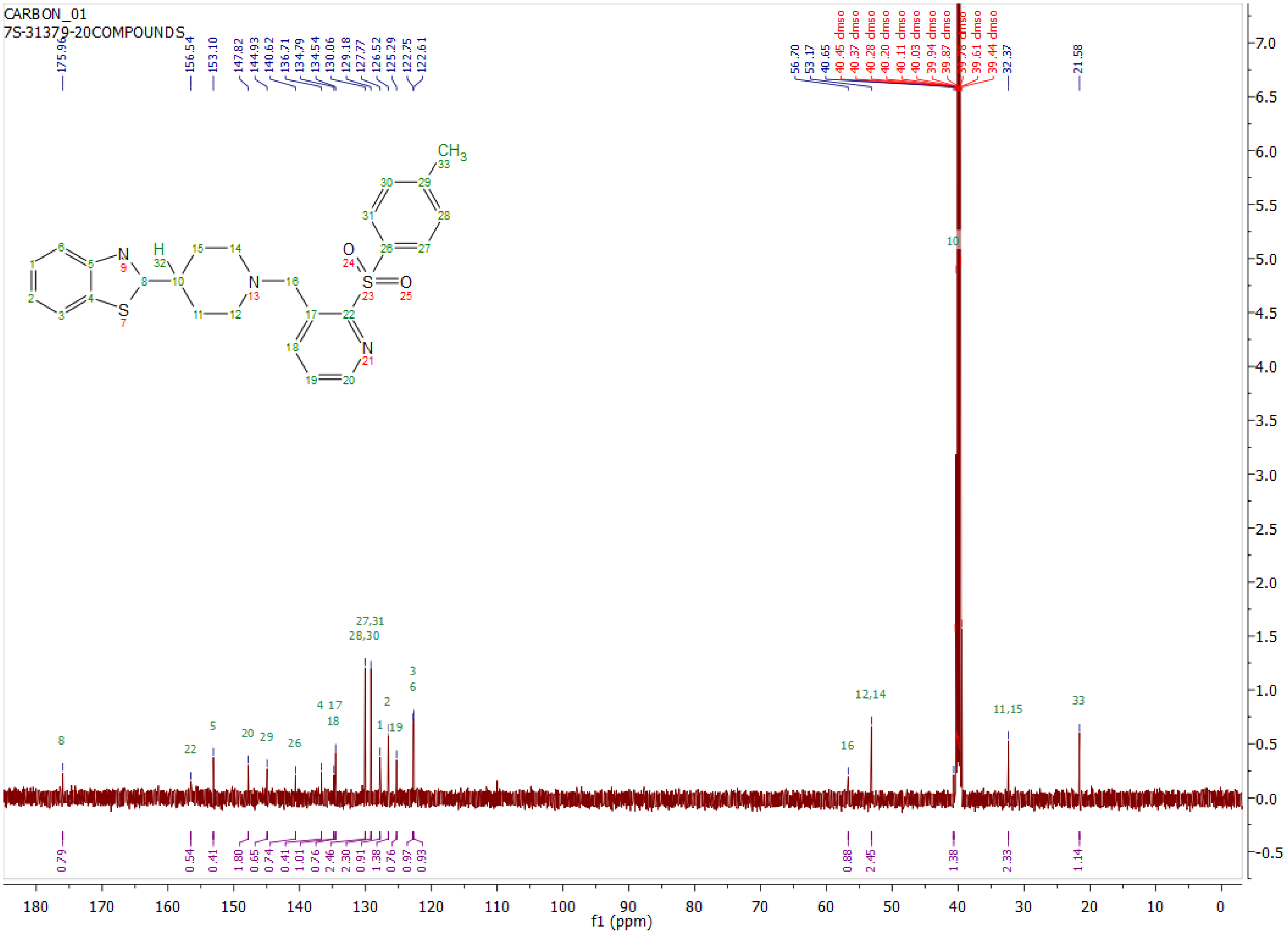
CARBON_01
7S-31379-20COMPOUNDS
175.96
156.54
153.10
147.82
144.93
140.62
136.71
134.79
134.54
130.06
129.18
127.77
126.52
125.29
122.75
122.61
56.70
53.17
40.65
32.37
21.58
f1 (ppm)

Compound 8

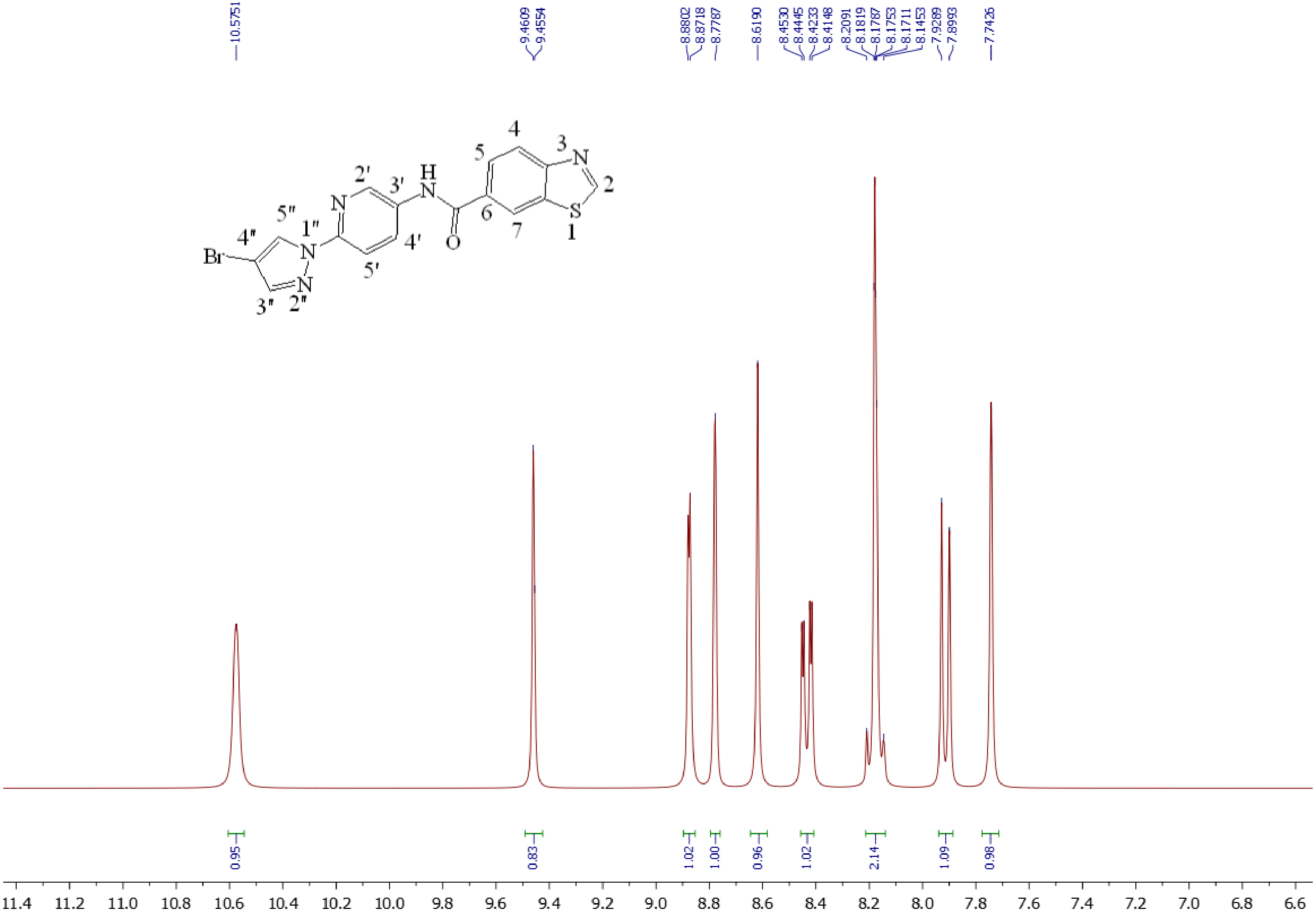

10.5751
9.4609
9.4554
8.8802
8.8718
8.7787
8.6190
8.4530
8.4445
8.4233
8.4148
8.2091
8.1819
8.1787
8.1753
8.1711
8.1453
7.9289
7.8993
7.7426
Br
N
H
O
S
0.95
0.83
1.02
1.00
0.96
1.02
2.14
1.09
0.98
11.4
11.2
11.0
10.8
10.6
10.4
10.2
10.0
9.8
9.6
9.4
9.2
9.0
8.8
8.6
8.4
8.2
8.0
7.8
7.6
7.4
7.2
7.0
6.8
6.6

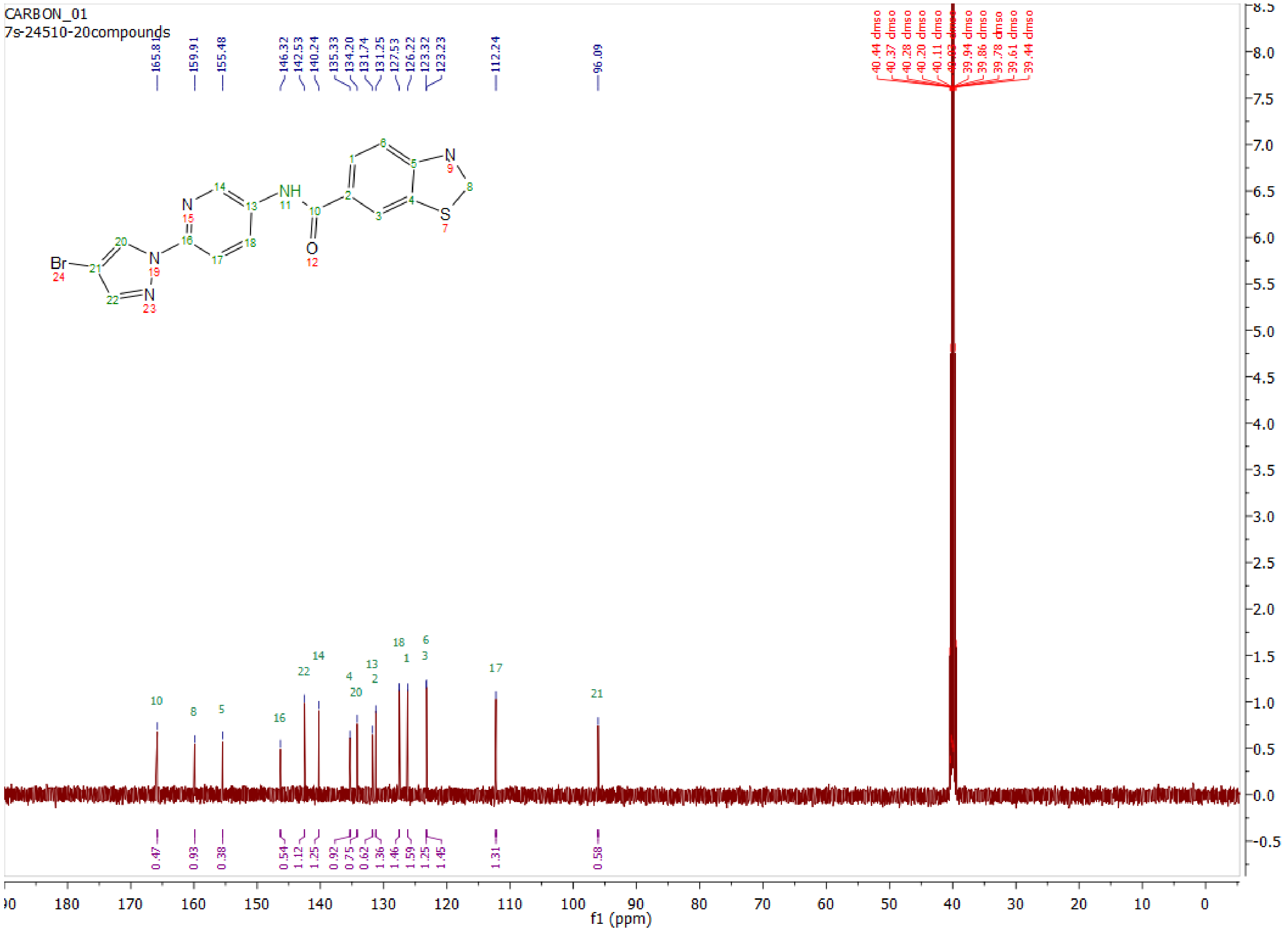
CARBON_01
7s-24510-20compounds
165.8
159.91
155.48
146.32
142.53
140.24
135.33
134.20
131.74
131.25
127.53
126.22
123.32
123.23
112.24
96.09
40.44 dmso
40.37 dmso
40.28 dmso
40.20 dmso
40.11 dmso
39.94 dmso
39.86 dmso
39.78 dmso
39.61 dmso
39.44 dmso
Br
NH
O
N
S
f1 (ppm)

Compound 10

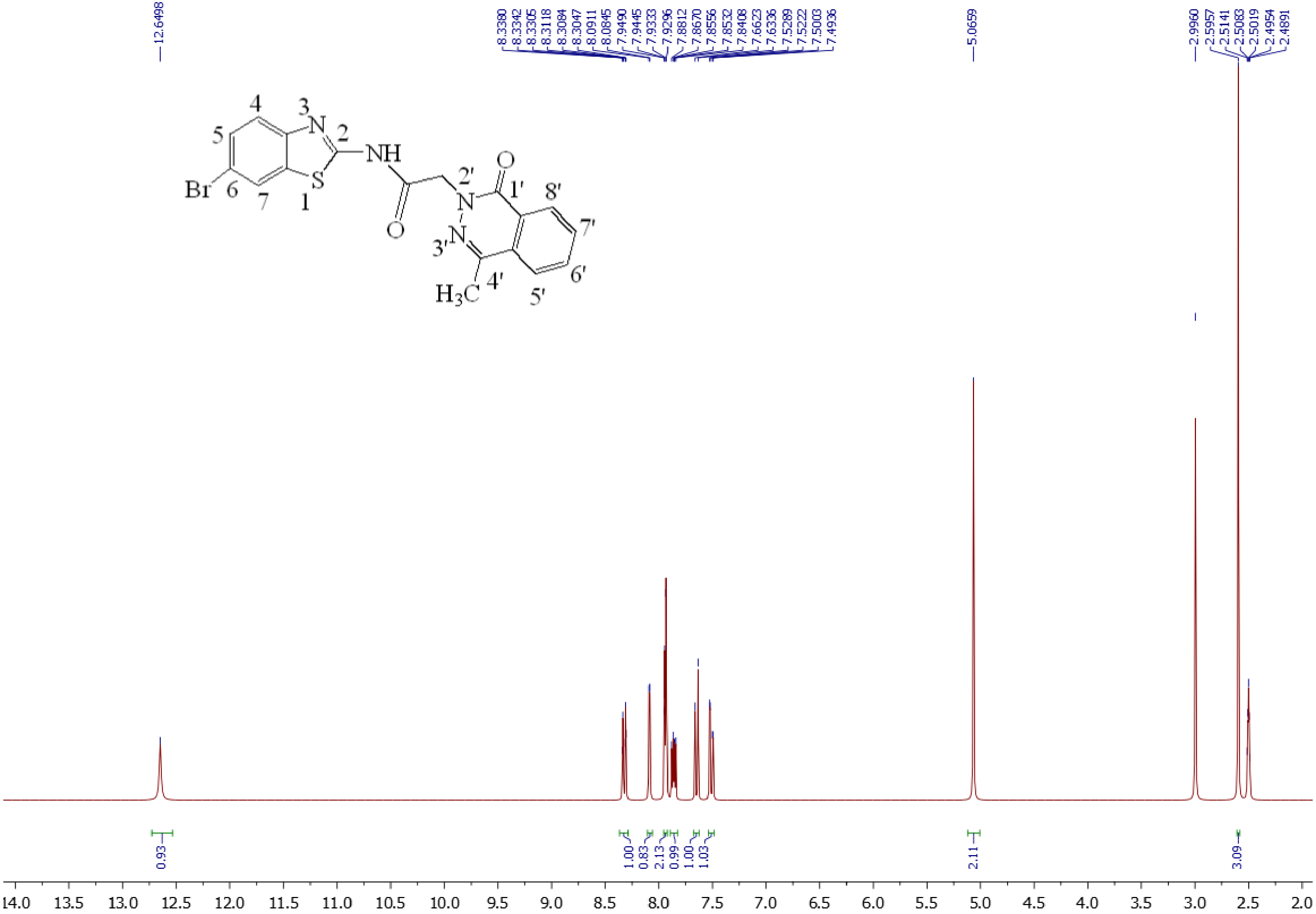

12.6498
8.3380
8.3342
8.3305
8.3118
8.3084
8.3047
8.0911
8.0845
7.9490
7.9445
7.9333
7.9296
7.8812
7.8670
7.8556
7.8532
7.8408
7.6623
7.6336
7.5289
7.5222
7.5003
7.4936
5.0659
2.9960
2.5957
2.5141
2.5083
2.5019
2.4954
2.4891
Br
NH
O
N
S
H_3C
0.93
1.00
0.83
2.13
0.99
1.00
1.03
2.11
3.09
14.0 13.5 13.0 12.5 12.0 11.5 11.0 10.5 10.0 9.5 9.0 8.5 8.0 7.5 7.0 6.5 6.0 5.5 5.0 4.5 4.0 3.5 3.0 2.5 2.0

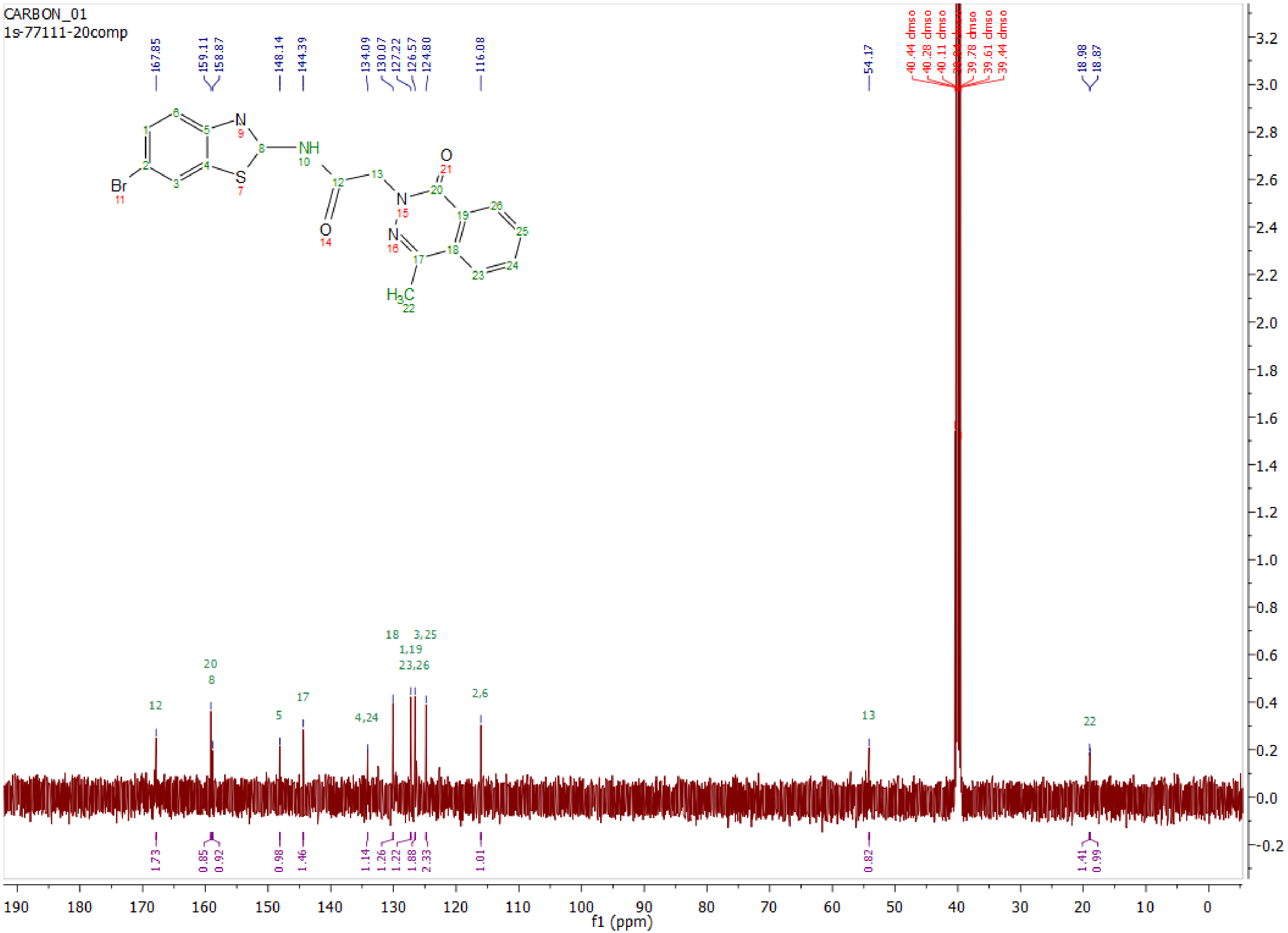
CARBON_01
1s-77111-20comp
167.85
159.11
158.87
148.14
144.39
134.09
130.07
127.22
126.57
124.80
116.08
54.17
40.44 dmso
40.28 dmso
40.11 dmso
39.78 dmso
39.61 dmso
39.44 dmso
18.98
18.87
Br
NH
O
N
S
H3C
12
20
8
5
17
4,24
18
1,19
23,26
3,25
2,6
13
22
1.73
0.85
0.92
0.98
1.46
1.14
1.26
1.22
1.88
2.33
1.01
0.82
1.41
0.99
f1 (ppm)
